# Relationship between job resources and job embeddedness among tertiary-level public hospital nurses: parallel mediating roles of work–family conflict and work–family enrichment

**DOI:** 10.3389/fpubh.2025.1527511

**Published:** 2025-06-02

**Authors:** Yujie Zhang, Shanyan Lei, Fang Yang

**Affiliations:** ^1^School of Humanities and Management, Zhejiang Chinese Medical University, Hangzhou, Zhejiang, China; ^2^The First Affiliated Hospital, Zhejiang University School of Medicine, Hangzhou, Zhejiang, China

**Keywords:** nurses, job resources, work–family conflict, work–family enrichment, job embeddedness, mediating effect, China

## Abstract

**Objective:**

This study explored the relationship between job resources and job embeddedness among tertiary-level public hospital nurses and the mediating role of work–family conflict and work–family enrichment.

**Methods:**

Data were collected from 1,420 nurses at five tertiary-level public hospitals in China. Measurements included job resources, work–family conflict, work–family enrichment, and job embeddedness. A descriptive analysis, *t*-test, one-way analysis of variance, hierarchical multiple regression analysis, and structural equation model were used to analyze the data.

**Results:**

Nurses’ job embeddedness was 23.57 ± 5.99. Job embeddedness for nurses varied according to age, education, employment type, years of service, work intensity, and health status (all *p*-values < 0.05). Nurses’ job resources were positively associated with job embeddedness (*β* = 0.214, *p* < 0.01). Nurses’ work–family conflict and work–family enrichment had a parallel mediating effect between job resources and job embeddedness, with mediating effect values of 0.120 and 0.044, respectively (*p* < 0.01). Regarding the specific dimensions of job resources, social supports, and skill diversity have the largest coefficients on job embeddedness (*β* = 0.288, *β* = 0.374), and both have direct and indirect association with job embeddedness.

**Conclusion:**

Nurses’ job resources were positively associated with job embeddedness directly and indirectly through the parallel mediating roles of work–family conflict and enrichment. Emphasis should be placed on the dimensions of social support and skill diversity within job resources, as these aspects are more likely to enhance nurses’ job embeddedness.

## 1 Introduction

In organizational behavior research, talent retention has long been a topic of significant academic interest. In today’s dynamic and competitive work environment, job embeddedness has increasingly attracted significant scholarly attention as a crucial metric for evaluating the connection between employees and organizations. The concept of job embeddedness, introduced in 2001, has progressively evolved into a pivotal theoretical framework for analyzing employee retention behaviors. It delineates the degree of attachment employees experience towards an organization and the challenges they encounter when contemplating departure (1). Mitchell (1) described job embeddedness as a web of social relationships; individuals are inextricably linked to one another in this web as network nodes, and everyone is embedded in it in various combinations. This concept provides a novel framework for comprehending the intricate dynamics between employees and organizations. In contrast to conventional research, which primarily focuses on turnover intentions or retention willingness, job embeddedness underscores the “rootedness” of employees within an organization. This rootedness extends beyond mere emotional attachments to the organization, encompassing profound connections with colleagues, the community, and opportunities for career development.

Nurses constitute an essential and pivotal component of the healthcare system. The intricate and demanding nature of their work environment significantly contributes to elevated turnover rates within the profession. In the context of China, the extent of job embeddedness plays a crucial role in determining nurses’ willingness to commit to long-term careers within the healthcare sector. Current research on job embeddedness predominantly examines its relationship with retention and turnover intentions (2, 3). Empirical evidence suggested that employees with high levels of job embeddedness are more inclined to remain with their current employer. A meta-analysis synthesizing data from 47 studies across seven countries found a moderate negative correlation between nurses’ job embeddedness and their turnover intentions (4). Moreover, findings indicate that nurses’ job embeddedness is closely related to key performance metrics, including work performance (5), attendance (6), and the incidence of nursing errors (7). For example, El-Sayed’s study (7) revealed a significant negative and moderate correlation between nurses’ job embeddedness and perioperative nursing errors. As the highest-level medical institutions in China, tertiary public hospitals are medical technology centers that combine medical treatment, prevention, teaching, and research. They are an important part of the current healthcare industry in China and are closely related to residents’ health interests and safety. Therefore, it is crucial to determine the degree of job embeddedness among nurses in tertiary public hospitals in China and explore the factors influencing job embeddedness.

A review of the existing literature reveals that research on job embeddedness among nurses is relatively limited. Notably, few studies examine the impact of job resources on job embeddedness from a holistic perspective, as most existing research focuses on specific, isolated dimensions of job resources. Given the distinctive characteristics of the nursing profession, there is a significant interaction between work and family domains, resulting in both positive and negative reciprocal influences. Importantly, few studies have explored the relationship between job resources and job embeddedness through the framework of the work–family interface. To address this research gap, work–family conflict (WFC) and work–family enrichment (WFE) are proposed as mediating variables. Based on existing research and theoretical frameworks, this study examines job embeddedness among nurses employed in public hospitals within China’s healthcare sector. The primary objective is to analyze the relationship between job resources and job embeddedness, while investigating whether WFC and WFE function as mediators in this relationship.

This investigation seeks to broaden the application of theories such as the JD-R model and to enhance the understanding of job embeddedness. By exploring the influence of job resources on nurses’ job embeddedness, this study aims to contribute to the development of a more comprehensive theoretical framework. Additionally, it provides guidance for hospital human resource management departments, enabling them to focus more on flexible management and comprehensive support in policy formulation and implementation. This can enhance the rational allocation of job resources, improve retention rates among healthcare professionals, and indirectly elevate the overall quality of medical services, thereby offering better care to patients.

## 2 Literature review and research hypotheses

### 2.1 Job resources and job embeddedness

According to the Job Demands-Resources (JD-R) model, two pivotal psychological processes operate within the workplace. The first is the health impairment process, driven by excessive job demands, which can lead to negative outcomes (8, 9). The second critical process is the motivational process, wherein individuals endowed with adequate job resources experience favorable work outcomes, such as increased work engagement and enhanced performance (8, 9). Notably, existing research has largely focused on the negative aspects of work, including job stress, excessive demands, and related challenges (10–12), while relatively little attention has been given to the positive effects of work. Within the JD-R framework, job resources encompass resources derived from social, material, and organizational domains (13, 14), including social support, organizational support, organizational justice, decision-making autonomy, and performance feedback.

Currently, research on the impact of job resources primarily concentrate on work engagement (15) and job performance (16). For example, Tu’s study (15) demonstrated a positive correlation between job resources and work engagement. Nevertheless, the comprehensive influence of job resources on job embeddedness remains insufficiently explored. Some studies propose job resources as a potential strategy for enhancing organizational stability and mitigating turnover rates. In particular, within the nursing sector, research has examined specific dimensions of job resources, such as social support (17), job skills (18), rewards, and job control (19), to elucidate their effects on job embeddedness. Yun’s study (17) demonstrated a significant association between job skills, social support, and nurses’ job embeddedness. Meanwhile, El-Gazar’s research (19) on Egyptian nurses indicated that job control significantly affects nurses’ job embeddedness, whereas rewards are not a predictive factor. This study aims to investigate the overall influence of job resources on nurses’ job embeddedness, as well as the relationships between specific dimensions of job resources and nurses’ job embeddedness. Based on this, the following research hypotheses are proposed:

*Hypotheses 1*: Job resources are associated with job embeddedness among tertiary-level public hospital nurses.

*Hypotheses 2*: Dimensions of job resources (social support, skill diversity, decision making, rewards, job control) are associated with job embeddedness among tertiary-level public hospital nurses.

### 2.2 Parallel mediating roles of work–family conflict and work–family enrichment

Work and family form the foundation of adult life, encompassing two primary domains where individuals allocate substantial time and energy, thereby exerting a profound influence on their professional and personal lives. In China, hospitals occupy a pivotal role within the healthcare system. Nurses, as essential components of medical teams, undertake diverse tasks, including routine patient care, health monitoring, medication administration, and health education. The complexity of their daily duties is frequently associated with elevated stress levels. Since work is essential for earning a living, nurses dedicate most of their time and energy to their careers. Family remains a crucial part of their personal lives. Most nurses are women, and traditional Chinese beliefs place significant family duties on them, such as raising children and caring for the older adult (20). Role theory (21) suggests that when time and energy are limited, the demands of work and family can clash, causing an imbalance that affects personal functioning. This conflict stems from the opposing responsibilities of work and family roles. Work–family conflict (WFC) is characterized as an inter-role conflict arising from the mutually incompatible pressures exerted by work and family roles. This conflict primarily stems from time constraints and demands intensity, which hinder individuals from effectively fulfilling the obligations of both roles. This conflict is bidirectional, encompassing both work interfering with family responsibilities and family obligations impeding work performance (22, 23). The first dimension refers to situations where work-related time commitments, stress, or responsibilities hinder the fulfillment of family duties. In contrast, the second dimension describes instances where family demands encroach upon the performance of professional responsibilities (22, 23).

The Work–Family Boundary Theory suggests that work and family constitute two relatively autonomous yet interconnected domains, characterized by flexibility, permeability, and compensatory dynamics (24). Owing to variations in the mechanisms and modes of interaction between these domains, alongside work–family conflict, diverse forms of work–family relationships have emerged, including WFE. WFE denotes the extent to which an individual’s engagement in one domain (work or family) generates benefits (e.g., development, affect, capital, efficiency) that positively enhance functioning in the other domain. WFE offers an optimistic viewpoint in the examination of work–family dynamics, highlighting the “synergistic promotion effect” that occurs between these two domains. This concept encompasses two aspects: work enriching family and family enriching work (25). The first dimension pertains to the acquisition of knowledge, skills, emotions, and social resources by individuals through their professional activities, which can subsequently enhance their effectiveness or wellbeing in familial roles. The fundamental mechanism of this dimension is predominantly evident in elements such as skill transfer, emotional spillover, and resource accumulation. For example, the emergency response skills that nurses develop in their clinical practice can enable them to manage family emergencies with greater composure. Similarly, the positive emotions derived from professional accomplishments can foster increased patience and tolerance in familial interactions; for instance, the sense of fulfillment from successfully saving a critically ill patient may lead a nurse to interact with her family members more compassionately. Conversely, the second dimension relates to the resources and emotional support garnered from the family domain, which can, in turn, enhance work performance and facilitate career advancement.

According to social exchange theory, individuals are likely to reciprocate by demonstrating attitudes and behaviors that align with the benefits they have received, particularly when they perceive that their organization has offered advantageous resources to them or their families (26). In the context of nursing, the perception of job resources provided by the organization can prompt nurses to reciprocate by mitigating conflict or enhancing family support (27), thereby fostering a “resource input-organizational commitment” cycle. Meanwhile, according to the JD-R theory, the influence of work on individuals is mediated through a motivational process, wherein sufficient access to job resources leads to favorable outcomes. Analogous to the research examining the correlation between job resources and job embeddedness, recent research has increasingly concentrated on examining the specific dimensions of job resources and their effects on WFC and WFE. For example, Ghislieri et al. (28) found that organizational support is negatively correlated with WFC and positively correlated with WFE among nurses. A meta-analysis further established a strong association between social support and WFC, indicating that social support significantly mitigates WFC (29). Another study focusing on security screening personnel identified supervisor support as a key job resource that significantly predicts WFC. Research focusing on Chinese nurses has shown that ample job resources can alleviate conflicts between family responsibilities and work demands, such as workloads or time pressures. Notably, implementing a flexible scheduling system can aid nurses in managing family obligations, thereby diminishing the impact of work on family life (30). Moreover, Wang’s research (31) highlighted that job flexibility serves as a significant moderating factor in the relationship between employees’ emotional experiences and WFE.

Initial research predominantly concentrated on the conflict between work and family roles, highlighting the adverse effects resulting from the interference between these roles. However, with the advancement of research and the advent of positive psychology, scholars have increasingly focused on the beneficial interactions between work and family. According to the hypothesis of limited individual resource allocation (32), work and family demands compete for an individual’s finite resources, potentially leading to psychological conflict and varying energy levels. This competition could subsequently reduce the extent of an individual’s job embeddedness. The research conducted by Dukhaykh (33) revealed a negative correlation between WFC and job embeddedness, suggesting that women facing conflicts between work and family obligations encounter difficulties in managing extensive work demands. This difficulty impacts their job embeddedness. Regarding the relationship between WFE and job embeddedness, harmony between work and family life generally leads to increased employee satisfaction with their organization, fostering a stronger connection with both the organization and colleagues, thereby implying a higher degree of job embeddedness. Consequently, WFC and WFE may function as parallel mediators concerning job resources and job embeddedness. Based on these findings, the following research hypotheses are proposed:

*Hypotheses 3:* WFC mediates the relationship between job resources and job embeddedness among tertiary-level public hospital nurses.

*Hypotheses 4:* WFE mediates the relationship between job resources and job embeddedness among tertiary-level public hospital nurses.

*Hypotheses 5:* WFC mediates the relationship between dimensions of job resources (social support, skill diversity, decision making, rewards, job control) and job embeddedness among tertiary-level public hospital nurses.

*Hypotheses 6:* WFE mediates the relationship between dimensions of job resources (social support, skill diversity, decision making, rewards, job control) and job embeddedness among tertiary-level public hospital nurses.

**Figure 1:**
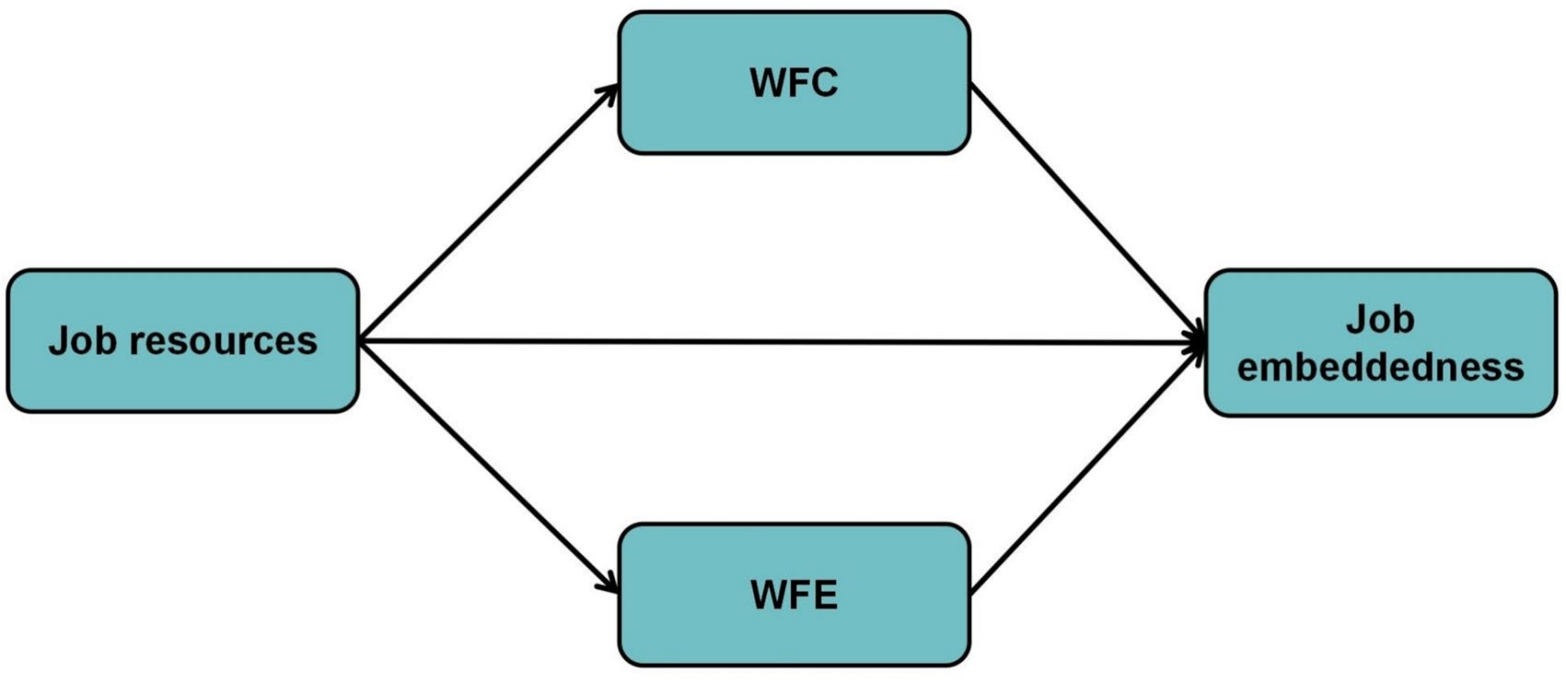
Hypothesis model.

Figure 1 shows the conceptual model used in this study.

## 3 Materials and methods

### 3.1 Study design

A cross-sectional study design was used to explore the parallel mediating roles of WFC and WFE in the relationship between job resources and job embeddedness.

### 3.2 Participants and data collection

The recruitment process was conducted from October to December 2021 in Weifang City, Shandong Province. Five public hospitals within Weifang City were randomly selected as sampling units. The data collection process strictly complied with the core principles of data acquisition and privacy protection (34, 35). Following approval from hospital administrators, researchers, with the collaboration of head nurses, engaged with potential participants. Departments were randomly chosen from six principal systems within the selected hospitals: internal medicine, surgery, gynecology, pediatrics, emergency, and ICU, in accordance with the principle of a 30% equal proportion. Ultimately, nurses on duty who satisfied the inclusion criteria on the sampling day were selected as study participants. The inclusion criteria were as follows: (1) currently registered nurses; (2) a minimum of 1 year of clinical nursing experience; (3) provision of informed consent and voluntary participation. The exclusion criteria included: (1) trainee or intern nurses; (2) nurses not on duty during the survey period. Intern nurses are generally in the midst of their training, and their professional skills and judgment may not yet be fully developed. As a result, the data pertaining to intern nurses may not accurately represent the conditions experienced by practicing nurses. Similarly, nurses who are not currently on duty are not engaged in the ongoing nursing practice within the clinical environment, rendering their data potentially irrelevant to the research objectives. Consequently, we excluded both intern nurses and off-duty nurses from our study.

The questionnaire utilized in this study was a paper-based survey composed in Chinese. To ensure data accuracy, rigorous quality control measures were implemented. (1) Questionnaire Design: The research team conducted an extensive review of both domestic and international literature, followed by multiple group discussions to refine and enhance the questionnaire content. This process ensured its comprehensive applicability and specificity. (2) Formal Survey: The survey was administered by graduate students who had received standardized training. Nurses independently completed the questionnaires with assistance from the survey administrators, and the completed questionnaires were collected on-site. Survey personnel used consistent language to clarify questions for respondents. To ensure the quality of the questionnaires, a system of self-checks and mutual checks among the survey administrators was employed. (3) Data Entry: The completed questionnaires were first reviewed to identify and exclude invalid responses. Data entry was performed using a double-entry method coupled with logical error checking to ensure data accuracy and integrity. The criteria for excluding questionnaires included the presence of evident logical inconsistencies, incomplete answers, patterned responses, or multiple answers to a single question. Participants were assured of the anonymity of their responses and were informed that declining to participate would not result in any adverse consequences.

### 3.3 Sample size

The sample size was determined using the formula for cross-sectional studies: *N* = (U_*α*/2_ *σ*/*δ*)^2^ (36). Based on existing literature, the standard deviation for nurse work engagement is 1.06 (37). The significance level α was set at 0.05, with U_α/2_ = 1.96. Allowing for a permissible error *δ* = 0.1, the calculation yields *N* = (1.96 × 1.06/0.1)^2^ ≈ 432. Considering a 20% rate of invalid responses, the minimum required sample size was 518. To enhance the representativeness and reliability of the findings, while concurrently considering the sample size requirements of the structural equation model, a large number of questionnaires were distributed. This strategy aimed to minimize potential bias arising from variations in hospital characteristics and department types. A total of 1,542 questionnaires were distributed. After excluding those with common errors or evident inconsistencies, 1,420 valid responses were obtained, resulting in a response rate of 92.1%.

### 3.4 Instruments

The questionnaire is comprised of the following components: exposure variables (the primary focus of this study), outcome variables, and a range of confounding variables.

#### 3.4.1 Exposure: job resources

Job resources were assessed using the Job Resources Scale. The Job Resources Scale prepared by Li (38) was used to investigate participants’ job resources. The scale includes five dimensions: social support, rewards, skill diversity, job control, and participation in decision-making, totaling 15 entries. Each entry was scored on a 5-point Likert scale from 1.0 to 5.0, with entries 1–5 and 10–13 being reverse scored. Higher scores indicated more job resources. The Cronbach’s *α* coefficients for the five dimensions of the original scale ranged from 0.61 to 0.82, demonstrating acceptable internal consistency reliability. The factor loadings for the items on the original scale exceeded 0.50, with the total variance explained amounting to 65.58%. Additionally, the fit indices for the five-factor model were satisfactory, demonstrating robust structural validity.

#### 3.4.2 Outcome 1: job embeddedness

This scale was developed by Crossley et al. (39) and is widely used in China (5, 40–43). The scale is unidimensional, with seven entries. The scale was scored on a 5-point Likert scale, with higher scores indicating higher levels of job embeddedness. The Cronbach’s *α* coefficient for the scale in this study was 0.872. The original scale demonstrated robust construct validity, as evidenced by a comprehensive global assessment.

#### 3.4.3 Outcome 2: WFC and WFE

WFC and WFE were assessed using the Work–Family Relationship Scale. The Work–Family Relationship Scale developed by Grzywacz (44) and translated by Zeng (45) was used to investigate the work–family relationships of the study participants. This scale includes two subscales: WFC and WFE. The WFC subscale includes two dimensions, work-to-family conflict (that is, work factors negatively affecting family life) and family-to-work conflict (that is, family factors negatively affecting work life), totaling eight entries. The WFE subscale includes two dimensions, work-to-family enrichment and family-to-work enrichment (work factors enriching family life and family factors enriching work life), totaling six entries. The entries are scored on a 5-point Likert scale from 1.0 to 5.0, with higher scores indicating higher levels of WFC and WFE. The internal consistency reliability, test–retest reliability, and composite reliability for the four dimensions of the original scale were observed to range from 0.68 to 0.77, 0.75–0.83, and 0.69–0.83, respectively, indicating a satisfactory level of reliability. The indicators for the four-factor model of the original scale were deemed satisfactory, and the correlation coefficients with criterion variables were statistically significant. These results confirmed the scale’s construct validity and criterion validity.

#### 3.4.4 Confounding variables: demographic and sociological variables

Potential confounding factors, including socio-demographic characteristics and job-related variables, were collected using a self-designed demographic questionnaire. It included questions on sex, age, education level, employment type, years of service, health status, and work intensity.

### 3.5 Data analysis

SPSS 22.0 and AMOS software were used for the statistical analysis of the data. In the present analyses, nurses with incomplete data were excluded. To mitigate potential common method bias arising from self-reported measures collected at a single time point, Principal Component Analysis (PCA) was employed. This step is essential for detecting any singular underlying structure that could artificially enhance the relationships among variables. PCA was chosen for its ability to elucidate data dimensions and detect variance attributable to a common method factor.

To confirm the suitability of the statistical tests employed, we evaluated the normality of the variables through assessments of skewness and kurtosis. Data conforming to a normal distribution were described as mean ± standard. Data that did not conform to a normal distribution were described using the median (M) and the 0th to 100th percentiles (*P_0_–P_100_*). Count data were described by frequency and percentage. Internal consistency reliability and composite reliability (CR) were used to assess the reliability of the scales, as measured by Cronbach’s alpha and CR values, respectively. Confirmatory factor analysis was used to assess the construct validity of the scales. The convergent validity of the scales was assessed mainly by average variance extracted (AVE) and factor loading values.

A *t*-test was used to compare the means between two groups, and a one-way analysis of variance was used to compare the means between multiple groups. For variables that deviated from a normal distribution, non-parametric tests were utilized for analytical purposes. In univariate analysis, sociodemographic characteristic demonstrating a significant difference with the dependent variable was incorporated as a covariate in subsequent analyses. Pearson’s correlation analysis was used to explore the correlation between variables. Parallel mediation tests were performed using the SPSS macro PROCESS program, and Model 4 was chosen as the base model. During the analytical procedure, a bootstrap method utilizing 5,000 resamples was implemented. The results were considered significant if the 95% confidence interval’s upper and lower bounds excluded zero. A structural equation model (SEM) was conducted to examine the effect of dimensions of job resources on nurses’ job embeddedness. For the two-sided test level, *α* = 0.05.

## 4 Results

### 4.1 Demographic characteristics of participants

The majority of survey participants were aged between 31 and 40 years, comprising 44.4% of the sample, with a mean age of 33.50 ± 7.60 years. The majority were women (95.6%), had a bachelor’s degree or above (89.2%), and worked as contract-based personnel (83.2%). The length of service primarily ranged from 6 to 10 years, accounting for 32.0%. Additionally, 38.7% had good general health. A total of 52.0% of the nurses perceived their work intensity to be high (Table 1).

**Table 1:** Univariate analysis of job embeddedness based on demographic characteristics.

Characteristic	*N* (%)	*M ± SD*	*t/F*	*p*
Total score		23.57 ± 5.99		
Average score		3.37 ± 0.86		
Sex				
Male	63(4.4)	23.94 ± 5.61	0.50	0.62
Female	1,357(95.6)	23.55 ± 6.01		
Age(year)				
≤30	548(38.6)	23.66 ± 5.77	3.58	0.013
31–40	630(44.4)	23.12 ± 6.19		
41–50	195(13.7)	24.40 ± 5.86		
≥51	47(3.3)	25.15 ± 5.94		
Educational level				
Junior college and below	153(10.8)	24.35 ± 5.73	7.05	0.001
Undergraduate	1,251(88.1)	23.41 ± 6.01		
Postgraduate or above	16(1.1)	28.44 ± 4.32		
Employment type				
Permanent	238(16.8)	24.62 ± 5.85	8.85	0.003
Contract-based/Personnel agent	1,182(83.2)	23.36 ± 6.00		
Years of service				
≤5	386(27.2)	23.97 ± 5.72	4.42	0.001
6–10	455(32.0)	22.84 ± 5.98		
11–15	232(16.3)	23.19 ± 6.47		
16–20	135(9.5)	23.69 ± 5.94		
≥21	212(14.9)	24.74 ± 5.82		
Healthy status				
Very unhealthy	35(2.5)	20.11 ± 6.88	19.95	<0.001
Less healthy	333(23.5)	22.18 ± 6.25		
General healthy	550(38.7)	23.16 ± 5.66		
More healthy	430(30.3)	24.90 ± 5.66		
Very healthy	72(5.1)	26.92 ± 5.72		
Work intensity				
Very low/rarely	34(2.4)	28.00 ± 5.60	12.23	<0.001
Commonly	419(29.5)	24.08 ± 5.47		
Higher	739(52.0)	23.54 ± 5.95		
Very high	228(16.1)	22.07 ± 6.64		

In this study, outliers were identified in two variables: age and length of service. These outliers surpassed the legal retirement age and exhibited unusually long durations of service according to Chinese standards. Consequently, analyses involving these variables were conducted both with and without the outliers to facilitate a sensitivity analysis. The results demonstrated that the presence of outliers did not affect the outcomes. Therefore, the outliers were retained in all subsequent analyses.

### 4.2 Assessment of the measurement instruments reliability and validity

The minimum Cronbach’s *α* and CR values for each scale were both 0.757, exceeding the recommended value of 0.7, indicating good internal consistency reliability and composite reliability (Table 2). The minimum AVE value of each scale was 0.542, which exceeded the recommended value of 0.5. And the factor loadings were all greater than 0.5, indicating good convergent validity (Table 2). The results of the confirmatory factor analysis showed that the scales of job resources, WFC, WFE, and job embeddedness had a five-factor, two-factor, two-factor, and one-factor structure, respectively (Supplementary material). The model fit indexes generally reached the reference values, and the model fit was good. Additionally, all factor loadings were greater than 0.5, indicating good construct validity (Table 2).

**Table 2:** Assessment of the measurement instruments reliability and validity.

Index	Normality		Cronbach’s α	*CR*	*AVE*	*GFI*	*NFI*	*IFI*	*PNFI*	*PCFI*
Reference value	Skewness	Kurtosis	>0.7	>0.7	>0.5	>0.9	>0.9	>0.9	>0.6	>0.6
Job resources	0.245	−0.119				0.943	0.941	0.947	0.717	0.722
Social support	−0.616	0.573	0.865	0.868	0.569					
Rewards	−0.118	−0.876	0.872	0.882	0.791					
Skill diversity	−0.442	−0.051	0.757	0.757	0.609					
Job control	0.367	−0.255	0.829	0.828	0.616					
Decision making	0.156	−0.498	0.865	0.876	0.706					
Work–family conflict	0.083	0.242				0.950	0.971	0.973	0.763	0.765
Work-to-family conflict	−0.312	−0.120	0.922	0.925	0.756					
Family-to-work conflict	0.600	0.132	0.930	0.931	0.771					
Work–family enrichment	0.019	0.449				0.942	0.944	0.946	0.630	0.631
Work-to-family enrichment	−0.002	0.214	0.847	0.856	0.665					
Family-to-work enrichment	−0.310	0.210	0.865	0.868	0.689					
Job embeddedness	−0.205	−0.242	0.873	0.891	0.542	0.904	0.910	0.912	0.520	0.521

As shown in Table 2, the absolute values of the skewness and kurtosis coefficients for all variables lie within the acceptable range. Consequently, it can be inferred that the data derived from the measurement instruments exhibit a normal or near-normal distribution.

### 4.3 Common method bias test

Common method bias has the potential to systematically inflate or distort the observed correlations between variables. For example, the relationship between independent and dependent variables may partly arise from the uniformity of the data source or measurement techniques, rather than reflecting a true causal connection. Consequently, this can undermine the validity of the findings, thereby complicating the generalization of the research results to other measurement instruments or research contexts. A principal component analysis yielded a single factor, explaining 29.109% of the total variance among the variables, which is below the commonly referenced threshold of 50% (46). This result suggests that while some common method variance is present, it does not predominate the responses and, consequently, does not unduly affect the relationships between the study variables.

### 4.4 Differences in variables based on demographic characteristics

A comparison of the job embeddedness scores of nurses with different ages, education levels, employment types, years of service, work intensity, and health statuses showed statistically significant differences (all *p*-values < 0.05) (Table 1).

### 4.5 Correlation analysis of job resources, WFC, WFE, and job embeddedness

The results of Pearson’s correlation analysis showed that job resources, WFC, and WFE were correlated with job embeddedness (*p* < 0.01) (Table 3). Based on the nurses’ scores on the Job Embeddedness Scale, 71 nurses were selected as the “low job embeddedness” group by 5% from low to high, and 71 nurses were selected as the “high job embeddedness” group by 5% from high to low, with reference to the relevant criteria (44). The differences in job resources, WFC, and WFE between high and low job embeddedness levels were analyzed. The results showed that there were significant differences at high and low job embeddedness levels (*p* < 0.001) (Table 3).

**Table 3:** Correlation analysis of job embeddedness, job resources, WFC and WFE.

Variables	*M*	*SD*	Job embeddedness(*r*)	*p*	High-level job embeddedness (*N* = 71)	Low-level job embeddedness (*N* = 71)	*t*	*p*
Job resources	51.50	8.92	0.401	<0.01	62.28 ± 8.48	44.77 ± 7.83	12.78	<0.001
Social support	20.57	3.31	0.307	<0.01	23.17 ± 3.07	18.44 ± 3.69	8.30	<0.001
Rewards	6.59	2.30	0.212	<0.01	7.99 ± 2.46	5.61 ± 2.35	5.90	<0.001
Skill diversity	7.41	1.87	0.078	<0.01	8.49 ± 1.87	7.82 ± 1.80	2.19	0.030
Job control	7.48	2.98	0.302	<0.01	10.00 ± 3.85	5.11 ± 2.18	9.31	<0.001
Decision making	9.45	2.97	0.344	<0.01	12.63 ± 2.96	7.80 ± 3.40	9.04	<0.001
WFC	22.53	6.54	−0.398	<0.01	15.58 ± 7.15	29.19 ± 7.05	−11.42	<0.001
WFE	20.27	4.38	0.280	<0.01	24.28 ± 5.60	18.25 ± 4.54	7.05	<0.001

### 4.6 The mediating effect of WFC and WFE on job resources and job embeddedness

**Figure 2:**
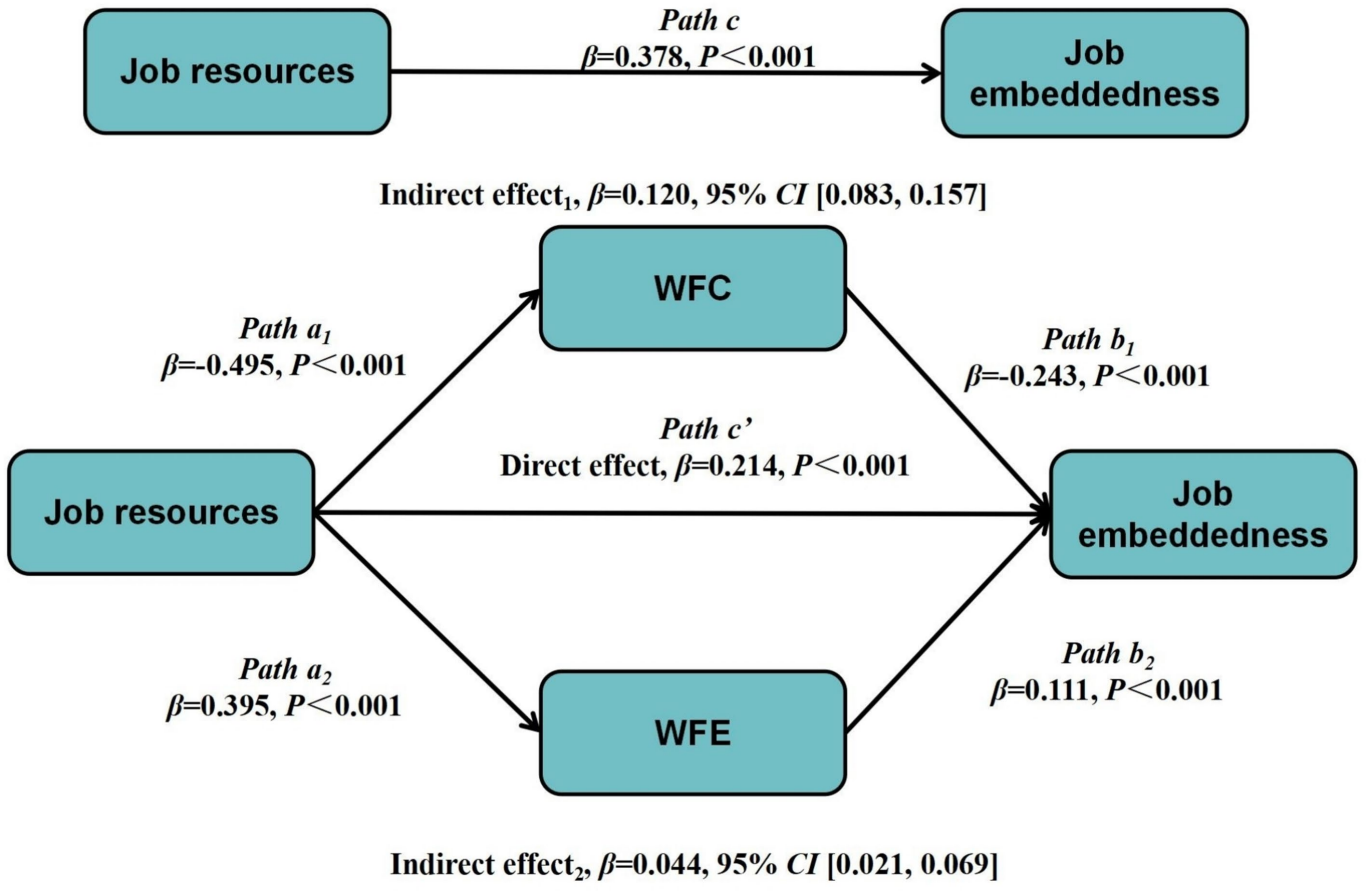
Mediating models of WFC and WFE between job resources and job embeddedness.

The PROCESS program developed by Preacher and Hayes (47) was used in this study. The bootstrap method for bias correction was used to calculate the mediation effect. The sample size was 5,000. The confidence interval was set at 95%. Mediating effects were analyzed using age, employment type, years of service, health status, and work intensity as control variables, job resources as independent variables, WFC and WFE as mediating variables, and job embeddedness as the dependent variable. The tests of mediating roles showed that job resources were significantly positively associated with job embeddedness (*β* = 0.214, *p* < 0.001), WFC was significantly negatively associated with job embeddedness (*β* = −0.243, *p* < 0.001), and WFE was significantly positively associated with job embeddedness (*β* = 0.111, *p* < 0.001). Meanwhile, job resources were significantly negatively associated with WFC (*β* = −0.495, *p* < 0.001) and significantly positively associated with WFE (*β* = 0.395, *p* < 0.001) (Table 4; Figure 2). No statistically significant interactions were identified among the primary covariates or control variables. Through the application of regression analysis, it was determined that the tolerance and variance inflation factor (VIF) values for each model were within the acceptable range, thereby indicating the absence of multicollinearity issues.

**Table 4:** Results of hierarchical regression analysis.

Model	Variables		Fit indices			Coefficient significance			
	Dependent variable	Independent variable	*R^2^*	*F*	*p*	*β*	Standard Error	*t*	*p*
Model 1	Job embeddedness	Job resources	0.172	42.013	<0.001	0.378	0.019	13.634	<0.001
Model 2	WFC	Job resources	0.418	144.582	<0.001	−0.495	0.023	−21.279	<0.001
Model 3	WFE	Job resources	0.215	55.277	<0.001	0.395	0.013	14.652	<0.001
Model 4	Job embeddedness	WFC	0.218	43.543	<0.001	−0.243	0.028	−7.880	<0.001
		WFE				0.111	0.036	4.174	<0.001
		Job resources				0.214	0.022	6.551	<0.001

The coefficient of the direct path from job resources to job embeddedness was 0.214, with the direct coefficient accounting for 56.61% of the total coefficient value. The coefficient for the indirect paths of WFC and WFE were 0.120 and 0.044, respectively, with indirect coefficient accounting for 31.75 and 11.64% of the total coefficient, respectively. The upper and lower limits of the bootstrap 95% confidence intervals (CI) of the above paths did not contain zero, indicating that all paths were significant. These results indicate that nurses’ job resources can directly and positively associate with job embeddedness and indirectly predict job embeddedness through WFC and WFE (Table 5).

**Table 5:** Results of bootstrapping mediation effect examination.

Effect	Path	Effect coefficient	Standard Error	Bootstrap 95%*CI*		Proportion of effect
				Lower	Upper	
Total effect	Job resources → job embeddedness	0.378	0.028	0.323	0.432	100%
Direct effect	Job resources → job embeddedness	0.214	0.033	0.150	0.277	56.61%
Indirect effect		0.164	0.022	0.120	0.208	43.39%
Indirect effect1	Job resources → WFC → job embeddedness	0.120	0.019	0.083	0.157	31.75%
Indirect effect2	Job resources → WFE → job embeddedness	0.044	0.012	0.021	0.069	11.64%

### 4.7 The effect of dimensions of job resources on nurses’ job embeddedness

AMOS software was applied to further analyze the effects of job resource dimensions (social support, skill diversity, decision making, rewards, job control) on job embeddedness and whether WFC and WFE play mediating effects between them. The results of the model fitting show that comparative fit index/degrees of freedom (CMIN/DF) = 4.955, root mean square error of approximation (RMSEA) = 0.053, goodness of fit index (GFI) = 0.999, adjusted goodness of fit index (AGFI) = 0.969, normed fit index (NFI) = 0.998, relative fit index (RFI) = 0.951, incremental fit index (IFI) = 0.999 (Table 6). The results of the above indexes reach the ideal reference standard, which indicates that the model fits well, no additional adjustments to the model were made based on the modification index.

**Table 6:** The model fitting parameters.

Fit index	*CMIN/DF*	*GFI*	*AGFI*	*NFI*	*RFI*	*IFI*	*RMSEA*
Reference value	<5	>0.9	>0.9	>0.9	>0.9	>0.9	<0.08
Correction value	4.955	0.999	0.969	0.998	0.951	0.999	0.053

**Figure 3:**
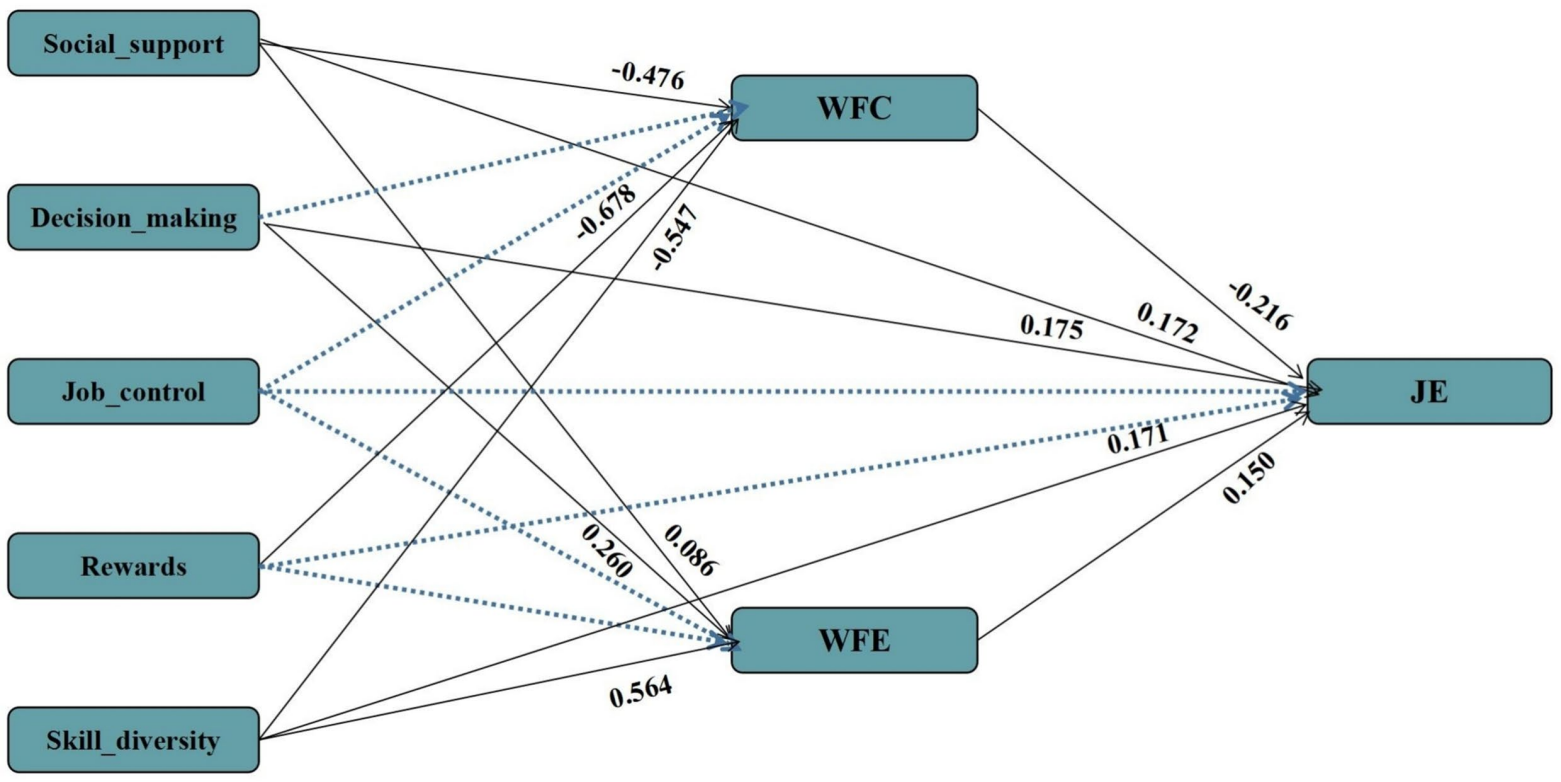
Structural equation model: the effect of dimensions of job resources on nurses’ job embeddedness.

The results show that social support and skill diversity have the largest coefficients on job embedding (*β* = 0.288, *β* = 0.374). Social support and skill diversity have a direct association with job embedding (*β* = 0.172, *β* = 0.171) and play an indirect effect on job embedding through WFC (*β* = 0.103, *β* = 0.118) and WFE (*β* = 0.013, *β* = 0.085). Decision-making also directly associates with job embeddedness (*β* = 0.175), but only indirectly through WFE (*β* = 0.039). Decision-making did not associate with WFC (*p* > 0.05) and could not indirectly associated with job embedding through WFC. Rewards did not have a direct association with job embedding. Rewards also did not associate with WFE and could not play an indirect role in job embedding through WFE but could play an indirect role in job embedding through WFC (*β* = 0.103, *β* = 0.118). On the other hand, job control does not directly associate with job embeddedness (*p* > 0.05) and does not indirectly associate with job embeddedness through WFC and WFE. The above results are reported in Table 7 and Figure 3.

**Table 7:** Path coefficients between variables.

Dependent variable	Independent variable	*SE*	*CR*	*P*	Direct effect	Indirect effect	Total effect
Job embeddedness	Social support	0.05	3.36	<0.001	0.172	–	0.288
	Skill diversity	0.07	2.61	0.01	0.171	–	0.374
	Decision making	0.07	2.45	0.01	0.175	–	0.214
	Rewards	0.06	1.26	0.21	0.079	–	0.146
	Job control	0.08	0.42	0.68	0.035	–	
	WFC	0.03	−7.59	<0.001	−0.216	–	
	WFE	0.04	4.05	<0.001	0.150	–	
WFC	Social support	0.05	−10.33	<0.001	−0.476	0.103	
	Skill diversity	0.06	−9.76	<0.001	−0.547	0.118	
	Rewards	0.06	−12.22	<0.001	−0.678	0.146	
	Decision making	0.07	−0.38	0.70	−0.025	–	
	Job control	0.08	−1.50	0.13	−0.117	–	
WFE	Social support	0.04	2.43	0.02	0.086	0.013	
	Skill diversity	0.04	13.11	<0.001	0.564	0.085	
	Rewards	0.04	0.51	0.61	0.022	–	
	Decision making	0.05	5.13	<0.001	0.260	0.039	
	Job control	0.06	1.08	0.28	0.064	–	

## 5 Discussion

The nurses’ job embeddedness score was 3.37 ± 0.86. Nurses’ job embeddedness was medium to high, a finding generally consistent with previous studies (18, 40, 48). This may be related to China’s active public hospital reforms in recent years. Nurses’ remuneration and job security have improved to a great extent. At the same time, nurses in tertiary public hospitals have better job remuneration packages and job stability than those in secondary hospitals, primary hospitals, private hospitals, or other occupations (49), which may have led to a higher level of job embeddedness. In addition, the traditional culture of China places a greater emphasis on collectivism than individualism, which may also play a key role in influencing the relevance of members within the collective and their embeddedness in the organization. However, while the status of job embeddedness among nurses was found to be reasonable, there is room for improvement. This may be because of a range of obstacles, such as doctor–patient conflicts, which are still prominent in China. In addition, nurses are busier than they were, and it is difficult to reconcile their work roles with their family roles (50).

The results of the study showed that the job embeddedness level was lower among nurses aged 31–40 years old and those with 6–15 years of work experience, with mean scores of 23.12 ± 6.19 and 22.84 ± 5.98, respectively. This may be because nurses in this age group are generally the backbone of the department and under pressure for daily nursing care, clinical teaching, and title promotion. At the same time, nurses in this age group also have heavier family responsibilities in terms of raising children and supporting the older adult. This may have resulted in lower job embeddedness among the nurses in this age group. A survey conducted among middle-aged male nurses in South Korea suggests that those in the mid-stage of their careers are contemplating transitioning to alternative job roles (51). The study identifies significant variations in job embeddedness levels among nurses employed in public hospitals under different contractual arrangements (*p* < 0.05), with contract-based and personnel agent nurses demonstrating comparatively lower levels of job embeddedness. Qin’s (52) research indicates that nurses on temporary contracts experience elevated stress levels relative to their counterparts on permanent contracts. Furthermore, contract-based and personnel agent nurses have relatively poor remuneration, benefits, titles, promotion chances, and participation in decision making (53). This may lead to a relatively low sense of professional belonging and a relatively low level of job embeddedness.

The findings of this study reveal significant variations in job embeddedness among nurses with different health statuses (*p* < 0.05). The findings of Aloisio (54) and Kamau (55) suggest that a nurse’s health level significantly influences their organizational commitment and directly affects job satisfaction. Nurses with superior health levels tend to exhibit higher work energy and emotional wellbeing, which facilitates more active engagement in their professional duties. This heightened engagement fosters greater responsibility and loyalty to their organization, resulting in higher levels of job embeddedness. In contrast, health-related challenges may hinder nurses’ ability to perform their job responsibilities, cultivate negative attitudes towards their work, and diminish overall job satisfaction, thereby decreasing the degree of job embeddedness. In terms of educational level, nurses with bachelor’s degrees exhibited relatively low job embeddedness, with mean scores of 23.41 ± 6.01. In China, the employment type of nurses with bachelor’s degree is mostly contractual in recent years. Because nurses with junior college degrees have relatively low expectations of their jobs, given their qualifications, knowledge, skill levels, etc., their self-worth can be more readily reflected in their sense of satisfaction with the organization, facilitating job embeddedness in the organization. Compared with nurses with graduate degrees, undergraduate nurses are at a disadvantage in terms of career development and welfare benefits.

The results showed that job resources were positively associated with job embeddedness with coefficient value of 0.214. At the same time, job resources were significantly negatively associated with WFC (*β* = −0.495) and significantly positively associated with WFE (*β* = 0.395), which is similar to previous research findings (29, 56). According to the Conservation of Resources Theory (57), individuals inherently strive to conserve, protect, and acquire resources. Both the threat of resource loss and the actual loss can lead to tension and stress. Sufficient job resources can fulfill the need for resource acquisition and mitigate the depletion of time, psychological resources, and other factors resulting from work-related pressures. This process promotes work–family balance and positively impacts individuals. Nurses receive salary compensation, benefits, and allowances throughout their work, which can provide financial support to them and their families. At the same time, work rewards and participation in decision making will encourage self-respect and a harmonious atmosphere in the organization. Improvements in levels of work skills can help nurses meet the demands of their position and enhance their own sense of self-worth. This will give nurses a stronger attachment and sense of belonging to their organization, so that they will show more enthusiasm for their work, and the various connections formed in the organization will be closer, meaning a higher the level of job embeddedness (58). At the same time, these job resources largely alleviate WFC and effectively enhance WFE. Hobfoll (57) and Lin et al. (59) propose that job resources play a crucial role in facilitating the transfer of energy between work and family domains. This process enables nurses to achieve a more effective balance between their professional and familial responsibilities, both in terms of time management and emotional behavior, thereby enhancing their capacity to fulfill their roles in both spheres.

The results of the study showed that WFC and WFE were significantly associated with job embeddedness (*β* = −0.243, 0.111). The coefficient values for the indirect paths of WFC and WFE were 0.157 and 0.069, respectively. According to the theory of work–family boundary management (24), WFC can lead to confusion in the boundary between an individual’s work and family spheres of transition, which in turn can have negative impacts. For employees who experience WFC, it is difficult to make a smooth role transition between the boundaries of the work and family life domains, producing negative results. Tertiary public hospitals combine high-level medical, teaching, and research tasks. Nurses work longer hours, are under greater pressure, and have more complex interpersonal relationships and higher emotional labor demands than those in other hospitals (60). Work, as a necessary means of survival, can lead to nurses investing their time and energy primarily in their work, limiting their ability to realize their family responsibilities and resulting in work intruding on family life (61). Moreover, because most nurses are female, they bear heavier family responsibilities in raising children and supporting the older adult, under the influence of traditional Chinese culture (20). To fulfill their family responsibilities, nurses may encounter conflicts with their work. Nurses’ WFC may make it difficult for them to maintain a high level of physical and mental engagement with nursing, and the degree of closeness to the organization may decrease, leading to negative behaviors at work and a decrease in job embeddedness.

By contrast, WFE effectively enhances nurses’ job embeddedness. For example, when an individual is under greater pressure, frustrated, or confused at work, the support provided by their family can enable them to fulfill their work duties and complete work tasks (62, 63). Moreover, nurses can give financial support to their families through their work. Continuous improvements in interpersonal communication skills at work will also play a contributing role in nurses’ relationships with their families, and rich and professional medical knowledge can provide health guidance to families. All these factors will enhance nurses’ confidence in coping with work difficulties, increase their satisfaction and dependence on nursing and their organizations, leading to a closer connection between nurses and their organizations and enhancing their job embeddedness (64).

Five dimensions of job resources were analyzed by establishing an association with job embeddedness. The study results showed that the dimensions of social support and skill diversity had the greatest impact on job embeddedness of the five dimensions (*β* = 0.288, *β* = 0.374). Social support refers to how much a person receives help, guidance, comfort, and information from social relationships (65, 66). Cohen and Wills advanced the direct effects hypothesis (67), positing that social support functions as a coping resource that consistently benefits an individual’s physical and mental health, regardless of stress levels. Nurses’ work environments are often stressful, including emotional stress, workload, and job complexity (68). Social support can give individuals an emotional connection that makes them feel respected and valued (69). It can act as a buffer against stress and mitigate the negative effects of job stress, thus increasing the strength and closeness of the individual’s connection to the job. Skill diversity refers to the richness of the types of skills that employees use and develop on the job (70). Individuals who master various skills are better able to adapt to organizational changes and variations in job requirements, which can satisfy the intrinsic motivation of individuals and contribute to their work engagement and job satisfaction (71), thus enhancing the degree of job embeddedness of individuals (18).

Social support and skill diversity have the greatest impact on nurses’ job embeddedness mainly because they fulfill nurses’ emotional and social needs and personal growth and development needs, respectively. As practitioners in the caring professions, nurses need emotional support to cope with the stresses of their work and professional support for personal growth and skill enhancement. The nature of nursing requires a high degree of teamwork and continuous skill renewal, and social support and skill diversity aptly meet these needs.

Job control refers to an employee’s perceived ability to exert some control over the work environment (72). Theoretically, higher job control may allow employees a higher degree of autonomy to adjust their work to suit their personal needs and preferences (73), which may positively affect their job embeddedness. However, this study found that job control did not have a significant effect on nurses’ job embeddedness (*p* > 0.05). Possible reasons for this include the fact that nurses’ work environments and healthcare processes are often subject to strict regulations and limitations and that stringent rules and regulations and quick responses in emergencies may limit their feelings of job control (74, 75), which in turn may limit the effect of job control on job embeddedness. Second, while job control grants individual autonomy, it may be accompanied by increased responsibility and stress. For nurses, this stress may counteract the positive effects of job control.

The results of this study indicate that decision-making participation has a direct association with job embeddedness (*β* = 0.175, *p* < 0.05). When employees can participate in decision-making, they are more likely to identify with their job roles and the organization’s values, which can increase career satisfaction and commitment to the job (76, 77), which in turn directly impacts job embeddedness. Second, participation in decision-making indicates that management recognizes employees’ abilities and contributions, and this recognition can enhance employees’ self-confidence and wellbeing (78) and promote their embeddedness in the workplace.

In addition, decision-making participation indirectly affects job embeddedness through work–family enablement (*β* = 0.039). This resource may promote positive interactions between employees’ work and family roles. When nurses can participate in decision-making, it may make them feel more fulfilled at work, and this positive work experience may spill over into family life. However, decision-making participation cannot indirectly affect job embeddedness through WFC (*p* > 0.05). The reason for this may lie in the inherent high intensity, irregular working hours, and urgent tasks of nursing, which are job characteristics that may lead to WFC (79). Even if nurses are involved in decision-making, these basic job demands remain challenging to change and may not resolve these deep-rooted problems. Furthermore, some nurses may feel overburdened by the additional time and effort required by participation in professional decision-making processes (80), or their engagement may be constrained by familial responsibilities (81). Therefore, decision-making participation will not enhance job embeddedness by reducing conflict.

According to the Conservation of Resources Theory, individuals actively cultivate resources they perceive as beneficial for personal growth and development. These resources are typically categorized into four types: material resources, conditional resources, personality traits, and energy resources (57, 82). Conditional resources, specifically, are potential resources that facilitate the acquisition of essential resources. In this study, skill variety, social support, job control, and decision participation are identified as conditional resources. Nonetheless, a comparative analysis reveals significant differences among these variables. Firstly, in terms of focus, skill variety and social support primarily address individual-level factors, being intricately linked to personal development elements such as economic income, work efficiency, career advancement, and emotional support. Conversely, job control and decision participation pertain more to organizational-level factors, emphasizing the provision of autonomy and decision-making opportunities within the workplace. Secondly, concerning the mode of influence, skill variety and social support exert a direct impact, typically influencing individuals through improvements at the personal level and the establishment of support networks. In contrast, job control and decision participation have an indirect influence, as they enhance individuals’ voice and involvement within the organization, thereby improving organizational efficiency and fairness, which subsequently produces indirect benefits for individuals.

Thirdly, with regard to the origins of resources, skill variety and social support primarily arise from individuals’ personal learning, growth, and social interactions. Continuous learning, experience accumulation, and the expansion of interpersonal networks are essential for enhancing skill variety anding obtain social support. In contrast, job control and participation in decision-making are primarily derived from organizational management and institutional frameworks. Organizations must establish appropriate systems and policies to afford individuals greater job control and opportunities for decision-making. The findings of this study suggest that, compared to organizationally-related conditional resources, skill variety and social support exert a more significant influence on job embeddedness among nurses in public hospitals. These resources directly influence their daily work experiences and career development. This underscores that social support and skill variety more effectively address nurses’ professional needs and contribute to their mental and physical wellbeing.

The study results show that rewards do not directly affect job embeddedness (*p* > 0.05). According to self-determination theory, an individual’s intrinsic motivation is the key driver of behavior (83). Rewards can be extrinsic or intrinsic (84). The connotation of reward in this study focuses more on it as an extrinsic motivation (38), which tends to be immediate feedback for specific behaviors. Job embedding, on the other hand, is a long-term, cumulative process. While rewards may provide short-term motivation, they may not be sufficient to maintain employees’ sense of job embeddedness over the long term. Rewards are not indirectly embedded in work through work–family enhancement. Rewards are associated with job performance and may have positive effects within the workplace that do not easily translate into resources in the family sphere. Thus, rewards do not enhance positive work–family interactions. Rewards act only indirectly on job embeddedness through WFC. High rewards can act as a stress-relieving mechanism to reduce work stress, increase job satisfaction, and reduce work-related dissatisfaction, thus reducing WFC to some extent.

The theoretical and practical implications of this study are noteworthy. From a theoretical perspective, the study is grounded in the JD-R model, which explains the effects of job stressors and resources on employees’ psychological and physiological wellbeing. By examining the relationship between job resources and job embeddedness among nurses in Chinese public hospitals, this research extends the applicability of the JD-R model, thereby contributing to its validation and refinement within specific occupational contexts. Secondly, job embeddedness provides a novel perspective on the relationship between employees and organizations and remains in the exploratory stage. By focusing on nurses as a distinct occupational group, this study seeks to expand the research boundaries of job embeddedness and enhance the comprehension of its fundamental concepts. Thirdly, numerous contemporary studies investigate the relationship between specific dimensions of job resources and job embeddedness, WFC, and WFE. This study not only corroborates the influence of job resources on job embeddedness and examines the roles of WFC and WFE in this context but also refines the analysis of specific job resource dimensions. Such an in-depth analysis highlights the relative significance of various job resources, contributing to the development of a more comprehensive theoretical framework and deepening the understanding of the mechanisms through which job embeddedness functions. This, in turn, offers valuable insights into the diverse behaviors and attitudes of employees.

The practical significance of this study is twofold. Firstly, by examining the relationship between nurses’ job resources and job embeddedness, and identifying the specific dimensions of job resources that most significantly influence job embeddedness, the findings offer a theoretical foundation for hospital human resource management. This knowledge aids hospital administrators in key factors that enhance nurses’ job embeddedness, thereby enabling more effective resource allocation and strengthening support systems. These measures play a significant role in enhancing job embeddedness, which directly increases employee retention rates, reduces turnover intentions among nurses, and alleviates the pressures faced by human resource management. Secondly, given that nurses are integral members of medical service teams, their levels of job embeddedness are directly linked to the quality and efficiency of healthcare services. This research provides hospital managers with theoretical insights into optimizing the factors influencing job embeddedness, which can enhance employee wellbeing and improve the overall quality of nursing services.

## 6 Conclusion

The study results show that job resources were associated with nurses’ job embeddedness directly and indirectly. Regarding the specific dimensions of job resources, social support, and skill diversity had the greatest impact on job embeddedness. The results suggest that nursing managers should emphasize the two dimensions of social support and skill diversity to improve nurses’ job embeddedness through measures such as establishing supportive interpersonal networks and providing diverse job content and learning opportunities. To enhance social support within healthcare settings, hospitals should implement regular team-building activities designed to promote effective communication and collaboration among staff, thereby cultivating a supportive and trustworthy work environment. Additionally, mental health counseling services should be established, including anonymous psychological counseling hotlines or online platforms, alongside regular mental health lectures and workshops, to aid nurses in managing stress and preventing professional burnout. For newly recruited nurses, a mentoring system should be introduced, pairing each new nurse with an experienced mentor to provide professional guidance, psychological support, and resource sharing, thus fostering a sense of belonging. Concurrently, hospitals should offer a flexible scheduling system that permits nurses to adjust their shifts in accordance with their familial obligations. To enhance skill diversity among nursing staff, hospitals can establish educational funds to facilitate nurses’ participation in external training sessions, seminars, and advanced courses. Concurrently, hospitals should implement comprehensive internal training and development programs to augment both clinical and non-clinical competencies. Additionally, hospitals can introduce innovation incentive mechanisms to motivate nurses to propose creative ideas and solutions aimed at improving nursing services. Regularly organizing innovation competitions and brainstorming sessions can further stimulate creativity. Moreover, implementing a job rotation system can provide nurses who are interested in rotating with opportunities to work across different departments and fields, thereby engaging in a variety of tasks and accumulating a breadth of experiences.

This study has several limitations. Firstly, the sample was obtained from five public hospitals in Weifang City. The selection of this specific geographical location and the hospital level may influence the comprehensiveness of the data. Consequently, the findings may not fully reflect the general conditions in broader regions or other areas. To validate the results, future studies should conduct more extensive and diverse surveys. Secondly, data collection relied on a questionnaire survey, which may be influenced by recall bias on the part of the respondents. Given that cross-sectional studies typically require data to be collected at a single point in time, participants may need to recall past events or behaviors, potentially leading to inaccuracies in the information provided. Thirdly, the inherent design characteristics of cross-sectional studies preclude the establishment of causal relationships between variables. Given that data is collected concurrently, these studies can only identify associations among various factors. Consequently, establishing causality typically requires corroboration from alternative study designs, such as longitudinal studies. Fourthly, although statistical techniques can account for some known confounding variables, unrecognized or unmeasured confounding factors may still exist, potentially affecting the accuracy of the study’s findings. Moreover, beyond work resources, job embeddedness may be affected by a range of factors. Future research should explore additional determinants related to nurses’ work resources, thereby contributing to the theoretical foundation for strengthening the attachment between nurses and their organizations.

## Data Availability

The original contributions presented in the study are included in the article/Supplementary material, further inquiries can be directed to the corresponding author.
