# Supplementary material for: Relationship between job resources and job embeddedness among tertiary-level public hospital nurses: parallel mediating roles of work–family conflict and work–family enrichment
Source: Front Public Health. 2025 Jun 2;13:1527511. doi: 10.3389/fpubh.2025.1527511 (PMC12171369; doi:10.3389/fpubh.2025.1527511)
Supplement: Supplementary file 1 [file Data_Sheet_1.pdf]

Number	Sex (1=Male;2= Female)	Age (1=≤ 30;2=31- 40;3=41- [illegible]	Educational _level (1=Junior [illegible]	Employmen t_type (1=Permane [illegible]	Years_of_se rvice (1=≤ 5;2=6- [illegible]	Healthy_stat us (1=Very unhealthy;2 [illegible]	Work_inten sity (1=Very low/rarely;2 [illegible]	JR1 (1=Never;2 =Seldom;3= [illegible]
1	2	3	2	2	2	4	2	5
2	2	2	2	2	1	2	3	4
3	2	3	2	2	3	4	3	1
4	2	3	2	2	3	4	3	4
5	2	5	2	1	5	4	4	5
6	2	4	2	1	5	3	4	3
7	2	3	2	2	3	3	2	5
8	2	5	2	1	5	4	5	4
9	2	3	1	2	3	3	3	4
10	2	5	2	1	5	3	3	5
11	2	3	2	2	2	3	2	4
12	2	2	2	2	2	3	2	5
13	2	4	2	1	5	3	3	4
14	2	2	2	2	1	2	3	5
15	2	2	2	2	1	2	2	4
16	2	3	2	2	3	3	3	5
17	2	4	2	1	5	3	4	4
18	2	2	1	2	1	2	3	5
19	2	2	2	2	1	2	3	5
20	2	2	1	2	1	2	3	5
21	2	4	2	1	5	4	4	5
22	2	4	2	1	5	4	3	4
23	2	3	2	2	3	2	3	3
24	2	2	2	2	2	3	4	5
25	2	4	2	1	5	3	2	4
26	2	4	2	1	5	4	3	5
27	2	3	2	2	2	3	3	5
28	2	3	2	2	2	4	3	5
29	2	5	2	1	5	2	4	4
30	2	2	2	2	1	3	2	5
31	2	2	2	2	1	3	3	4
32	2	3	2	2	4	2	3	5
33	2	3	2	2	2	2	2	4
34	2	2	2	2	1	4	2	3
35	2	4	2	1	5	4	3	5
36	2	2	2	2	2	2	4	5
37	2	4	2	1	5	3	2	5
38	2	2	2	2	2	3	3	5
39	2	4	2	1	5	3	2	3
40	2	2	2	2	1	3	3	4
41	2	4	2	1	5	3	5	4
42	2	4	2	1	5	3	3	4
43	2	3	2	2	2	3	3	5
44	2	4	2	1	5	4	2	5
45	2	5	2	1	5	3	3	4
46	2	4	2	1	5	3	3	4
47	2	4	2	1	5	3	4	4
48	2	2	2	2	1	2	4	5
49	2	3	2	2	2	3	3	5
50	2	3	2	2	2	3	4	5
51	2	2	2	2	2	4	2	5

52	2	2	3	2	1	3	3	5
53	2	3	2	2	2	2	2	5
54	2	3	2	2	2	3	2	5
55	2	2	2	2	1	3	2	3
56	2	3	2	2	4	3	2	4
57	2	2	2	2	1	3	4	5
58	1	2	2	2	1	4	2	3
59	2	4	2	1	5	3	3	5
60	2	5	2	1	5	2	3	4
61	2	2	2	2	2	4	1	4
62	2	2	2	2	2	3	2	5
63	2	2	2	2	1	3	3	3
64	2	2	2	2	1	3	3	5
65	2	2	2	2	1	3	2	4
66	2	3	2	2	3	3	3	4
67	2	2	2	2	1	3	2	5
68	2	2	2	2	2	2	3	5
69	2	3	2	2	3	4	2	5
70	2	3	2	2	2	3	2	4
71	2	2	2	2	2	3	4	4
72	2	3	2	2	3	3	2	5
73	2	3	2	2	2	4	2	4
74	2	3	2	2	3	3	3	5
75	2	4	2	2	4	3	1	3
76	2	2	2	2	1	4	2	3
77	2	4	2	1	5	2	2	1
78	2	2	2	2	2	3	3	5
79	2	2	2	2	2	3	3	4
80	2	3	2	2	3	3	2	4
81	2	3	2	2	2	3	4	5
82	2	3	2	2	3	3	2	4
83	2	2	2	2	1	3	4	5
84	2	3	2	2	3	3	3	3
85	2	2	2	2	1	2	2	5
86	2	2	2	2	2	2	2	4
87	2	2	2	2	1	2	4	5
88	2	3	2	2	2	3	4	4
89	2	4	2	1	5	3	3	3
90	2	3	2	2	4	2	4	3
91	2	3	2	2	3	3	3	3
92	2	2	2	2	1	2	4	5
93	2	3	2	2	3	4	4	3
94	2	2	2	2	1	1	4	5
95	2	2	2	2	1	2	3	5
96	2	2	2	2	1	3	2	3
97	2	2	2	2	1	4	2	3
98	2	3	2	2	1	3	4	5
99	2	2	2	2	1	3	4	5
100	2	2	2	2	2	3	4	4
101	2	3	2	2	2	3	4	5
102	2	3	2	2	2	3	3	5
103	2	3	2	2	2	4	2	2
104	2	3	2	2	2	4	2	3
105	2	3	1	2	1	3	3	5
106	2	3	2	2	3	3	3	3

107	2	2	2	2	1	3	2	3
108	2	2	2	2	1	3	3	3
109	2	2	2	2	1	2	3	5
110	2	3	2	2	2	4	3	5
111	2	4	2	1	5	4	2	5
112	2	2	2	2	1	3	2	5
113	2	2	1	2	1	3	4	5
114	2	3	2	2	2	4	2	3
115	2	3	2	2	2	1	2	4
116	2	2	2	2	1	4	1	4
117	2	2	2	2	1	4	2	4
118	2	2	2	2	2	3	4	5
119	2	3	2	2	2	3	4	5
120	2	3	2	2	3	3	4	3
121	2	3	2	2	4	3	3	5
122	2	3	2	2	3	3	2	3
123	2	5	2	1	5	3	4	4
124	2	3	2	2	3	3	4	4
125	2	3	2	2	2	3	2	3
126	2	2	2	2	2	3	4	4
127	2	3	2	2	3	3	4	4
128	2	3	2	2	1	3	3	5
129	2	4	2	1	5	3	3	1
130	2	3	2	2	2	4	3	4
131	2	2	1	2	1	3	2	4
132	2	3	2	2	2	3	3	5
133	2	3	2	2	2	3	3	5
134	2	2	2	2	1	4	2	5
135	2	3	2	2	2	3	3	4
136	2	3	2	2	2	3	4	5
137	2	2	2	2	1	1	4	3
138	2	5	2	1	5	3	3	2
139	2	3	2	2	2	3	2	4
140	2	3	2	2	2	4	1	4
141	2	2	2	2	1	3	3	4
142	2	2	2	2	1	4	2	3
143	2	3	2	2	2	3	2	5
144	2	3	2	2	2	3	2	4
145	2	3	2	2	2	4	3	3
146	2	2	2	2	1	2	3	4
147	2	2	2	2	2	4	3	5
148	2	5	2	1	5	3	4	4
149	2	3	3	1	4	3	4	5
150	2	3	2	2	3	2	4	5
151	2	3	2	2	3	3	4	4
152	2	3	2	2	3	4	2	3
153	2	3	2	2	2	2	3	4
154	2	2	2	2	2	3	3	4
155	2	2	2	2	2	4	3	4
156	2	2	2	2	1	2	5	5
157	2	3	2	2	2	2	5	5
158	2	3	2	2	4	2	5	5
159	2	3	2	2	2	3	3	4
160	2	3	2	2	2	3	2	5
161	1	2	2	2	1	2	3	3

162	2	2	2	2	2	3	2	3
163	2	3	2	2	3	3	3	4
164	2	3	2	2	4	3	3	4
165	2	3	2	2	2	3	4	4
166	2	2	2	2	1	1	4	5
167	1	2	2	2	1	2	4	5
168	2	3	2	2	3	3	3	3
169	2	2	2	2	1	2	4	5
170	2	3	2	2	2	3	2	4
171	2	2	2	2	1	3	4	5
172	2	2	2	2	1	3	4	5
173	1	2	2	2	2	3	4	5
174	2	3	2	2	2	3	4	4
175	2	3	2	2	2	3	2	4
176	2	4	2	2	5	3	2	5
177	2	2	2	2	1	3	3	4
178	2	3	2	2	2	3	2	4
179	2	2	2	2	2	3	2	4
180	1	2	2	1	2	4	1	5
181	2	3	1	2	2	3	3	4
182	2	2	2	2	1	3	5	4
183	2	2	2	2	1	3	4	4
184	2	2	2	2	2	3	4	5
185	2	2	2	2	2	3	4	5
186	2	3	2	2	2	2	3	4
187	2	3	2	2	3	2	2	5
188	2	3	1	2	3	2	3	3
189	2	2	2	2	1	3	5	5
190	2	2	2	2	1	2	3	5
191	2	2	2	2	1	3	4	4
192	2	2	2	2	2	3	3	4
193	2	3	2	2	3	3	4	5
194	2	3	2	2	3	3	4	5
195	2	3	2	2	2	3	3	3
196	2	3	2	2	2	2	2	5
197	2	4	2	1	5	3	4	5
198	2	3	2	2	2	4	3	4
199	2	3	2	2	3	2	4	5
200	2	3	2	2	4	3	3	5
201	2	4	2	1	5	3	4	3
202	2	2	1	2	1	2	5	5
203	2	3	2	2	2	2	2	5
204	2	2	2	2	1	2	2	4
205	2	3	2	2	2	3	5	5
206	2	4	2	1	5	2	4	4
207	2	3	2	2	3	3	2	5
208	2	3	2	2	3	3	3	3
209	2	2	2	2	1	2	4	4
210	2	3	2	2	3	3	4	4
211	2	3	2	2	2	2	4	4
212	2	2	2	2	1	3	2	4
213	2	3	2	2	2	3	3	3
214	2	3	2	2	2	3	3	4
215	2	3	2	2	3	2	3	4
216	2	4	2	2	5	2	3	4

217	2	3	2	2	2	2	2	4
218	2	2	2	2	2	2	4	5
219	2	3	2	2	3	4	2	4
220	2	3	2	2	2	3	2	4
221	2	3	2	2	4	3	2	3
222	2	2	3	2	1	3	4	5
223	2	2	2	1	2	4	1	2
224	2	2	3	1	3	4	5	4
225	2	3	3	2	4	3	3	4
226	2	2	1	2	2	2	3	5
227	2	3	2	2	3	2	4	4
228	2	2	2	2	1	4	3	5
229	2	4	2	1	4	3	3	4
230	2	2	2	2	1	3	3	4
231	2	3	2	2	2	3	3	5
232	2	2	2	2	1	2	3	3
233	2	4	2	1	5	3	4	4
234	2	4	2	1	5	4	1	5
235	2	3	2	1	4	3	3	4
236	2	3	2	2	3	4	5	3
237	2	2	2	2	1	2	3	5
238	2	4	3	2	4	4	4	5
239	2	4	2	2	4	4	4	4
240	2	3	2	2	2	2	5	1
241	2	3	2	2	2	3	3	4
242	2	3	2	2	4	3	2	4
243	2	3	2	2	3	3	3	5
244	2	2	2	2	1	1	1	5
245	2	3	2	2	2	3	3	4
246	2	3	2	2	2	2	3	3
247	2	2	2	2	2	2	4	5
248	2	3	2	2	3	3	3	4
249	2	3	2	2	2	3	2	4
250	2	3	1	2	2	2	4	4
251	2	3	2	2	2	3	2	4
252	1	2	2	2	5	2	5	5
253	2	2	2	2	2	3	2	4
254	2	3	2	2	4	3	2	4
255	2	2	2	2	1	3	4	5
256	2	2	2	2	1	2	4	5
257	2	3	2	2	2	3	3	5
258	2	3	2	2	3	3	4	4
259	2	2	2	2	1	3	4	5
260	2	5	2	1	5	3	3	4
261	1	2	1	2	1	2	4	5
262	2	3	2	2	3	3	4	4
263	2	3	2	2	1	3	3	4
264	2	3	2	2	2	3	2	4
265	2	4	2	1	5	3	2	3
266	2	3	2	2	2	3	4	5
267	2	4	2	1	5	3	4	4
268	2	3	2	2	3	4	3	5
269	2	3	3	1	2	2	4	5
270	2	3	2	2	3	2	4	4
271	2	2	2	2	1	3	4	4

272	2	3	2	2	3	3	3	5
273	2	3	2	2	3	3	5	3
274	2	3	2	2	2	2	3	4
275	2	3	2	1	5	2	4	5
276	2	2	2	2	1	2	4	5
277	2	3	2	2	2	2	3	5
278	2	5	2	1	5	3	4	3
279	2	4	2	1	5	3	4	4
280	2	3	2	2	3	3	4	5
281	2	3	2	2	1	3	2	3
282	2	2	2	2	1	2	3	5
283	2	2	1	2	1	2	4	4
284	2	3	2	1	4	3	3	5
285	2	4	2	1	5	3	2	3
286	2	3	2	2	3	2	3	5
287	2	2	2	2	1	2	2	3
288	2	2	1	2	2	3	4	5
289	2	2	1	2	2	3	3	1
290	2	2	2	2	1	3	3	3
291	2	3	2	2	2	3	3	5
292	2	2	2	2	1	2	4	4
293	2	3	2	2	3	3	2	4
294	2	2	2	2	1	3	3	5
295	2	3	2	2	3	2	4	5
296	2	4	2	2	5	2	3	2
297	2	2	2	2	1	3	4	4
298	2	4	2	1	5	3	3	4
299	2	2	2	2	2	2	3	5
300	2	3	2	2	3	3	3	4
301	2	3	2	2	4	3	2	4
302	2	2	2	2	2	3	3	5
303	2	3	2	2	2	2	3	5
304	2	3	2	2	2	1	5	4
305	2	3	2	2	3	4	1	5
306	2	2	2	2	2	3	3	4
307	2	4	2	1	5	2	4	4
308	2	4	2	1	5	3	4	4
309	2	2	1	2	1	3	3	4
310	2	2	2	2	1	3	4	5
311	2	2	2	2	1	3	4	5
312	2	2	2	2	2	2	4	5
313	2	2	2	2	1	2	4	4
314	2	3	2	2	3	3	2	4
315	2	2	2	2	1	2	4	4
316	2	2	2	2	2	3	4	5
317	2	3	2	2	4	3	2	5
318	2	3	2	2	2	4	2	4
319	2	2	2	2	3	1	4	4
320	2	3	2	2	2	2	3	5
321	2	2	2	2	2	2	3	5
322	2	2	2	2	1	2	4	5
323	2	2	2	2	2	4	2	3
324	2	3	2	2	2	4	2	5
325	2	3	2	2	3	2	4	5
326	2	3	2	2	3	3	4	4

327	2	3	2	2	3	3	3	4
328	2	2	2	2	1	4	2	4
329	2	4	2	1	5	3	4	3
330	2	3	2	2	3	2	4	5
331	2	2	2	2	2	3	4	5
332	2	3	2	2	4	3	3	4
333	2	2	2	2	1	2	5	5
334	2	2	2	2	1	2	3	5
335	2	2	2	2	1	3	4	4
336	1	2	2	2	1	2	2	5
337	2	3	2	2	2	3	2	4
338	2	3	2	2	3	3	4	4
339	2	3	2	2	4	3	3	4
340	2	3	2	2	2	3	4	4
341	2	4	2	1	5	4	5	5
342	2	3	2	2	2	3	2	4
343	2	2	2	2	1	2	4	5
344	2	3	2	2	3	3	4	4
345	2	2	2	2	2	2	4	4
346	2	3	2	2	2	2	4	4
347	2	2	2	2	1	2	2	5
348	2	2	1	2	1	2	4	5
349	2	2	2	2	2	1	5	5
350	2	3	2	2	3	3	2	5
351	2	3	2	2	3	3	2	2
352	2	3	2	2	2	3	4	4
353	2	3	2	2	2	4	4	4
354	2	2	2	2	2	3	2	5
355	2	3	2	2	2	4	2	4
356	2	2	2	2	2	3	4	5
357	2	2	2	2	2	2	5	5
358	2	2	2	2	1	2	4	5
359	2	2	2	2	1	2	4	4
360	2	2	1	2	1	3	3	4
361	2	3	2	2	2	2	3	5
362	1	2	1	2	1	2	3	4
363	1	2	1	2	1	1	5	5
364	2	3	2	2	2	3	3	5
365	2	5	2	1	5	3	3	4
366	2	2	2	2	2	4	3	5
367	2	3	2	2	3	4	4	5
368	2	3	2	2	2	2	3	5
369	2	4	2	1	5	3	4	4
370	2	2	2	2	1	3	4	5
371	2	2	2	2	1	4	2	5
372	2	4	2	1	5	3	2	4
373	2	3	2	2	2	2	3	4
374	2	3	2	2	2	3	2	4
375	2	3	2	2	2	3	2	4
376	2	3	2	2	2	3	3	5
377	2	3	2	1	4	2	4	4
378	2	3	2	2	2	2	4	5
379	2	3	2	2	3	4	2	5
380	2	3	2	2	4	3	3	5
381	2	2	2	2	1	3	3	5

382	2	3	2	2	2	3	4	4
383	2	3	2	2	3	2	3	5
384	2	3	2	2	2	2	2	4
385	2	3	2	2	3	2	3	4
386	2	2	2	2	1	3	2	4
387	2	2	2	2	2	3	3	2
388	2	3	2	2	2	2	4	3
389	2	2	2	2	2	4	3	4
390	2	5	2	1	5	3	3	4
391	2	3	2	2	2	3	2	4
392	2	2	2	2	2	2	2	4
393	2	3	2	2	3	4	2	4
394	2	3	2	2	3	3	2	1
395	2	3	2	2	3	1	4	5
396	2	2	2	2	2	2	3	5
397	2	3	2	2	2	2	1	3
398	2	3	2	2	3	1	4	4
399	2	3	2	2	2	4	1	3
400	2	3	2	2	2	4	3	4
401	2	4	2	1	4	3	4	4
402	2	3	2	2	2	2	4	5
403	2	3	2	2	3	3	4	5
404	2	2	2	2	2	2	4	4
405	2	2	2	2	1	3	4	5
406	2	2	2	2	1	2	5	5
407	2	2	2	2	2	4	3	5
408	2	2	2	2	2	3	3	5
409	2	3	2	2	2	4	3	5
410	2	3	2	2	3	2	4	3
411	2	3	2	2	3	3	3	5
412	2	2	2	2	1	3	2	3
413	2	3	1	2	2	3	3	3
414	2	2	2	2	2	3	2	4
415	2	3	2	1	4	4	3	5
416	2	2	2	2	1	2	4	5
417	2	3	2	2	2	3	3	5
418	2	3	2	2	2	3	3	5
419	2	2	2	2	1	3	4	5
420	2	2	2	2	1	3	4	5
421	2	3	2	2	4	3	3	5
422	2	2	1	2	1	4	2	5
423	2	4	2	2	4	3	2	3
424	2	2	2	2	1	2	2	3
425	2	2	2	2	1	3	3	5
426	2	3	2	2	1	3	3	5
427	2	3	3	1	2	2	3	4
428	2	2	1	2	2	3	2	5
429	2	2	1	2	1	2	2	5
430	2	3	2	2	2	2	2	3
431	2	3	2	2	2	2	4	3
432	2	2	1	2	1	2	4	4
433	2	3	2	2	3	2	2	1
434	2	2	2	2	1	2	5	5
435	2	4	2	1	5	4	4	3
436	2	2	2	2	2	3	3	4

437	2	2	2	2	1	4	2	3
438	2	2	2	2	2	1	3	5
439	2	3	2	2	2	3	3	4
440	2	3	2	2	4	3	2	4
441	2	2	1	2	1	2	2	4
442	2	3	2	2	3	4	2	5
443	2	3	2	2	3	2	4	4
444	2	2	2	2	1	2	3	5
445	2	3	2	2	2	3	3	4
446	2	3	2	2	2	3	2	3
447	2	2	2	2	2	2	4	5
448	2	3	2	1	4	2	2	5
449	2	3	2	2	2	3	3	4
450	2	4	2	2	4	2	4	5
451	2	3	1	2	2	2	3	5
452	2	2	2	2	1	2	5	5
453	2	4	2	1	5	3	4	4
454	2	4	2	1	5	3	3	4
455	2	2	2	2	2	2	3	5
456	2	3	2	2	2	3	2	5
457	2	3	2	2	3	3	4	5
458	2	3	2	2	3	1	4	5
459	2	2	2	2	2	3	3	5
460	2	4	2	1	5	3	2	4
461	1	3	2	2	2	4	2	5
462	2	2	2	2	1	3	4	4
463	2	3	2	2	3	2	3	4
464	2	2	2	2	2	3	4	5
465	2	2	2	2	1	2	4	4
466	2	3	2	2	2	2	3	4
467	2	2	2	2	2	3	3	5
468	2	3	2	2	2	3	2	4
469	2	4	2	2	5	2	3	4
470	2	3	2	2	2	3	3	3
471	2	3	2	2	3	4	3	4
472	2	3	2	2	3	3	3	4
473	2	5	2	1	5	3	4	4
474	2	5	2	2	1	2	3	5
475	2	2	2	2	2	2	4	5
476	2	2	2	2	2	3	3	5
477	1	2	1	2	1	3	4	5
478	2	2	2	2	2	2	3	4
479	2	2	1	2	1	3	5	5
480	2	5	2	1	5	2	3	3
481	2	3	2	2	3	3	3	3
482	2	2	3	2	1	2	4	4
483	2	2	2	2	2	3	2	5
484	2	2	2	1	1	4	4	3
485	2	4	2	1	5	3	3	4
486	2	3	1	2	2	3	3	5
487	2	2	2	2	1	3	4	3
488	2	2	2	2	2	3	2	3
489	2	2	1	2	2	2	5	5
490	2	2	2	2	1	3	2	4
491	2	2	2	2	2	3	3	5

492	2	2	2	2	1	2	4	5
493	2	3	2	2	3	4	3	4
494	2	4	2	1	4	2	3	3
495	2	2	2	2	1	3	5	5
496	2	3	2	2	4	4	2	4
497	2	3	2	2	3	3	4	5
498	2	3	2	2	3	3	4	4
499	2	2	2	2	1	4	2	3
500	2	2	2	2	1	3	4	5
501	2	3	2	2	4	2	4	4
502	1	2	1	2	1	3	4	5
503	2	3	2	2	3	3	2	3
504	2	3	3	2	3	3	2	4
505	2	4	2	1	5	4	2	4
506	2	2	2	2	1	3	4	5
507	2	2	2	2	1	2	3	5
508	2	2	2	2	1	2	5	3
509	2	2	2	2	1	2	3	4
510	2	3	2	2	2	3	3	4
511	2	2	1	2	2	2	5	5
512	2	2	2	2	2	3	2	5
513	2	3	2	2	2	3	4	4
514	2	3	2	2	2	3	2	3
515	2	2	2	2	2	2	4	5
516	2	2	1	2	2	3	4	4
517	2	3	2	2	2	4	2	4
518	2	3	2	2	2	2	4	5
519	2	2	2	2	2	3	3	5
520	1	2	1	2	1	3	3	4
521	2	3	2	2	2	3	2	3
522	2	2	1	2	1	3	4	5
523	2	2	2	2	2	2	3	4
524	2	4	2	2	4	4	3	4
525	2	3	2	2	3	3	3	4
526	2	2	2	2	1	3	4	5
527	2	2	2	2	1	3	4	5
528	2	2	2	2	1	3	4	5
529	2	3	2	2	3	2	2	4
530	2	3	2	2	3	3	4	5
531	2	3	2	2	3	3	4	5
532	2	2	2	1	1	2	4	4
533	2	2	2	2	1	2	2	5
534	2	4	2	1	4	3	3	3
535	1	2	1	2	1	2	3	5
536	2	2	2	2	2	3	3	5
537	2	2	2	2	1	3	4	5
538	2	3	2	2	4	3	2	5
539	2	3	2	2	2	3	4	4
540	2	3	2	2	3	4	2	1
541	2	2	1	2	1	2	4	4
542	2	3	2	2	2	2	3	3
543	2	3	2	2	3	3	3	5
544	2	2	2	2	2	2	3	4
545	2	3	2	2	3	3	1	4
546	1	3	2	2	2	4	3	5

547	2	2	1	2	1	3	4	5
548	2	2	2	2	1	3	4	5
549	2	2	2	2	1	4	3	5
550	2	3	2	2	3	2	3	5
551	2	3	2	2	2	3	3	5
552	2	2	2	2	2	3	3	5
553	2	3	2	2	3	3	2	4
554	2	3	2	2	3	4	3	4
555	1	2	2	2	1	2	5	5
556	2	4	2	1	1	2	4	5
557	2	4	2	2	4	2	3	4
558	2	3	2	2	3	2	3	4
559	2	3	2	2	2	2	2	4
560	2	2	2	2	1	3	3	4
561	2	2	2	2	1	3	3	5
562	2	2	2	2	1	4	3	5
563	2	3	2	1	5	3	3	4
564	2	2	2	2	1	3	4	5
565	2	3	2	2	3	3	3	4
566	2	3	2	2	2	3	3	5
567	2	2	1	2	1	2	3	3
568	2	3	2	2	4	2	3	5
569	2	3	2	2	2	3	4	5
570	2	3	2	2	2	3	3	4
571	2	3	2	2	3	3	3	3
572	2	3	2	2	2	3	4	3
573	2	2	2	1	2	2	3	5
574	1	3	2	2	3	3	3	3
575	2	4	2	1	5	3	3	5
576	2	3	2	2	4	2	3	5
577	2	4	2	1	5	4	4	4
578	2	3	2	2	3	3	4	5
579	2	3	2	2	3	3	3	4
580	2	3	2	2	3	3	3	5
581	2	4	2	1	5	4	3	4
582	2	2	2	2	2	3	2	3
583	2	4	2	1	5	2	3	4
584	2	3	2	2	2	4	1	4
585	2	2	2	2	2	2	4	4
586	2	2	2	2	1	3	3	4
587	2	2	2	2	1	3	3	3
588	2	2	2	2	1	3	3	5
589	2	2	2	2	1	3	4	5
590	2	2	2	2	2	3	4	5
591	2	3	2	2	3	2	5	5
592	2	3	2	2	2	2	4	3
593	2	3	2	2	3	3	2	4
594	2	2	2	2	2	2	3	5
595	2	2	1	2	2	3	4	5
596	2	3	2	2	3	4	2	4
597	2	3	2	2	3	2	3	5
598	2	2	2	2	2	3	2	5
599	2	3	2	2	2	3	3	5
600	2	3	1	2	1	2	4	4
601	2	3	1	2	1	2	3	5

602	2	2	2	2	1	3	3	5
603	2	4	2	1	5	3	4	5
604	2	2	2	2	1	3	1	4
605	2	2	2	2	1	3	4	5
606	2	2	1	2	2	2	4	4
607	2	3	2	1	3	4	2	4
608	2	3	2	2	2	2	2	4
609	2	3	2	2	2	1	2	4
610	2	2	1	2	1	2	4	5
611	2	2	2	2	2	2	3	5
612	2	3	2	2	2	2	4	4
613	2	3	2	2	2	2	3	4
614	2	2	1	2	1	2	3	5
615	2	3	1	2	3	3	4	5
616	2	2	2	2	1	2	4	5
617	2	3	2	2	3	3	2	4
618	2	3	2	2	2	2	2	4
619	2	2	1	2	2	1	3	4
620	2	3	2	2	4	3	2	5
621	2	3	2	2	2	3	3	4
622	2	4	2	1	5	2	3	4
623	2	3	2	2	2	2	4	4
624	2	2	2	2	1	2	4	5
625	2	2	2	2	1	2	4	5
626	2	2	2	1	1	3	3	5
627	2	3	2	2	4	2	3	4
628	2	2	1	2	1	2	3	4
629	2	3	2	2	3	2	3	5
630	2	3	2	2	4	3	2	4
631	2	3	1	2	2	3	2	4
632	2	2	1	2	1	2	4	5
633	2	2	1	2	2	4	3	5
634	2	2	1	2	1	2	3	4
635	2	2	2	2	2	3	3	4
636	2	3	2	2	4	3	2	4
637	2	2	1	2	2	3	2	5
638	2	3	1	2	1	2	4	5
639	2	2	2	2	1	2	4	5
640	2	3	2	2	2	2	3	5
641	2	4	2	2	4	2	4	4
642	2	2	1	2	1	2	4	4
643	2	3	2	2	2	3	4	5
644	2	2	2	2	1	3	4	4
645	2	3	2	2	3	2	3	3
646	2	2	2	2	1	2	3	5
647	2	3	1	2	3	2	3	5
648	2	2	2	2	1	2	4	4
649	2	2	1	2	1	2	2	5
650	2	2	2	1	5	4	1	4
651	2	3	2	2	4	3	2	5
652	2	3	2	2	4	2	5	5
653	2	4	2	2	4	3	2	5
654	2	4	2	2	5	1	4	5
655	2	2	2	2	1	3	4	5
656	2	4	2	1	5	3	2	3

657	2	3	2	2	4	3	2	4
658	2	3	2	2	2	2	5	5
659	2	2	2	2	1	2	4	4
660	2	3	1	2	1	2	3	4
661	2	2	1	2	1	2	3	4
662	2	2	1	2	1	3	4	5
663	2	3	2	1	4	3	4	4
664	2	2	2	2	1	3	3	4
665	2	3	2	2	3	3	4	5
666	2	2	2	2	1	2	2	5
667	2	2	2	2	2	4	3	4
668	2	3	2	1	2	3	2	4
669	1	2	1	2	1	2	4	4
670	2	3	2	1	3	4	2	4
671	2	4	2	1	5	2	3	5
672	2	3	2	2	3	2	3	5
673	2	2	2	2	1	3	3	5
674	2	2	1	2	1	2	3	5
675	2	2	1	2	1	2	3	5
676	2	5	2	1	5	2	3	4
677	2	2	1	2	2	2	5	5
678	2	3	2	1	3	2	3	4
679	2	3	2	2	2	2	3	5
680	2	4	2	1	5	3	2	4
681	2	3	1	2	2	2	3	4
682	2	2	2	2	1	3	3	3
683	2	2	2	2	1	2	5	5
684	2	2	1	2	1	4	5	5
685	2	2	2	2	1	4	3	3
686	2	3	1	2	3	3	2	3
687	2	2	1	2	1	2	3	4
688	2	2	2	2	1	4	2	5
689	2	4	2	1	5	3	3	5
690	2	2	2	2	1	3	3	5
691	2	3	2	2	2	4	1	5
692	2	2	2	2	1	3	3	3
693	2	2	1	2	1	3	4	5
694	2	4	1	1	5	3	3	3
695	2	3	2	2	3	3	1	4
696	2	2	2	2	1	3	3	5
697	2	2	2	1	1	2	4	5
698	2	3	2	2	2	3	2	5
699	2	2	2	2	1	3	3	4
700	2	4	2	1	5	4	2	5
701	2	4	1	2	5	3	2	3
702	2	2	2	2	2	4	2	4
703	2	3	2	2	2	2	3	4
704	2	3	2	2	3	4	3	4
705	2	3	2	2	4	3	2	5
706	2	3	1	2	2	2	4	5
707	1	3	2	2	3	4	4	3
708	2	3	2	2	2	3	5	4
709	2	2	2	2	1	2	4	4
710	2	3	2	2	3	4	2	5
711	2	3	2	2	2	2	3	4

712	2	3	2	2	3	3	2	5
713	2	4	2	1	5	2	4	5
714	2	3	2	2	2	3	4	3
715	2	2	1	2	1	3	3	4
716	2	3	2	2	3	4	3	4
717	2	4	1	1	5	1	5	5
718	2	3	2	1	2	2	3	5
719	2	2	2	2	2	2	4	5
720	2	2	2	2	1	3	4	4
721	2	2	2	2	2	2	5	5
722	2	2	2	2	2	4	1	3
723	2	2	2	2	1	3	3	5
724	2	4	1	2	5	3	4	5
725	2	4	1	2	5	2	2	3
726	2	2	1	2	1	3	3	4
727	2	3	2	2	4	3	2	5
728	2	4	2	2	5	2	3	4
729	2	2	2	2	2	3	4	5
730	2	5	2	1	5	3	3	4
731	2	2	2	2	1	2	3	4
732	2	2	2	2	1	3	3	5
733	2	3	2	2	2	3	4	5
734	2	2	1	2	1	2	3	5
735	2	4	2	1	5	2	3	5
736	1	2	2	2	1	4	1	4
737	2	2	2	2	1	3	4	5
738	2	4	2	1	5	2	4	3
739	2	3	2	2	2	4	4	4
740	2	2	2	2	1	3	3	4
741	1	2	2	2	1	3	3	5
742	2	3	2	2	4	3	2	2
743	2	3	2	2	2	2	4	5
744	2	4	2	1	5	3	4	4
745	1	2	1	2	1	3	4	3
746	2	4	2	1	5	3	3	4
747	2	2	2	2	1	2	4	5
748	2	3	2	2	2	4	2	5
749	1	2	2	2	1	3	4	5
750	2	2	1	2	2	3	2	4
751	2	2	2	2	2	4	2	4
752	2	3	2	2	3	3	3	5
753	2	2	2	2	2	2	4	4
754	2	4	2	1	5	3	4	5
755	2	2	1	2	1	2	4	4
756	2	2	2	2	1	2	3	5
757	2	3	2	2	2	4	2	1
758	2	2	2	2	2	4	3	4
759	2	3	2	2	2	3	2	4
760	2	2	2	2	2	3	2	4
761	2	3	2	2	3	3	3	3
762	2	3	2	2	3	3	1	4
763	2	3	2	2	2	2	3	5
764	2	3	2	2	2	2	5	5
765	2	3	1	2	2	2	4	3
766	2	3	2	2	2	3	3	4

767	2	3	2	2	2	2	3	3
768	2	3	2	2	2	3	3	4
769	2	3	1	2	2	3	3	4
770	2	2	2	2	2	2	2	4
771	2	2	2	2	1	3	3	5
772	2	3	2	2	3	3	1	5
773	2	3	2	2	3	4	2	4
774	2	3	2	2	3	4	1	3
775	2	2	1	1	1	2	3	4
776	2	3	2	2	2	3	3	4
777	2	4	2	1	5	2	5	4
778	2	3	2	2	3	3	3	5
779	2	4	2	2	4	3	4	4
780	2	3	2	2	3	3	4	5
781	2	3	2	2	2	2	3	4
782	2	2	2	2	1	3	4	4
783	2	4	2	1	5	4	4	5
784	2	2	2	2	2	4	3	3
785	2	4	2	1	5	2	5	4
786	2	3	2	2	2	2	3	5
787	2	2	2	2	2	3	3	5
788	2	2	2	2	2	3	3	4
789	1	3	2	2	2	4	3	4
790	2	2	2	2	2	3	2	3
791	2	2	2	2	2	2	4	5
792	2	4	2	1	5	4	3	3
793	2	2	2	2	1	4	3	4
794	2	3	2	2	2	3	3	3
795	2	2	2	2	1	3	4	5
796	2	3	1	2	3	2	3	4
797	2	3	2	2	2	2	5	5
798	2	2	1	2	2	3	3	5
799	2	3	2	2	3	2	5	5
800	2	3	2	2	2	3	2	5
801	2	3	2	2	4	2	2	2
802	2	3	2	2	2	3	3	5
803	2	2	2	2	1	3	2	5
804	2	2	2	2	1	2	4	4
805	2	3	2	2	2	2	3	5
806	2	4	2	2	4	3	3	5
807	2	3	2	2	2	3	4	4
808	2	3	2	2	2	2	4	4
809	2	2	2	2	1	2	3	4
810	2	2	2	2	1	3	3	5
811	2	4	2	1	5	2	4	3
812	2	2	2	2	1	3	3	5
813	2	4	1	1	5	3	3	4
814	2	4	2	2	4	3	4	4
815	2	4	2	1	5	3	4	4
816	2	3	2	2	2	2	5	5
817	2	3	2	2	4	4	3	4
818	2	2	2	2	1	2	4	5
819	2	2	2	2	1	3	2	5
820	2	3	2	2	2	3	4	5
821	1	2	2	2	1	3	4	5

822	2	3	2	2	3	3	3	5
823	2	3	2	2	2	4	2	4
824	2	3	2	2	3	3	4	4
825	2	3	2	2	2	3	3	4
826	2	3	2	2	3	3	3	3
827	2	2	2	2	1	3	3	4
828	2	2	1	2	1	2	3	5
829	2	2	2	2	1	4	3	5
830	2	3	2	2	2	4	2	4
831	2	3	1	2	3	3	3	3
832	2	2	2	2	1	3	3	5
833	1	3	2	2	3	4	3	4
834	2	4	2	1	5	3	4	5
835	2	3	2	2	2	4	1	3
836	2	3	2	2	3	2	4	4
837	2	2	2	2	1	3	3	4
838	2	3	2	2	2	3	3	3
839	2	2	1	2	2	3	4	5
840	2	4	2	1	5	3	4	3
841	2	2	2	2	2	3	2	4
842	2	2	2	2	1	4	2	3
843	2	3	1	2	2	3	4	5
844	2	2	1	2	1	3	3	5
845	2	2	2	2	1	2	4	4
846	2	5	2	1	5	3	4	4
847	2	3	2	2	4	2	2	5
848	2	2	2	2	2	2	3	5
849	2	4	2	1	5	3	2	4
850	2	3	3	1	1	2	4	4
851	1	2	2	2	1	2	5	5
852	2	2	2	2	1	2	4	5
853	2	2	2	2	1	2	3	5
854	2	3	2	2	4	3	4	5
855	2	2	2	2	1	3	3	5
856	2	4	2	1	5	3	4	3
857	2	2	1	2	1	3	4	4
858	2	3	1	2	2	2	4	5
859	2	4	2	1	5	3	2	3
860	2	3	2	2	4	4	3	4
861	2	4	2	1	5	3	2	3
862	2	3	2	2	4	3	3	3
863	2	2	2	2	2	2	3	5
864	2	4	2	2	4	3	2	4
865	2	3	2	2	3	2	4	5
866	2	2	2	2	1	3	2	5
867	2	2	2	2	1	4	2	4
868	2	2	1	2	2	3	2	3
869	2	3	2	2	3	3	3	5
870	2	4	2	1	5	2	4	4
871	2	3	2	2	2	3	3	4
872	2	5	2	1	5	3	4	3
873	2	3	2	2	2	3	4	5
874	2	2	2	2	1	2	4	5
875	2	3	2	2	3	3	3	4
876	2	4	1	1	5	3	2	5

877	1	2	1	2	1	2	4	5
878	2	3	2	2	3	3	3	5
879	1	2	2	2	2	3	4	3
880	2	2	2	2	1	2	4	4
881	2	3	2	2	4	2	3	3
882	2	3	2	2	3	4	3	4
883	2	2	2	2	1	3	3	5
884	2	2	2	2	1	4	2	4
885	2	3	2	2	3	3	3	5
886	2	2	2	2	1	4	3	4
887	2	5	2	1	5	2	4	5
888	2	4	2	1	5	2	2	5
889	2	2	2	2	1	4	2	4
890	2	4	2	1	5	3	3	3
891	2	5	2	1	5	3	4	5
892	1	2	2	2	2	3	5	5
893	2	4	2	1	4	2	3	5
894	1	2	2	2	1	4	3	5
895	2	4	1	1	5	2	3	5
896	2	4	2	1	5	4	4	4
897	2	3	2	2	3	2	3	5
898	2	4	2	2	4	3	2	4
899	2	3	2	2	1	4	2	4
900	2	5	2	1	5	3	4	4
901	2	2	2	2	2	3	3	5
902	1	2	2	2	1	3	3	5
903	2	2	1	2	1	2	3	4
904	2	3	2	2	3	2	2	3
905	2	2	2	2	2	3	2	4
906	2	2	2	2	1	3	2	4
907	2	2	1	2	2	4	2	4
908	2	3	1	2	2	4	3	5
909	2	3	2	2	2	4	2	4
910	2	3	2	2	2	3	3	4
911	2	2	2	2	1	3	2	5
912	2	2	2	2	1	3	3	4
913	2	3	1	2	1	1	5	5
914	2	3	2	2	2	3	3	5
915	2	2	2	2	2	2	3	5
916	1	2	2	2	1	3	2	5
917	2	2	2	2	1	2	2	4
918	2	4	2	1	5	3	3	4
919	1	2	2	2	1	3	3	5
920	2	2	2	2	2	2	3	3
921	2	2	2	2	2	2	3	5
922	2	2	2	2	2	3	3	5
923	2	3	2	2	2	3	2	3
924	2	2	2	2	2	3	3	4
925	2	3	2	2	3	3	3	3
926	2	4	2	2	4	3	3	5
927	2	4	2	1	5	3	3	5
928	2	2	2	2	2	3	3	4
929	1	4	2	1	5	3	3	4
930	2	3	2	2	2	2	3	5
931	2	4	2	1	5	4	4	5

932	2	3	2	2	2	3	2	4
933	2	2	2	2	1	3	4	4
934	2	2	2	2	1	2	3	4
935	2	2	2	2	1	4	2	4
936	2	3	2	2	2	4	2	5
937	2	3	2	2	2	3	2	5
938	2	3	2	2	3	3	3	4
939	2	3	2	2	3	4	2	4
940	1	2	2	2	1	3	2	4
941	2	3	2	2	4	1	4	5
942	2	2	2	2	1	3	3	5
943	2	3	2	2	3	2	4	5
944	2	3	2	2	3	2	4	5
945	2	3	2	2	4	2	4	5
946	2	3	2	2	3	3	4	5
947	2	3	2	2	3	2	3	5
948	2	2	2	2	1	3	4	4
949	2	2	2	2	1	4	2	3
950	2	2	1	2	1	2	4	4
951	2	3	2	2	4	3	2	5
952	2	3	2	2	3	3	3	4
953	2	3	2	2	4	3	2	5
954	2	3	2	2	2	3	5	5
955	2	2	2	2	2	3	4	5
956	2	3	2	2	2	4	3	4
957	1	2	1	2	1	4	2	5
958	2	2	2	2	2	2	2	4
959	2	2	2	2	2	1	4	5
960	2	3	2	2	3	2	2	5
961	2	2	2	2	1	2	4	4
962	2	3	2	2	4	4	3	3
963	2	3	2	2	2	3	3	5
964	2	3	2	2	2	3	3	4
965	2	2	2	2	1	3	2	4
966	2	2	2	2	1	3	3	5
967	1	3	2	2	3	3	4	3
968	2	3	2	2	3	3	3	4
969	1	2	1	2	1	3	2	3
970	2	3	2	2	2	2	3	4
971	2	3	2	1	5	4	4	4
972	2	2	2	2	1	2	4	5
973	2	3	2	2	2	2	4	5
974	2	3	2	2	2	3	4	5
975	2	2	2	2	2	2	4	5
976	2	3	2	2	4	2	3	5
977	2	4	2	2	5	1	4	5
978	2	3	2	2	2	2	3	4
979	2	2	2	2	1	3	3	5
980	2	2	2	2	1	4	1	4
981	2	2	2	2	5	2	5	5
982	2	2	1	2	1	3	3	5
983	2	2	2	2	1	2	3	5
984	2	3	2	2	2	3	3	5
985	2	2	2	2	1	3	4	5
986	2	3	2	2	2	4	1	4

987	2	2	2	2	1	4	1	4
988	2	2	2	2	1	3	2	4
989	2	2	2	2	2	2	3	4
990	2	3	2	2	2	3	2	5
991	2	2	1	2	2	2	4	5
992	2	3	2	2	4	3	2	3
993	2	2	2	2	2	4	3	4
994	2	2	1	2	1	3	4	4
995	2	3	2	2	3	3	2	5
996	1	3	2	2	3	4	3	5
997	2	2	2	2	1	2	4	5
998	2	2	2	2	1	3	4	5
999	2	3	2	2	3	3	4	4
1000	2	4	2	1	5	3	4	5
1001	2	4	2	1	5	3	4	5
1002	2	4	3	1	5	4	3	5
1003	2	4	2	1	5	3	3	5
1004	2	4	2	1	5	3	4	4
1005	2	3	2	2	3	3	3	5
1006	2	3	2	2	2	2	4	5
1007	2	3	2	2	2	3	2	5
1008	2	4	2	1	5	3	2	4
1009	2	3	2	2	2	2	3	5
1010	2	2	1	2	1	4	2	4
1011	2	4	2	2	5	3	4	5
1012	2	4	2	1	5	3	3	5
1013	2	3	2	2	5	2	4	5
1014	1	2	2	2	1	4	2	4
1015	1	3	1	2	1	1	3	5
1016	2	3	2	2	3	3	3	5
1017	2	3	2	2	2	2	3	4
1018	2	2	2	2	1	4	2	4
1019	2	4	2	1	5	4	3	4
1020	2	3	2	2	2	3	2	5
1021	2	4	2	1	5	4	3	4
1022	2	2	2	2	1	3	2	5
1023	2	2	2	2	1	4	3	3
1024	2	2	2	2	1	3	4	5
1025	2	4	2	1	4	3	2	4
1026	2	2	2	2	1	2	4	5
1027	2	4	2	2	4	3	4	3
1028	2	3	2	1	3	4	2	4
1029	2	4	2	1	5	3	3	5
1030	2	3	2	2	3	4	3	5
1031	2	3	2	2	4	2	2	5
1032	2	4	2	1	4	3	4	5
1033	2	4	2	1	5	3	4	4
1034	2	3	2	2	2	4	2	4
1035	2	5	2	1	5	3	4	4
1036	2	3	2	1	2	3	3	5
1037	2	3	2	2	4	2	3	5
1038	2	4	2	1	5	3	2	5
1039	2	2	2	2	2	4	2	2
1040	2	3	2	2	2	2	2	4
1041	2	3	1	2	4	3	3	4

1042	2	2	2	2	2	4	3	5
1043	2	4	2	2	5	3	3	5
1044	2	3	2	2	3	4	4	4
1045	2	2	1	2	1	2	5	4
1046	2	4	2	1	5	3	4	3
1047	2	5	2	1	5	3	4	3
1048	2	2	2	2	1	3	4	4
1049	2	3	2	2	2	2	4	5
1050	2	3	2	2	4	4	3	5
1051	2	4	2	1	5	3	2	5
1052	2	3	2	1	4	3	4	4
1053	2	3	2	2	2	3	3	5
1054	2	3	2	2	3	4	4	5
1055	1	2	1	2	1	2	2	5
1056	2	2	2	2	2	2	3	5
1057	2	5	2	1	5	3	4	2
1058	2	4	2	2	5	3	3	4
1059	2	3	2	2	2	2	4	4
1060	2	3	2	2	3	3	2	4
1061	2	5	3	1	5	3	4	3
1062	2	4	2	1	5	3	3	5
1063	2	3	1	1	5	4	2	3
1064	2	5	2	1	5	4	3	4
1065	2	2	2	2	2	2	4	5
1066	2	3	2	2	3	3	1	5
1067	2	5	2	1	5	4	2	5
1068	2	4	2	1	5	2	2	5
1069	2	3	2	2	3	3	4	4
1070	2	2	1	2	1	3	3	5
1071	2	4	2	1	5	2	3	5
1072	2	3	2	1	4	4	1	4
1073	2	2	1	2	1	2	5	4
1074	2	3	2	2	3	4	2	4
1075	1	2	1	2	1	3	4	5
1076	2	2	1	2	2	2	4	5
1077	2	2	2	2	1	3	3	5
1078	2	3	2	2	2	3	4	5
1079	2	2	1	2	1	2	4	5
1080	1	2	2	2	1	3	4	4
1081	2	2	1	2	1	2	3	4
1082	2	3	2	2	4	3	3	4
1083	2	3	2	2	3	2	4	5
1084	2	5	2	1	5	3	3	5
1085	2	2	2	2	1	2	4	5
1086	2	3	2	2	2	3	3	4
1087	2	4	2	2	4	2	3	4
1088	2	2	2	2	2	2	2	4
1089	2	4	2	1	5	3	3	4
1090	2	2	2	2	1	2	5	5
1091	2	3	2	2	3	2	3	3
1092	2	3	2	2	2	2	3	4
1093	2	3	2	2	2	3	3	4
1094	2	3	2	2	4	4	2	3
1095	2	3	2	2	2	3	3	4
1096	2	2	1	2	2	3	2	5

1097	2	2	2	2	2	3	3	4
1098	2	3	2	2	4	3	4	5
1099	2	3	2	2	3	2	4	4
1100	2	3	2	2	2	3	3	3
1101	2	3	2	2	4	4	3	5
1102	2	3	2	2	2	3	2	4
1103	2	4	2	1	5	3	3	4
1104	2	2	2	2	2	4	4	5
1105	2	2	2	2	1	2	4	5
1106	2	3	2	2	3	3	2	5
1107	2	3	2	2	4	4	2	5
1108	2	3	2	2	4	2	3	2
1109	2	4	2	1	5	4	3	4
1110	2	3	2	2	2	3	3	5
1111	2	3	2	2	4	3	4	3
1112	2	3	2	2	2	4	3	4
1113	2	2	2	2	1	2	3	5
1114	2	2	1	2	1	1	5	5
1115	2	2	2	2	2	3	3	4
1116	2	3	2	2	2	2	4	4
1117	2	2	1	2	1	3	4	5
1118	2	3	2	2	2	2	3	4
1119	2	2	2	2	2	4	3	5
1120	2	2	2	2	2	3	2	3
1121	2	2	1	2	1	4	3	5
1122	2	3	2	2	4	4	3	5
1123	1	2	2	2	2	3	3	5
1124	2	2	2	2	1	2	2	3
1125	2	2	2	2	2	2	3	5
1126	2	4	2	2	4	2	3	4
1127	2	2	1	2	1	3	3	4
1128	2	3	2	2	4	3	4	4
1129	2	3	2	2	2	1	2	3
1130	2	2	2	2	2	3	3	5
1131	2	2	2	2	1	3	3	5
1132	2	4	2	1	5	3	3	5
1133	2	3	2	2	2	1	4	5
1134	2	2	1	2	1	3	5	5
1135	1	2	2	1	1	2	3	3
1136	2	2	2	2	1	3	5	5
1137	2	2	2	2	1	3	4	3
1138	2	2	2	2	2	2	3	3
1139	2	3	2	2	4	2	3	3
1140	2	3	2	2	2	3	3	5
1141	2	3	2	2	2	3	3	5
1142	2	2	2	2	2	2	4	5
1143	2	3	2	2	2	4	3	4
1144	2	3	2	2	4	2	1	4
1145	2	2	2	2	2	3	3	2
1146	2	4	2	1	5	3	4	3
1147	2	2	2	2	1	3	5	5
1148	1	2	2	2	1	3	1	5
1149	2	3	2	2	3	3	4	3
1150	2	3	2	2	3	3	2	4
1151	2	2	2	2	2	2	2	4

1152	2	5	2	1	5	2	4	5
1153	1	3	2	2	3	4	2	1
1154	2	2	1	2	1	1	5	5
1155	2	3	2	2	3	2	3	5
1156	2	3	2	2	3	3	3	5
1157	2	3	2	2	4	3	2	3
1158	1	2	2	2	1	2	4	5
1159	1	2	1	2	1	2	4	5
1160	2	3	2	2	3	3	3	4
1161	2	2	2	2	1	2	5	5
1162	2	4	2	1	5	3	4	4
1163	2	2	2	2	2	3	5	5
1164	1	2	1	2	1	3	4	5
1165	2	2	1	2	1	3	3	5
1166	2	5	2	1	5	3	5	5
1167	2	3	2	2	3	3	2	5
1168	1	3	2	2	2	3	4	5
1169	2	2	2	2	1	3	3	4
1170	2	2	1	2	1	2	4	5
1171	2	3	2	2	4	2	4	4
1172	2	3	2	2	2	2	4	5
1173	2	3	2	2	3	3	4	3
1174	2	3	2	2	4	3	3	5
1175	2	4	2	1	5	3	3	4
1176	2	3	2	2	2	3	3	4
1177	2	3	2	2	4	2	4	5
1178	2	3	2	2	2	4	2	4
1179	2	3	2	1	3	2	5	3
1180	2	2	2	2	1	2	3	5
1181	2	3	2	2	2	2	2	4
1182	2	3	2	2	4	3	2	1
1183	1	2	1	2	1	3	3	5
1184	2	4	3	1	5	3	2	4
1185	2	4	2	1	5	4	5	4
1186	1	2	2	2	1	3	4	5
1187	2	3	2	2	2	3	3	4
1188	2	3	2	2	2	3	4	4
1189	2	5	2	1	5	4	4	5
1190	2	3	2	2	2	4	2	5
1191	2	3	2	2	2	3	4	4
1192	2	3	2	2	3	3	4	5
1193	2	3	2	2	2	2	3	5
1194	2	2	2	2	1	2	4	4
1195	2	4	2	1	5	3	4	4
1196	2	3	2	2	2	4	1	5
1197	2	3	2	2	2	2	3	4
1198	2	4	2	1	5	3	3	4
1199	2	2	2	2	2	3	2	4
1200	2	2	2	2	1	2	4	4
1201	2	2	2	2	1	1	2	3
1202	2	4	2	1	5	3	4	4
1203	2	3	2	2	2	3	3	3
1204	2	3	2	2	3	2	4	5
1205	2	2	1	2	1	3	5	5
1206	1	2	1	2	1	4	4	5

1207	2	3	1	2	1	2	3	5
1208	2	3	2	2	3	4	2	4
1209	2	3	2	2	4	4	2	2
1210	2	2	2	2	2	1	4	5
1211	2	3	2	2	3	3	3	4
1212	2	2	2	2	1	3	3	5
1213	2	3	2	2	3	4	2	4
1214	2	3	2	2	3	3	3	4
1215	2	3	2	2	3	3	4	5
1216	2	2	1	2	1	3	5	5
1217	2	4	2	1	5	4	4	4
1218	2	3	2	2	3	2	4	4
1219	2	3	2	2	3	3	3	5
1220	2	2	2	2	1	2	2	4
1221	2	2	2	2	1	3	4	5
1222	2	3	2	2	4	4	1	4
1223	2	4	2	1	5	3	3	4
1224	2	3	2	2	4	3	2	2
1225	2	3	2	2	2	3	3	4
1226	2	2	1	2	1	4	4	5
1227	2	3	2	2	3	2	2	5
1228	2	4	2	2	5	3	2	4
1229	2	2	1	2	1	3	3	5
1230	2	3	2	2	3	4	2	4
1231	2	3	2	2	3	4	4	4
1232	2	4	2	1	5	3	2	4
1233	2	2	2	2	2	4	2	5
1234	2	2	2	2	1	3	4	5
1235	2	3	2	2	3	3	4	4
1236	2	3	2	2	3	3	2	5
1237	2	3	1	2	3	3	5	5
1238	1	3	2	2	2	3	3	3
1239	2	3	2	2	4	3	2	4
1240	2	3	1	2	3	3	3	4
1241	2	3	2	2	4	3	4	5
1242	2	3	2	2	3	2	3	4
1243	2	4	2	1	5	3	3	4
1244	2	3	2	2	3	2	4	4
1245	1	2	2	2	1	3	2	5
1246	2	4	2	1	5	3	4	3
1247	2	3	2	2	3	3	2	5
1248	2	2	1	2	1	2	2	3
1249	2	4	2	1	5	4	3	4
1250	2	3	2	2	3	4	3	4
1251	2	3	2	2	4	2	3	4
1252	2	2	1	2	1	3	2	4
1253	2	4	2	1	5	3	3	4
1254	2	4	2	2	4	3	3	3
1255	2	3	2	2	2	4	3	3
1256	2	3	2	2	2	4	3	5
1257	2	3	2	2	2	4	2	5
1258	2	2	2	2	1	2	3	4
1259	2	2	1	2	1	3	4	5
1260	2	5	2	1	5	3	4	3
1261	2	2	2	2	1	2	4	5

1262	2	2	2	2	2	3	4	5
1263	2	3	2	2	2	3	3	4
1264	2	3	2	2	2	2	3	4
1265	2	3	2	2	4	3	4	5
1266	2	3	2	2	4	3	4	4
1267	2	2	2	2	2	3	3	4
1268	2	2	2	2	2	4	4	4
1269	2	3	2	2	3	2	4	5
1270	2	5	2	1	5	4	3	3
1271	2	5	2	1	5	3	4	5
1272	2	5	2	1	5	3	3	4
1273	2	4	2	1	5	4	5	5
1274	2	3	2	2	3	2	3	5
1275	2	2	2	2	2	4	2	5
1276	2	3	2	2	2	3	4	4
1277	2	3	2	2	2	4	3	5
1278	2	3	2	1	4	2	3	5
1279	2	2	2	2	1	3	3	5
1280	2	2	1	2	1	3	3	5
1281	2	3	2	2	3	3	5	4
1282	2	4	2	1	5	4	3	5
1283	2	4	2	1	4	3	3	5
1284	2	5	2	1	5	4	2	4
1285	2	5	2	1	5	3	2	5
1286	1	3	2	2	3	4	2	3
1287	2	3	2	2	4	2	3	5
1288	2	5	2	1	5	3	4	4
1289	2	3	3	2	4	3	2	2
1290	2	3	2	2	2	2	3	4
1291	2	2	2	2	2	3	2	3
1292	2	3	2	2	3	2	3	5
1293	2	2	1	2	1	3	4	4
1294	2	3	2	1	4	2	3	5
1295	2	4	2	1	5	3	5	5
1296	1	3	2	2	3	3	3	3
1297	2	2	2	2	2	3	3	3
1298	2	3	2	2	4	3	2	5
1299	2	3	2	2	3	3	2	5
1300	2	3	2	2	3	3	3	5
1301	2	4	2	1	5	3	2	3
1302	2	3	2	2	2	3	3	2
1303	2	2	2	2	1	4	3	5
1304	2	3	2	2	2	3	4	5
1305	2	4	2	1	5	3	4	4
1306	2	4	2	2	5	3	3	4
1307	2	5	2	1	5	2	3	5
1308	2	4	2	1	5	4	3	5
1309	2	4	2	2	5	3	4	5
1310	2	2	2	2	1	2	4	5
1311	2	2	2	2	1	2	3	4
1312	2	3	2	2	2	3	3	4
1313	2	2	1	2	1	2	3	4
1314	2	5	1	1	5	2	4	5
1315	2	3	2	2	3	2	2	4
1316	2	4	2	1	5	4	3	4

1317	2	2	2	2	1	2	4	4
1318	2	4	2	2	4	3	2	5
1319	2	2	1	2	2	2	3	5
1320	2	3	2	1	4	4	3	5
1321	2	4	2	1	5	3	2	4
1322	2	4	2	1	5	2	4	4
1323	1	2	2	2	2	3	3	4
1324	2	2	2	2	2	3	1	4
1325	2	2	2	2	2	3	4	5
1326	2	2	2	2	1	3	4	4
1327	2	2	2	2	1	3	2	4
1328	2	5	1	1	5	4	3	4
1329	2	2	1	2	1	3	3	4
1330	2	3	2	2	3	3	1	4
1331	2	2	2	2	1	2	4	5
1332	2	2	2	2	1	3	4	5
1333	2	5	2	1	5	3	4	3
1334	2	2	2	2	1	3	3	5
1335	2	3	2	2	2	4	5	4
1336	2	3	2	2	2	3	3	5
1337	2	3	2	2	2	3	2	4
1338	2	4	2	2	5	1	5	5
1339	2	3	2	2	4	3	3	5
1340	2	3	2	2	3	3	3	4
1341	2	3	2	2	3	3	3	5
1342	2	2	2	2	2	4	2	3
1343	2	4	2	1	4	2	3	4
1344	2	4	2	1	4	3	2	3
1345	2	4	2	1	5	4	3	5
1346	2	4	2	1	5	3	4	5
1347	2	2	2	2	1	1	3	5
1348	2	3	2	2	4	3	4	5
1349	2	4	2	1	5	3	4	5
1350	2	5	2	1	5	4	3	4
1351	2	4	2	1	5	3	3	4
1352	2	4	2	1	5	3	4	4
1353	2	3	1	2	2	2	4	5
1354	2	2	2	2	1	3	4	4
1355	2	3	2	2	3	4	2	5
1356	2	2	2	2	2	3	3	4
1357	2	2	1	2	1	1	3	5
1358	2	2	2	2	1	3	2	5
1359	2	4	2	2	3	3	3	2
1360	2	2	2	2	1	3	4	5
1361	2	2	2	2	1	3	2	5
1362	2	3	2	2	2	2	2	4
1363	2	3	2	2	2	4	4	3
1364	2	3	2	2	2	4	2	3
1365	2	4	2	2	4	2	3	4
1366	2	3	3	1	5	4	3	4
1367	2	4	2	1	5	2	4	4
1368	2	3	2	2	2	3	3	5
1369	2	3	2	2	2	3	4	5
1370	1	3	2	2	2	4	4	4
1371	2	3	2	2	3	3	2	4

1372	2	4	2	1	4	3	3	4
1373	2	4	2	1	4	2	4	4
1374	2	5	2	1	5	3	2	5
1375	2	3	2	2	2	3	3	4
1376	2	3	2	2	2	2	4	5
1377	2	3	2	2	2	3	3	5
1378	2	3	2	2	2	4	2	4
1379	2	3	2	2	4	4	2	5
1380	2	3	2	2	3	2	4	5
1381	2	3	2	2	3	3	2	3
1382	2	2	2	2	2	4	4	5
1383	2	2	2	2	1	3	3	4
1384	2	3	2	2	3	2	4	5
1385	2	3	2	2	3	2	2	5
1386	2	3	2	2	4	3	2	5
1387	2	2	2	2	2	2	4	5
1388	2	3	2	2	2	2	3	5
1389	2	3	2	2	3	1	4	5
1390	2	2	2	2	2	3	2	5
1391	2	4	2	1	5	3	3	5
1392	2	3	2	2	4	3	2	3
1393	2	4	2	2	4	3	2	4
1394	2	3	2	2	2	2	4	4
1395	2	3	2	2	4	2	3	5
1396	2	2	2	2	1	3	2	5
1397	2	2	2	2	1	3	2	5
1398	2	2	2	2	1	4	2	5
1399	2	4	2	2	4	2	3	5
1400	2	3	2	2	4	3	1	4
1401	2	3	2	2	4	3	3	3
1402	2	3	2	2	2	3	4	5
1403	2	4	2	1	5	4	2	4
1404	2	3	2	2	3	2	4	5
1405	2	3	2	1	3	3	3	4
1406	2	2	2	2	1	3	2	5
1407	2	3	2	2	3	3	4	5
1408	2	3	2	2	3	3	2	4
1409	2	5	2	1	5	2	5	4
1410	2	3	2	2	3	3	2	4
1411	2	4	2	1	5	2	2	3
1412	2	4	2	2	5	2	3	3
1413	2	2	2	2	2	3	3	4
1414	2	3	2	2	4	3	2	4
1415	2	2	2	2	2	2	3	4
1416	2	4	2	1	5	4	4	4
1417	2	3	2	2	2	2	4	5
1418	2	3	2	2	3	1	4	5
1419	2	2	2	2	2	1	5	5
1420	2	4	2	2	4	3	4	5

JR2 (1=Never;2 =Seldom;3= Sometimes;	JR3 (1=Never;2 =Seldom;3= Sometimes;	JR4 (1=Never;2 =Seldom;3= Sometimes;	JR5 (1=Never;2 =Seldom;3= Sometimes;	JR6 (1=Never;2 =Seldom;3= Sometimes;	JR7 (1=Never;2 =Seldom;3= Sometimes;	JR8 (1=Never;2 =Seldom;3= Sometimes;	JR9 (1=Never;2 =Seldom;3= Sometimes;	JR10 (1=Never;2 =Seldom;3= Sometimes;
5	5	4	4	5	3	5	5	1
4	4	4	4	3	3	3	3	2
1	4	3	3	5	3	5	5	3
3	3	4	3	2	2	4	4	2
5	5	2	4	4	4	4	5	1
4	4	4	4	5	5	5	5	3
4	5	5	3	2	3	5	4	1
5	4	4	5	4	4	4	4	3
4	4	4	4	2	2	4	3	2
4	4	4	4	4	4	4	4	2
4	5	4	4	3	3	3	4	1
5	5	5	5	1	1	1	1	3
4	4	4	4	5	5	4	4	1
4	5	5	5	5	5	5	5	3
4	4	4	4	3	3	3	4	3
5	5	5	4	3	3	4	4	1
3	4	4	4	3	3	3	4	2
5	5	5	4	2	2	2	4	3
5	5	5	5	4	4	4	4	4
4	5	5	4	5	5	3	4	3
4	4	4	4	5	5	5	4	1
2	3	3	2	4	4	4	3	1
4	4	4	4	4	4	4	4	4
5	5	5	5	1	3	3	3	3
4	4	4	4	4	4	4	3	2
4	5	5	4	4	5	4	5	3
5	4	5	4	4	4	2	4	3
2	4	4	3	1	1	2	4	3
4	4	3	4	3	3	3	3	3
3	4	3	4	2	3	5	4	1
3	4	4	4	2	2	4	3	2
5	5	3	5	3	3	4	3	1
4	4	4	4	2	3	5	4	1
1	4	1	3	2	2	4	5	1
5	4	4	3	4	4	4	5	1
5	5	5	5	5	5	5	5	4
5	4	4	3	2	1	5	1	2
5	5	5	5	1	1	1	5	5
4	4	4	4	3	4	4	4	2
4	4	4	4	3	3	5	4	1
4	4	4	4	5	5	4	4	2
3	3	3	3	2	2	4	3	1
4	4	4	4	4	4	4	4	3
5	5	4	4	2	2	3	3	3
4	4	4	3	5	5	5	5	3
4	4	3	4	1	1	5	2	3
3	4	3	4	5	5	5	4	1
5	5	5	5	4	4	4	4	2
5	4	4	4	4	3	3	4	2
4	4	3	4	1	2	2	3	1
5	5	5	5	5	5	5	5	1

4	4	3	3	4	3	4	3	3
5	3	5	5	3	3	5	5	1
3	4	4	4	2	5	3	4	3
2	3	2	2	4	3	5	2	1
4	4	4	4	4	4	4	4	1
4	4	4	4	3	3	4	4	2
3	3	3	3	3	3	3	3	1
4	5	4	4	3	3	3	4	3
5	4	4	4	4	3	3	5	2
3	4	2	4	3	3	5	4	1
4	4	5	4	2	1	4	4	1
3	3	4	3	3	3	3	3	2
5	5	5	5	4	4	4	4	2
4	5	4	4	4	4	2	2	2
4	3	4	3	4	4	3	3	3
5	5	4	5	4	3	3	3	4
5	4	5	5	1	4	4	4	3
3	4	4	3	3	4	4	4	1
4	4	4	4	1	2	3	3	2
5	5	4	5	1	3	1	1	4
4	5	5	5	5	4	5	5	3
4	4	3	3	4	3	2	2	1
5	5	4	4	3	3	2	2	3
4	3	3	3	3	3	4	4	1
3	3	3	3	3	3	5	5	1
4	3	1	4	1	1	1	1	5
5	5	5	5	5	5	5	5	5
4	4	4	3	5	5	5	5	1
4	3	4	3	2	2	4	4	3
5	4	4	4	1	1	5	5	1
4	4	4	4	4	4	4	4	1
5	5	4	5	2	4	4	4	4
3	3	3	3	2	2	2	2	2
5	5	5	5	5	5	3	3	4
3	4	3	4	2	2	4	4	3
5	5	5	5	5	5	5	5	5
4	4	4	4	4	4	4	4	3
3	4	2	3	3	2	2	2	3
4	4	4	4	4	4	4	4	3
4	4	3	5	2	2	4	4	2
5	5	5	5	4	4	4	4	2
4	3	3	3	3	3	3	3	1
5	5	5	5	3	3	5	5	5
5	5	4	4	4	3	4	4	3
3	3	3	3	2	2	3	3	1
3	4	3	2	2	2	3	3	1
5	5	5	5	1	4	5	5	3
5	5	5	4	3	4	5	5	3
3	3	4	4	4	4	4	4	2
5	4	5	5	3	3	3	3	3
4	4	5	5	3	3	4	4	1
2	3	2	3	3	3	2	2	4
3	4	4	4	2	2	4	4	1
5	5	5	5	4	4	4	4	4
3	3	3	3	3	3	3	3	2

3	3	3	3	3	3	3	3	2
5	3	3	2	3	3	3	3	3
3	5	3	4	1	3	5	5	1
4	5	4	4	2	2	2	2	3
5	4	2	4	5	5	3	3	1
5	5	5	5	1	3	4	4	1
4	5	3	2	5	5	4	4	1
3	3	1	3	1	2	4	4	1
4	3	4	3	5	4	5	5	1
3	4	3	4	2	2	3	3	1
4	4	4	3	3	3	4	4	2
3	3	3	4	2	2	3	3	1
4	4	4	4	4	4	3	3	3
3	3	2	4	4	4	5	5	1
5	5	3	4	3	3	4	4	4
3	3	3	3	2	1	2	2	2
4	4	3	3	4	4	5	5	1
3	3	4	3	2	3	2	2	2
3	3	2	3	3	3	4	4	1
4	4	4	4	4	4	4	4	2
4	4	4	3	3	3	4	4	2
5	4	5	4	4	4	4	4	3
1	1	1	1	5	5	5	5	2
4	4	4	3	2	4	3	3	2
4	4	4	4	3	3	3	3	1
5	5	5	5	5	5	3	3	3
3	4	4	3	3	4	4	4	2
3	4	3	3	2	2	3	3	4
4	4	4	4	3	4	4	4	1
5	5	5	5	3	3	5	5	4
4	4	4	4	3	4	3	3	1
3	3	1	3	3	3	5	3	1
3	4	3	3	2	2	5	1	1
3	3	2	3	4	2	4	4	1
4	5	4	4	2	2	2	2	4
2	3	3	2	3	3	3	3	1
4	4	4	4	2	2	5	4	2
4	4	3	4	2	3	3	4	3
4	2	3	3	1	1	5	3	1
4	4	2	4	2	2	3	4	1
4	4	3	4	1	1	4	1	1
4	4	4	4	4	4	4	3	3
4	4	4	5	4	4	4	5	4
5	5	5	5	5	5	3	5	4
4	5	5	4	2	2	4	3	2
4	3	3	3	2	2	5	4	1
4	4	5	4	4	4	2	5	3
3	4	2	4	1	2	5	5	1
4	4	4	5	2	2	5	5	2
5	5	5	5	2	4	4	4	5
5	5	5	3	5	3	2	4	3
5	5	5	5	1	5	5	5	5
4	5	4	4	3	3	5	3	2
5	5	5	4	4	3	3	3	1
3	3	3	3	3	3	3	3	3

3	4	1	3	2	2	4	3	1
4	4	3	4	4	4	3	3	2
4	4	4	5	5	5	3	5	4
5	5	4	4	5	5	5	3	3
4	5	5	5	1	1	3	5	1
5	4	4	4	4	3	3	4	2
3	3	2	3	3	3	3	4	2
5	5	5	4	3	3	3	4	2
5	5	4	5	5	5	5	4	1
2	4	5	4	2	3	5	5	1
5	3	5	5	1	1	3	3	2
3	5	4	4	2	2	5	4	2
4	4	3	4	1	2	3	3	3
4	4	4	4	2	2	4	5	3
4	4	5	3	3	3	4	4	3
4	4	3	4	3	3	3	3	1
4	4	4	4	5	5	5	5	3
4	1	5	1	5	5	5	5	1
3	3	3	3	3	3	5	3	1
4	4	4	5	3	3	3	3	3
4	4	4	5	3	4	4	4	3
5	4	4	5	3	4	3	3	4
4	5	5	5	4	4	5	5	1
5	5	5	5	4	3	4	5	4
4	4	4	4	4	4	4	4	2
5	5	4	4	5	5	3	5	2
5	5	3	5	5	5	5	5	5
5	5	5	5	5	4	4	5	2
5	5	5	5	5	5	3	5	5
4	4	4	4	5	5	5	5	1
4	4	4	4	4	4	4	3	1
5	5	4	4	5	5	4	4	2
4	3	5	4	4	4	4	3	2
3	3	3	3	3	3	3	3	3
4	4	5	4	5	5	3	5	3
5	4	3	5	5	5	4	4	3
5	4	4	4	2	2	5	4	4
5	5	5	5	5	5	5	5	2
4	4	4	4	3	4	2	4	3
4	4	4	4	2	2	3	3	3
5	5	5	5	4	4	1	4	3
5	5	5	3	4	4	4	4	2
3	3	3	4	2	2	2	4	1
4	4	4	4	4	4	4	4	2
4	4	4	4	3	4	4	4	3
4	5	5	4	2	4	3	3	1
3	3	3	3	3	2	5	3	2
5	5	5	5	2	3	3	5	3
3	4	4	3	3	3	4	4	2
4	4	4	4	5	3	4	4	3
4	4	4	5	4	4	4	4	2
4	4	4	4	2	2	3	3	3
4	4	4	4	4	4	4	4	2
4	4	4	4	4	4	3	3	1
4	4	4	4	4	4	4	4	2

4	4	4	4	5	3	2	2	3
5	5	5	5	3	2	5	5	3
3	3	4	3	1	2	4	4	1
4	3	3	3	3	3	4	4	1
4	3	3	3	4	4	4	4	1
4	4	5	4	5	5	4	4	2
4	4	2	4	1	1	1	1	1
5	5	2	5	2	2	5	5	1
4	4	4	4	2	2	3	3	3
5	5	5	5	3	3	4	4	5
3	3	4	3	3	3	4	4	3
4	4	4	3	5	5	3	3	1
5	5	5	4	3	3	5	5	2
4	5	4	4	2	3	4	4	3
5	5	4	5	5	5	5	5	1
3	3	3	3	3	3	4	4	2
4	4	4	3	4	4	5	5	3
4	4	4	4	2	2	2	2	1
4	4	3	3	3	3	4	4	2
3	4	4	3	5	5	5	5	2
5	5	5	5	5	4	4	4	2
4	5	5	5	1	1	4	4	3
4	3	4	4	4	4	4	4	1
4	4	4	2	5	5	5	5	1
4	4	3	3	2	2	5	5	2
4	3	3	4	1	1	5	5	1
5	5	3	5	4	4	4	4	3
4	4	5	4	2	2	3	3	2
4	4	4	4	4	4	4	4	3
3	3	3	3	3	3	2	2	2
5	5	5	5	4	4	3	3	2
3	3	4	3	3	4	3	3	3
3	5	4	3	2	2	3	3	2
4	4	4	4	3	2	5	5	2
4	4	4	4	2	2	5	5	1
5	5	5	5	5	5	5	5	4
3	4	4	4	3	3	3	3	3
4	4	4	3	3	3	3	3	3
5	5	5	5	5	3	3	3	1
3	4	2	4	5	4	3	3	4
3	5	5	3	3	3	1	1	1
3	4	3	4	2	3	2	2	2
4	4	4	4	3	3	4	4	3
4	4	3	3	5	5	3	3	3
5	5	5	5	1	1	5	5	3
3	4	4	4	3	4	5	5	3
4	5	4	4	3	3	4	4	1
3	4	3	3	4	4	4	4	2
4	4	3	4	3	3	5	5	1
5	5	4	4	4	4	4	4	4
3	4	4	3	5	5	5	5	3
3	4	4	4	3	3	4	4	3
5	5	5	5	5	5	4	4	5
4	4	4	4	4	4	3	3	3
4	4	4	4	4	4	4	4	2

5	5	4	4	3	2	4	4	2
4	4	4	3	4	4	3	3	3
4	4	4	4	2	2	4	4	1
5	5	5	4	4	4	5	5	2
4	4	5	5	5	5	5	5	1
4	3	3	4	3	3	2	2	2
4	4	4	5	4	4	2	2	5
4	4	4	5	5	5	4	4	2
4	4	5	4	2	2	5	5	3
4	4	4	4	1	1	4	4	2
4	5	4	4	2	3	2	2	1
4	4	4	5	3	3	3	3	3
5	5	5	3	3	4	3	3	2
2	3	3	3	4	4	4	4	2
4	4	4	5	5	5	5	5	1
2	3	1	2	4	4	4	4	1
4	4	4	2	3	4	4	4	2
4	4	4	4	3	3	4	4	1
5	5	4	4	4	2	3	4	3
4	5	3	3	2	2	5	3	4
4	4	4	4	3	3	3	3	3
5	4	4	5	5	5	5	5	2
5	5	5	5	4	5	3	4	3
5	5	5	5	4	4	3	4	5
4	4	4	4	2	3	4	4	2
3	4	3	3	3	3	3	3	3
5	5	4	5	4	2	4	2	4
5	5	5	5	5	5	4	5	5
4	3	3	3	4	4	4	4	3
4	4	3	3	2	3	3	4	2
5	4	5	5	2	2	2	2	3
5	5	5	5	4	4	3	4	3
5	5	4	5	4	4	4	4	3
4	4	4	4	2	3	3	5	1
4	4	4	4	4	4	3	4	2
4	4	4	5	4	4	5	5	3
4	4	4	4	2	4	4	4	2
4	4	2	3	2	2	5	2	1
5	5	5	5	3	4	3	4	3
5	5	5	5	5	5	5	5	1
5	3	4	5	5	5	5	5	4
4	4	4	4	4	4	4	4	3
4	3	3	3	3	3	3	4	2
4	4	3	3	2	3	4	4	1
4	4	4	4	4	3	4	3	3
4	4	4	4	5	5	2	5	1
4	4	4	4	2	3	4	4	1
4	3	5	5	5	5	5	5	1
4	3	4	3	4	5	4	3	2
5	5	5	5	4	4	4	4	5
5	5	5	5	5	5	5	5	2
2	3	3	3	3	3	3	3	1
3	3	2	3	2	3	3	5	1
5	4	3	5	5	5	4	5	5
4	3	4	4	1	2	4	3	2

4	4	4	4	3	4	5	3	2
4	4	4	4	3	3	3	3	3
4	3	4	5	2	4	4	4	3
5	4	4	5	4	4	5	4	1
3	3	4	3	4	4	4	4	2
4	4	4	3	3	3	4	5	2
5	5	4	5	2	4	4	3	3
3	5	4	3	5	3	3	3	1
4	4	3	3	4	4	5	4	3
5	5	5	5	2	3	4	3	3
4	4	4	4	5	5	4	2	3
4	4	5	4	3	3	2	4	2
4	4	4	3	4	2	3	3	2
4	4	4	4	3	4	4	5	4
4	5	4	4	4	5	5	4	2
3	4	4	3	2	2	4	3	1
5	5	5	5	5	5	4	5	5
4	4	4	4	5	5	5	3	1
4	4	4	4	5	5	5	3	3
4	4	4	4	5	5	3	4	3
4	4	4	4	2	3	3	4	1
5	5	5	5	5	5	3	5	3
5	5	5	5	5	5	1	5	2
4	4	4	5	3	3	4	5	1
4	4	3	3	2	2	4	5	1
4	4	4	4	3	3	4	2	1
4	3	4	4	2	2	2	2	4
5	5	5	5	4	4	3	4	3
4	4	4	4	2	2	2	2	1
5	5	5	5	5	5	5	4	2
5	5	5	5	5	5	5	5	4
5	5	5	5	5	5	4	5	5
4	4	4	4	3	3	2	2	4
4	4	4	4	3	3	3	3	1
5	5	5	5	1	2	3	2	2
3	4	4	5	2	3	3	3	1
5	5	5	5	4	4	3	3	5
5	5	5	5	5	5	5	5	2
4	4	4	4	3	5	3	4	3
4	5	5	5	5	5	4	3	1
5	5	5	5	5	4	2	5	2
4	4	4	4	4	4	3	4	2
5	5	4	5	5	5	4	5	3
4	4	4	4	3	4	3	2	2
5	5	5	5	2	2	3	3	2
3	4	4	4	4	4	5	4	3
4	4	4	4	3	3	4	3	4
3	4	3	3	3	2	5	3	1
5	5	5	5	5	5	5	5	1
5	5	5	5	4	4	4	5	1
4	4	4	4	3	4	3	3	2
4	5	4	4	5	2	5	4	3
5	5	5	5	1	3	3	5	1
5	5	5	5	5	5	4	4	1
5	5	5	5	5	5	5	4	3

5	4	4	4	2	4	4	4	1
4	5	4	4	4	4	3	3	3
3	3	3	2	3	3	5	3	1
4	4	4	4	5	3	3	4	2
3	4	4	2	3	3	3	3	2
2	3	1	3	3	3	5	3	1
4	4	3	4	1	2	3	4	1
4	5	4	4	2	4	5	3	2
4	4	3	4	2	3	4	2	2
3	2	4	4	4	4	3	3	1
4	4	4	4	2	2	2	4	2
2	4	1	2	4	2	4	2	1
1	2	2	2	5	4	5	4	1
5	5	5	5	5	3	1	5	4
5	5	5	5	3	3	3	5	3
2	4	3	2	4	3	4	4	1
5	5	4	5	5	5	4	5	2
4	4	2	4	5	5	5	5	1
2	4	4	3	2	1	4	4	2
4	4	4	4	3	3	3	3	2
5	5	5	4	2	3	3	3	5
5	5	5	4	2	2	4	4	1
4	3	5	1	2	3	3	3	2
5	5	5	5	4	4	2	2	2
5	5	4	5	5	5	5	5	3
3	3	3	4	3	4	5	5	1
4	5	4	3	3	3	5	5	5
5	5	3	4	5	1	1	1	1
3	3	3	3	3	3	3	3	3
4	4	5	5	5	5	3	3	3
3	3	3	2	2	2	4	4	1
2	3	3	4	3	3	4	4	1
4	4	3	4	2	2	3	3	2
4	5	4	4	5	5	5	5	1
4	4	5	5	5	3	2	2	3
5	5	5	5	5	5	3	3	3
4	4	4	4	3	3	3	3	1
5	5	5	5	4	4	4	4	3
5	4	2	4	3	3	3	3	2
5	5	4	5	4	4	4	4	1
5	5	5	5	5	5	5	5	1
3	3	3	3	4	3	5	5	1
4	4	3	4	3	3	3	3	2
4	5	5	5	1	5	3	3	3
5	3	4	5	1	2	2	2	2
4	4	4	4	3	3	4	4	3
5	5	5	5	1	1	5	5	1
5	4	5	5	5	5	2	2	3
4	4	1	4	2	2	5	5	1
4	4	2	4	2	3	4	4	2
4	4	4	4	4	4	4	4	2
3	4	4	3	5	4	5	5	1
5	5	5	5	4	5	4	4	4
4	4	4	4	4	4	4	4	3
4	4	3	4	5	5	5	5	1

4	4	2	4	2	3	4	4	2
4	4	4	4	5	5	5	5	1
4	4	4	4	2	2	3	3	3
4	4	4	4	3	3	5	5	1
4	4	4	4	5	5	3	3	3
3	5	5	5	1	2	5	5	1
4	4	4	4	3	3	4	4	3
4	5	5	3	2	2	2	2	3
4	4	4	5	5	3	5	5	3
4	4	2	3	2	2	5	5	1
5	5	3	4	2	3	5	5	1
3	4	4	4	4	4	5	5	1
4	4	4	4	2	3	3	3	3
5	5	5	5	3	3	3	3	1
4	4	4	4	5	4	4	4	3
4	5	5	5	1	3	3	3	5
4	4	3	4	4	4	4	4	1
4	4	5	4	5	5	5	5	2
5	5	5	5	5	5	3	3	3
4	5	1	4	4	4	4	4	1
4	4	4	4	2	2	4	4	3
3	4	4	3	4	4	3	3	1
4	5	4	4	1	2	5	5	2
4	4	4	4	3	4	4	4	1
4	4	4	4	2	2	5	5	1
4	4	4	4	4	4	4	4	1
4	4	4	4	5	3	3	3	2
5	5	5	5	1	1	1	1	1
4	4	4	4	3	3	2	2	2
4	4	4	4	3	3	3	3	3
3	4	4	4	5	1	4	4	1
4	4	3	4	2	2	2	2	4
4	4	4	4	2	2	5	5	1
4	4	3	4	2	2	4	4	2
4	5	1	4	2	3	5	5	3
3	3	3	4	3	3	3	3	3
2	4	4	4	4	4	4	4	3
4	5	4	3	3	4	4	4	1
4	4	4	4	2	2	2	2	1
2	3	3	2	2	3	4	4	2
4	5	5	5	4	4	4	4	3
3	3	4	3	2	3	3	3	3
5	3	4	4	5	5	5	5	3
4	3	3	3	4	4	3	3	2
3	3	3	3	5	3	5	5	1
4	4	4	4	4	4	4	4	3
5	5	4	5	1	1	3	3	1
3	3	3	3	1	2	4	4	3
4	4	4	4	5	5	3	3	1
4	5	4	4	3	3	4	4	3
3	3	3	3	3	3	3	3	3
3	3	3	3	3	3	3	3	3
5	5	3	5	3	4	2	2	1
4	4	4	4	2	3	4	4	1
3	3	3	4	5	5	5	5	3

4	5	4	4	3	3	4	4	2
3	4	4	3	3	3	5	5	3
4	4	4	4	2	2	2	2	2
5	5	5	5	1	1	1	1	5
4	4	4	3	1	3	5	5	2
5	5	5	5	4	5	5	5	3
4	4	4	4	5	2	5	5	2
2	3	2	2	2	2	4	4	1
5	4	4	5	2	4	3	3	2
4	5	4	5	5	5	5	5	3
5	5	5	5	5	5	3	3	3
3	3	2	2	2	3	4	4	1
3	3	3	4	2	3	2	2	2
4	5	3	3	3	3	4	4	1
5	5	5	5	4	4	3	3	3
4	5	4	4	5	5	5	5	3
3	3	3	3	3	3	3	3	3
3	5	3	3	3	3	5	5	1
4	4	4	4	4	4	3	3	1
5	5	3	5	3	3	2	2	3
5	5	4	5	5	5	3	3	1
4	4	4	4	5	5	5	5	2
4	3	3	3	1	1	4	4	2
5	5	5	4	4	4	3	3	4
4	4	3	3	2	3	3	3	2
4	4	4	4	2	2	2	2	2
5	5	5	5	3	3	4	4	2
5	5	5	5	3	3	3	3	2
4	4	4	4	3	3	3	3	1
4	4	3	3	3	3	3	3	3
4	5	5	5	5	5	3	3	2
4	4	4	5	4	4	3	3	3
4	2	3	3	3	3	2	2	3
4	4	4	4	5	5	4	4	4
4	5	4	5	2	3	3	3	2
4	5	5	5	4	4	4	4	5
5	4	5	4	4	4	4	4	3
2	3	4	3	3	3	4	4	1
5	5	4	4	5	3	4	4	2
4	4	4	3	2	2	5	5	2
4	4	4	4	3	3	3	3	3
4	5	4	4	2	2	2	2	1
4	4	4	4	2	3	4	4	3
5	5	5	5	1	1	1	1	1
3	5	4	4	3	4	4	4	1
5	5	5	5	5	5	3	3	3
4	4	5	4	5	4	4	4	3
5	4	4	5	2	3	5	5	4
2	4	1	3	5	3	3	3	2
3	3	4	4	4	4	4	4	2
4	4	1	3	1	1	1	1	2
4	4	5	4	2	2	4	4	1
4	4	4	4	3	3	3	3	1
3	4	3	3	2	2	4	4	2
4	5	2	4	1	3	3	3	2

5	5	3	5	1	1	5	5	3
5	5	3	5	1	1	5	5	3
5	5	2	5	1	3	3	3	3
5	5	4	5	5	3	5	5	4
3	3	4	3	3	4	2	2	2
5	5	5	5	3	4	3	3	3
3	4	3	4	2	3	4	4	2
4	4	4	5	3	2	3	3	1
5	5	5	5	4	4	2	2	3
5	5	5	5	4	5	4	4	2
3	4	4	3	3	3	4	4	2
4	4	4	4	4	4	4	4	3
4	4	4	4	5	4	5	5	1
5	5	4	4	2	4	4	4	2
4	4	4	4	4	4	4	4	1
5	5	5	5	3	3	3	3	3
4	4	4	4	2	3	3	3	3
5	5	5	5	1	2	4	4	3
4	4	4	4	3	3	2	2	1
4	5	5	4	5	3	5	5	1
3	3	3	3	3	3	3	3	3
3	5	5	4	3	3	3	3	2
5	5	5	5	5	5	5	5	5
4	4	4	4	3	5	5	5	1
3	4	4	4	4	4	4	4	2
3	3	3	3	2	2	4	4	1
4	5	5	5	5	5	5	5	3
4	3	4	4	3	3	5	5	1
5	5	5	4	5	5	5	5	2
5	5	5	5	5	5	5	5	4
4	4	4	4	2	2	5	5	3
5	5	5	5	1	4	5	5	2
4	3	2	4	2	3	5	5	2
5	5	5	5	4	3	5	5	3
4	4	4	4	3	3	3	3	2
2	2	2	3	2	2	4	4	1
4	4	4	4	1	4	3	3	1
5	5	3	5	5	3	2	2	2
4	4	4	4	2	2	4	4	2
4	4	4	4	2	3	4	4	3
4	3	3	4	2	2	2	2	3
4	5	4	4	5	5	5	5	1
4	5	4	4	2	4	4	4	2
5	5	5	5	4	4	3	3	3
5	4	5	5	4	4	4	4	3
3	3	3	4	3	3	3	3	3
4	4	2	4	3	2	4	4	1
3	3	3	3	3	3	3	3	3
5	5	3	3	3	3	3	3	1
4	4	4	4	2	2	3	3	1
4	5	4	4	3	3	4	4	1
5	5	5	4	1	2	3	3	3
4	5	3	4	1	1	5	5	1
5	5	3	3	3	3	2	2	2
5	5	5	5	3	3	3	4	1

5	4	4	3	3	3	2	4	1
5	5	5	5	5	5	5	4	3
4	4	1	4	3	4	4	4	1
3	4	4	3	4	4	4	4	3
5	5	4	5	3	3	2	3	2
4	4	2	3	2	2	2	3	1
4	4	4	4	3	3	5	4	2
4	4	4	4	3	2	3	3	3
5	5	5	5	4	4	1	5	5
5	5	5	5	4	5	3	4	4
4	4	4	4	3	3	3	3	3
5	5	5	5	5	4	4	4	2
5	4	5	5	4	4	5	3	1
4	4	4	4	3	3	5	3	3
4	4	4	5	3	4	3	3	2
3	4	4	4	4	5	5	5	3
4	4	4	4	2	2	2	4	3
4	4	4	4	4	4	2	4	4
5	5	4	5	2	2	4	4	4
4	4	4	3	5	5	5	5	1
3	4	3	3	4	4	3	3	3
4	4	4	4	3	3	3	3	3
4	4	4	4	3	3	4	2	4
5	5	5	5	1	4	3	3	3
5	5	5	5	3	3	3	4	3
4	5	4	5	4	4	4	3	2
5	5	4	4	4	4	4	4	3
4	4	5	4	3	3	5	4	3
4	4	4	4	1	1	4	4	3
4	4	4	4	3	3	3	4	2
5	5	5	5	4	4	3	5	4
5	5	5	5	5	5	5	3	1
4	4	4	4	3	3	3	2	3
5	5	4	4	3	3	3	4	2
4	4	3	4	2	2	5	4	1
5	5	3	4	2	3	3	3	2
5	5	5	5	5	4	4	2	3
5	5	4	5	3	4	3	4	3
5	5	3	5	1	3	4	4	3
5	5	4	5	1	2	3	4	1
4	4	4	4	4	4	4	3	2
5	5	5	5	2	3	3	3	4
4	5	4	5	2	2	5	2	2
4	2	4	4	3	4	4	4	1
5	5	5	5	4	4	4	4	3
5	5	5	5	2	3	2	3	3
4	5	4	4	5	5	5	4	1
4	5	4	2	5	5	5	4	2
5	5	1	5	5	5	5	2	1
4	4	4	4	3	4	4	3	2
5	5	5	5	4	3	3	4	4
4	4	5	5	3	5	3	3	3
5	5	5	5	5	5	5	5	5
4	4	4	4	4	3	5	3	1
4	4	3	4	5	3	4	4	3

4	4	4	4	4	4	4	4	2
5	5	5	5	5	5	3	5	5
4	5	3	3	2	2	4	2	1
4	3	5	5	5	4	3	4	1
4	4	2	4	5	5	5	5	2
5	5	5	5	2	3	3	3	3
4	4	3	4	3	4	5	3	3
4	4	3	2	3	3	5	3	1
5	5	5	4	2	5	3	5	1
5	5	5	4	3	5	3	5	1
3	2	3	3	3	3	3	3	3
4	5	3	4	1	1	3	3	1
4	5	4	4	3	4	3	3	3
5	4	3	4	2	3	2	2	1
5	4	4	4	4	4	5	5	1
5	4	4	4	4	4	5	5	2
3	5	5	3	3	3	4	2	3
5	5	5	5	5	5	5	5	5
4	4	4	4	5	5	5	3	3
4	4	4	4	3	3	3	4	3
5	5	5	5	5	5	5	3	3
4	4	4	4	3	3	3	3	3
5	5	5	5	3	3	3	4	3
4	4	3	5	5	5	5	5	1
5	5	3	4	3	3	3	3	2
4	4	2	4	1	1	4	4	4
5	5	5	5	5	5	5	5	4
5	5	5	5	1	1	4	4	1
3	3	3	4	2	2	5	4	4
5	5	5	5	5	5	5	5	3
5	4	4	5	5	5	5	4	1
3	5	4	3	1	2	5	5	2
3	4	3	3	1	1	4	3	4
4	5	4	4	2	3	5	4	3
5	5	5	5	3	3	3	2	1
4	4	4	3	3	2	3	3	1
4	5	5	4	2	2	2	2	2
4	4	4	4	3	3	3	4	2
5	4	4	4	4	4	5	5	1
5	5	4	4	4	4	4	5	2
5	5	5	4	2	3	3	3	5
5	5	5	5	5	5	5	5	5
5	5	2	5	1	4	4	4	1
5	5	5	5	1	1	1	1	3
5	5	3	5	4	3	4	4	1
3	5	2	3	1	1	4	1	1
4	4	4	4	2	3	5	3	1
4	4	4	4	4	4	4	4	3
5	5	4	5	5	5	5	5	1
5	5	5	5	5	5	5	5	1
4	4	3	4	5	5	4	4	2
3	3	4	3	5	4	4	5	1
4	4	3	3	4	4	5	4	1
3	3	3	3	3	3	4	3	2
4	4	4	4	2	3	3	5	3

5	5	5	5	5	5	5	4	1
4	5	5	4	3	4	3	4	3
3	3	2	3	2	3	3	3	2
4	4	4	3	2	3	3	4	1
3	3	3	3	3	3	3	2	3
5	5	5	5	3	3	3	4	3
5	5	5	5	5	5	5	5	5
5	5	5	5	5	5	5	5	3
3	3	3	3	4	4	3	4	1
5	5	5	5	3	3	3	3	3
2	2	2	2	2	2	2	2	1
5	5	5	4	2	2	2	2	4
3	5	4	4	1	1	4	1	2
5	5	3	5	3	3	3	4	3
4	4	4	4	3	3	3	3	3
5	5	5	4	4	3	3	3	1
4	4	4	4	3	3	3	3	4
5	5	5	4	5	5	5	5	3
4	4	3	4	4	4	5	3	3
4	4	3	4	2	2	3	2	1
4	5	5	3	5	3	4	4	1
5	5	5	4	5	3	4	2	2
5	5	5	5	4	4	4	3	3
4	5	5	5	4	4	4	1	3
3	4	1	4	1	1	5	4	1
4	5	5	5	4	4	3	4	1
4	4	4	4	2	2	5	5	4
4	4	4	4	2	2	2	3	3
4	4	3	4	2	3	5	2	2
4	4	4	4	4	4	3	4	2
3	3	2	3	2	3	4	3	2
5	5	5	5	3	3	5	1	5
4	4	4	4	5	5	5	5	3
3	3	3	4	4	3	3	4	3
4	4	4	4	2	3	3	3	3
5	5	5	4	2	2	4	4	3
4	4	4	4	3	3	3	4	3
4	5	4	5	5	3	5	5	4
4	4	4	5	5	4	5	1	1
3	1	4	3	4	3	4	4	1
4	5	5	4	5	5	2	5	5
4	4	4	3	2	2	3	4	3
2	4	5	5	4	4	4	5	1
4	4	4	4	3	3	3	2	2
4	3	3	3	2	2	3	2	1
4	4	3	2	1	1	5	5	2
4	4	3	4	5	4	4	4	2
4	4	4	4	3	2	3	3	2
4	4	4	4	2	2	2	3	2
4	5	3	4	4	5	3	4	1
4	4	4	4	2	2	3	4	1
5	4	4	4	2	1	5	3	1
5	5	5	5	3	3	3	4	3
4	4	4	4	4	2	2	2	1
4	4	4	4	3	3	4	3	2

4	3	5	1	5	5	5	5	2
4	4	3	3	2	3	3	3	2
4	4	4	4	4	4	2	2	3
4	4	3	4	3	3	3	3	1
5	4	4	4	3	3	4	4	3
5	5	5	5	1	2	5	1	1
4	5	2	4	1	2	5	3	4
3	3	2	4	3	3	5	2	1
4	4	4	4	3	4	4	4	3
3	5	3	3	3	2	3	4	3
4	4	3	4	1	1	4	2	1
5	5	5	5	2	2	2	2	2
4	4	4	4	2	2	4	3	3
4	5	4	4	2	3	2	4	1
4	4	4	4	5	5	3	3	3
4	4	4	4	2	2	2	5	2
5	5	4	5	2	3	3	4	1
3	2	3	3	2	2	4	2	1
4	4	3	4	2	2	3	4	1
4	4	4	4	1	1	4	3	1
5	5	5	5	3	3	5	5	2
4	3	4	4	3	2	4	4	1
4	4	4	4	2	2	2	4	2
3	3	3	3	3	3	3	3	3
5	5	5	5	5	5	4	5	4
4	4	3	4	1	1	1	4	1
4	3	4	4	2	2	5	4	2
3	3	3	3	4	4	4	4	2
5	5	5	5	3	4	3	4	1
4	4	4	4	5	5	5	5	4
5	5	5	5	5	5	5	1	5
3	4	5	3	5	4	4	4	3
4	5	4	5	5	5	5	5	3
4	4	4	4	3	3	5	5	2
4	4	4	4	2	2	3	3	1
5	4	4	4	3	4	2	2	2
3	5	3	5	5	5	3	3	1
4	4	4	4	5	5	5	5	1
5	4	4	5	3	5	3	3	1
5	5	5	5	1	1	1	1	2
4	4	4	4	2	2	2	2	2
4	5	4	4	3	3	4	4	4
4	4	3	2	1	2	4	4	3
5	5	5	5	3	3	3	3	2
4	4	4	5	5	5	5	5	1
5	5	5	5	2	2	3	3	3
4	4	4	4	2	4	4	4	2
4	4	3	3	2	2	4	4	2
5	5	4	4	3	3	3	3	3
5	5	4	5	2	2	3	3	3
3	4	4	4	2	2	2	2	1
4	4	5	4	3	3	5	5	2
5	5	5	5	5	5	5	5	3
5	5	5	4	5	4	5	5	3
5	5	4	5	1	1	1	1	3

5	5	5	4	5	5	4	4	3
4	4	4	4	2	3	4	4	2
5	4	5	4	2	2	3	3	3
4	4	4	4	2	2	2	2	1
5	5	5	5	4	4	4	4	1
3	3	3	3	3	3	3	3	2
5	5	5	3	4	4	4	4	3
4	4	4	3	2	3	3	3	1
4	4	4	4	1	1	4	4	2
4	3	3	4	2	1	4	4	2
4	4	4	4	3	3	3	3	4
4	4	3	4	2	2	2	2	2
4	4	4	5	2	2	3	3	2
2	3	4	2	1	1	5	5	1
4	4	4	4	2	2	2	2	1
4	4	4	3	3	3	3	3	3
3	3	3	4	3	4	5	5	1
5	5	3	5	5	5	3	3	2
3	4	4	3	4	4	4	4	2
4	4	4	4	3	2	2	2	1
3	2	2	3	2	2	5	5	2
5	5	4	4	3	4	3	3	3
5	4	4	3	3	3	3	3	2
4	4	4	4	4	4	3	3	3
4	4	2	5	5	5	5	5	1
5	5	5	5	2	3	4	4	5
5	5	5	5	4	4	4	4	4
4	4	5	5	2	3	3	3	2
4	4	4	4	5	5	4	4	3
5	5	5	5	5	5	5	5	5
5	5	5	5	5	5	5	5	4
5	5	4	4	2	3	3	3	3
2	5	3	3	3	3	3	3	3
4	5	4	4	1	1	1	1	1
4	3	4	3	4	4	4	4	3
2	4	3	2	2	2	2	2	1
5	5	4	3	5	5	5	5	5
2	3	2	3	5	4	4	4	1
5	4	4	4	2	2	4	4	2
3	4	4	3	5	5	5	5	2
3	3	3	3	3	3	3	3	2
5	5	3	5	5	5	5	5	5
4	4	4	4	2	2	3	3	3
5	5	5	5	5	4	3	3	1
4	4	4	3	4	4	4	4	2
4	4	3	3	2	2	3	3	3
4	5	4	5	5	3	3	3	1
5	5	5	5	5	5	5	5	5
4	4	4	4	4	4	5	5	3
3	4	3	3	1	1	4	4	1
4	3	3	4	5	5	4	4	3
5	5	4	1	5	4	5	5	1
5	5	5	5	3	4	4	4	2
4	4	4	4	4	4	4	4	2
4	5	4	4	3	4	5	5	3

5	5	5	5	1	1	1	1	3
4	4	4	5	2	3	3	3	2
3	3	3	3	2	2	4	4	1
4	4	4	4	2	2	2	2	4
4	3	3	3	3	2	4	4	1
4	4	4	4	3	2	5	5	2
5	4	5	5	4	5	3	3	3
3	3	3	3	4	3	4	4	2
4	5	4	4	1	3	2	2	3
4	4	4	4	2	2	2	2	1
5	5	5	5	5	5	4	4	5
4	5	4	4	3	3	3	3	2
4	3	2	3	1	1	4	4	1
3	3	3	3	3	3	3	3	3
5	5	5	5	5	5	5	5	3
4	4	4	4	3	3	4	4	3
5	5	5	4	3	3	3	3	2
5	5	5	5	3	3	3	3	2
5	5	5	5	5	5	5	5	5
5	4	4	3	5	5	3	3	1
5	5	5	5	4	5	5	5	3
4	4	1	3	1	1	5	5	1
4	4	1	3	2	2	4	4	1
4	4	4	4	3	4	3	3	3
5	3	5	5	1	1	5	5	1
4	5	4	4	4	4	3	3	5
5	5	4	5	3	4	5	5	3
4	4	3	4	3	3	5	5	2
3	3	4	3	3	3	5	5	1
4	4	4	4	3	3	4	4	1
3	4	4	3	3	4	4	4	1
4	5	5	4	2	2	4	4	3
3	4	4	4	3	2	5	5	1
5	5	2	3	2	1	3	3	3
5	5	4	5	3	2	2	2	3
4	5	3	4	1	2	5	5	1
5	5	5	5	4	4	1	1	5
3	5	4	4	1	2	3	3	2
5	5	4	4	3	3	5	5	3
5	5	5	4	2	2	2	2	1
4	4	4	4	2	2	2	2	4
4	3	3	4	5	5	4	4	1
5	5	4	4	4	4	4	4	4
4	4	4	4	4	4	3	3	3
4	5	4	4	3	3	3	3	1
4	4	5	4	5	4	4	4	3
1	4	2	3	4	4	4	4	4
4	4	4	4	2	4	4	4	1
3	4	2	2	1	1	1	1	2
4	5	5	4	3	3	4	4	2
2	5	4	4	4	4	4	4	2
4	4	4	4	3	3	3	3	3
4	4	3	3	1	2	2	2	3
4	4	4	4	5	5	5	5	1
5	5	4	4	2	2	4	4	1

4	4	4	5	2	3	2	2	1
4	4	4	4	5	5	3	3	2
4	4	4	4	3	3	4	4	3
3	4	4	3	4	3	5	5	1
4	5	4	5	2	2	3	3	5
4	4	5	5	2	2	1	1	2
4	4	3	3	2	2	3	3	3
3	4	4	4	3	4	4	4	2
4	4	4	4	3	3	3	3	2
5	5	5	5	5	5	5	5	5
5	5	5	5	1	1	1	1	3
5	5	5	4	3	3	3	3	2
4	4	4	4	3	4	3	3	3
5	5	5	5	1	4	5	5	3
4	4	4	4	2	4	4	4	4
3	3	3	4	4	4	4	4	5
4	3	3	4	3	3	3	3	2
4	4	2	4	4	4	3	3	1
4	5	3	4	3	2	5	5	3
3	5	4	4	3	4	3	3	3
4	3	2	4	4	4	4	4	1
5	4	4	4	2	2	3	3	1
5	5	5	5	4	4	3	3	1
4	4	4	5	5	3	5	5	3
4	4	4	4	4	4	3	3	2
3	4	4	4	4	4	3	3	2
4	4	4	4	3	3	3	3	2
4	5	4	4	3	5	3	3	1
5	5	4	5	2	5	5	5	4
4	4	4	4	4	4	4	4	4
5	4	3	4	2	2	2	2	4
5	5	5	5	3	3	4	4	2
3	3	4	5	3	3	3	3	2
4	5	3	3	2	2	2	2	1
5	5	4	5	1	3	4	4	1
3	3	3	3	2	2	2	2	3
3	4	4	4	4	4	4	4	3
3	3	2	3	4	3	5	5	4
4	4	4	4	3	4	4	4	1
4	3	4	4	4	4	5	5	3
4	5	4	5	5	5	5	5	4
5	5	4	4	3	4	5	5	2
4	5	4	4	2	3	4	4	1
5	5	5	5	5	5	5	5	5
5	5	5	5	4	4	4	4	3
5	5	5	5	2	3	4	4	3
4	4	4	4	3	3	5	5	1
5	5	3	3	1	3	3	3	1
4	4	4	4	2	3	1	1	2
5	5	5	5	5	5	4	4	5
5	5	5	5	4	4	3	3	3
4	4	2	3	3	3	5	5	3
5	4	5	4	5	4	3	3	1
5	5	4	5	5	4	4	4	3
4	4	2	3	1	1	3	3	1

2	4	3	4	3	3	4	4	1
3	3	3	3	2	2	4	4	2
3	4	3	3	3	3	3	3	2
4	4	4	4	3	3	4	4	2
5	5	5	5	1	3	3	3	2
3	4	3	4	2	2	4	4	2
4	4	4	4	2	2	4	4	1
4	4	4	4	2	3	3	3	3
4	4	4	4	4	4	4	4	1
4	4	4	4	4	4	2	2	2
5	5	5	5	1	1	1	1	1
5	5	5	4	4	4	3	3	2
4	4	4	5	5	5	3	3	3
3	4	4	4	5	4	3	3	2
5	5	5	4	5	5	3	5	4
5	5	5	5	4	4	5	5	4
5	5	4	4	4	4	4	4	2
4	3	4	4	4	4	3	4	2
5	5	5	3	3	3	3	3	3
5	5	5	5	4	4	4	3	3
5	5	5	5	1	5	5	5	3
3	4	4	4	4	4	3	3	2
4	5	5	5	4	4	4	4	2
4	4	3	4	5	3	5	4	1
5	5	5	5	5	5	5	1	3
5	5	5	5	5	5	5	5	5
4	5	5	4	3	3	4	4	3
5	4	4	5	5	5	4	2	1
5	5	4	4	4	4	5	2	4
5	5	4	5	4	4	4	4	3
4	4	4	3	1	3	2	3	2
4	4	3	3	3	2	5	4	1
3	4	4	4	4	4	4	4	3
4	5	5	3	5	5	5	5	1
4	4	4	4	4	5	5	4	3
4	4	4	4	3	3	4	4	1
3	3	2	2	3	3	4	4	2
5	5	5	5	3	3	3	5	1
4	4	4	3	4	4	4	3	3
5	5	5	5	4	4	3	5	2
4	4	3	3	2	2	4	2	2
4	4	5	5	2	2	4	3	3
5	5	5	4	2	3	3	5	2
5	5	3	5	3	3	4	4	2
5	5	5	5	3	3	3	3	2
4	5	5	4	4	4	5	5	2
5	4	4	4	2	2	3	2	1
3	3	2	3	2	2	3	4	1
5	5	3	4	4	4	4	4	3
5	5	5	5	1	1	4	4	3
4	4	3	5	3	3	3	2	3
3	5	3	2	1	1	5	1	2
2	2	2	2	4	4	4	4	2
5	5	4	5	5	4	5	5	1
3	5	4	3	4	4	4	3	2

4	4	4	4	5	4	4	4	1
5	5	5	5	1	5	2	3	4
4	4	4	4	2	2	2	2	4
5	3	3	5	3	4	4	4	4
3	3	3	4	4	4	4	4	3
4	4	2	2	2	3	4	5	1
4	4	3	4	4	3	4	4	1
4	4	4	2	4	4	4	4	4
5	5	5	4	3	4	3	4	3
5	5	5	4	4	4	5	4	1
4	4	4	4	3	3	4	4	2
5	5	5	4	5	5	3	5	3
5	4	4	4	4	4	3	5	1
5	5	5	5	5	5	4	4	3
4	4	4	4	3	2	3	3	2
2	3	3	4	2	3	3	3	1
4	5	1	4	2	1	5	5	5
4	4	4	4	3	3	4	4	3
3	4	2	2	1	3	2	4	2
4	4	4	4	2	2	4	3	3
5	5	5	3	4	5	5	4	1
3	3	3	3	1	2	5	1	1
5	5	5	5	1	1	1	5	3
5	5	5	4	3	4	3	4	2
5	5	4	5	3	2	3	4	1
3	5	4	4	5	4	4	4	3
5	5	4	4	5	5	5	5	1
4	4	4	4	4	5	3	5	1
4	4	2	4	4	3	3	4	3
4	5	4	5	4	4	3	4	3
3	3	3	3	2	2	5	3	1
4	4	4	4	4	4	4	4	3
4	3	4	4	4	2	4	4	1
5	5	5	5	3	4	3	5	3
4	5	5	4	5	5	5	5	4
5	5	5	5	5	5	5	4	2
5	5	4	4	2	3	3	3	2
5	5	5	5	2	3	4	4	4
5	5	3	5	1	1	5	4	2
4	4	4	4	5	5	2	5	1
4	4	5	4	5	5	4	4	2
5	5	5	5	3	3	5	3	1
4	4	4	4	3	4	4	4	2
5	5	5	5	5	5	5	5	3
4	4	4	4	2	2	4	5	2
4	4	4	3	3	3	3	4	1
4	5	4	4	4	2	4	3	1
3	3	4	3	2	2	2	2	1
5	5	4	5	1	1	1	5	1
1	3	3	3	1	1	1	1	1
4	4	4	4	3	4	4	4	2
3	3	3	4	2	3	3	3	1
1	2	2	2	4	1	5	1	1
4	4	5	3	4	4	5	3	2
5	5	5	5	1	1	1	2	2

4	4	3	4	2	2	3	5	2
4	5	2	5	1	2	4	1	1
3	3	2	3	3	3	3	3	2
2	3	2	3	4	4	4	3	1
5	5	2	5	1	1	4	2	3
2	3	3	2	2	3	5	3	3
5	3	2	4	2	2	5	2	1
5	5	5	1	5	5	5	5	1
4	5	4	5	5	5	3	2	3
5	5	4	5	5	4	3	4	2
5	5	5	5	5	5	5	1	3
3	4	2	4	2	2	2	2	3
3	5	3	4	2	2	3	4	1
5	5	5	5	1	1	3	4	2
3	3	3	3	3	3	4	3	2
2	3	3	3	3	2	4	4	1
5	5	5	5	1	1	1	5	5
5	5	5	5	5	5	5	1	5
3	3	3	3	3	3	5	1	2
5	5	4	3	1	2	3	2	3
5	4	4	4	1	2	2	4	3
3	4	4	3	3	3	3	3	3
4	5	4	4	1	1	5	2	1
4	4	2	4	2	2	4	2	1
5	5	5	5	3	4	3	4	1
4	5	5	4	1	3	5	4	1
4	5	5	5	5	5	3	4	2
4	4	4	4	2	2	2	2	4
4	5	5	5	2	3	2	5	2
4	4	4	4	4	4	4	4	3
4	3	3	4	3	2	3	4	3
4	4	4	4	4	4	3	4	2
3	2	3	3	2	3	3	3	3
5	5	4	4	4	4	4	5	3
5	5	5	5	4	4	4	4	5
4	3	4	4	3	4	3	3	2
5	5	5	5	5	5	3	5	3
5	5	5	5	4	4	4	4	5
4	4	3	4	5	5	5	5	4
5	5	5	5	2	2	2	4	2
4	2	3	3	3	4	4	4	2
3	4	3	3	4	3	4	2	3
4	3	2	5	2	3	2	3	4
5	5	5	4	5	3	4	5	3
5	5	4	4	2	2	2	2	1
4	4	4	3	4	4	3	4	2
2	3	3	3	3	3	3	4	2
4	3	3	2	5	5	5	5	1
3	2	1	3	4	4	5	4	1
3	3	3	3	3	3	3	3	3
5	5	5	5	4	4	5	4	1
5	5	4	5	5	5	4	4	1
3	3	3	4	4	4	5	2	3
4	5	2	4	5	4	4	4	1
4	4	4	4	2	4	3	5	3

5	5	5	5	1	1	5	4	2
1	1	1	1	5	5	5	5	1
5	5	5	5	1	3	3	3	5
5	5	5	5	5	5	5	4	4
5	4	5	4	5	5	4	4	2
2	4	2	3	2	2	4	2	4
5	5	4	4	5	5	4	5	3
5	5	5	5	4	4	4	4	5
3	3	3	4	3	3	3	3	1
4	5	5	4	5	5	5	5	3
4	4	4	4	3	4	3	5	1
5	5	4	5	5	3	5	5	5
5	5	4	4	4	4	4	5	1
5	5	5	5	5	5	4	4	2
5	5	4	5	5	5	5	5	4
4	4	4	4	2	3	4	4	2
5	5	5	5	4	4	4	3	5
4	4	3	4	1	2	4	3	2
5	5	5	5	5	5	3	5	5
4	4	4	4	3	3	3	5	3
5	5	5	5	5	5	5	5	5
3	4	2	3	3	4	5	4	1
5	5	5	5	3	3	3	3	3
4	4	3	4	5	4	4	5	3
4	4	4	4	3	3	5	4	3
4	5	4	4	5	5	5	1	2
4	4	4	5	3	4	3	3	1
3	5	3	2	3	2	5	3	4
5	5	5	5	3	5	5	5	1
4	4	4	3	3	3	5	2	1
1	1	1	1	5	5	5	5	1
4	5	5	5	2	5	4	5	3
5	4	4	4	4	4	4	4	4
4	5	4	5	3	3	4	3	3
5	4	3	4	3	3	4	4	3
4	4	4	4	2	2	2	5	2
4	4	4	4	3	2	2	4	1
4	5	4	3	4	4	4	4	1
4	3	4	4	2	1	2	5	1
4	4	3	4	4	4	4	4	3
4	5	5	4	1	2	3	3	2
4	4	5	4	3	4	4	3	1
4	4	4	4	5	5	5	3	3
4	4	4	4	4	5	3	5	2
5	5	4	5	5	5	5	5	2
3	5	4	4	4	3	4	4	3
4	4	4	4	4	4	4	4	2
3	3	4	2	5	5	5	3	1
4	4	4	4	3	3	3	3	3
3	4	4	4	3	3	4	4	2
4	4	4	4	4	5	5	5	3
3	3	4	3	2	2	5	5	1
5	5	5	5	4	4	4	4	1
5	5	5	5	5	5	5	5	4
4	5	4	3	5	5	5	5	3

5	5	5	5	5	5	5	5	3
4	4	3	3	3	3	3	3	2
3	3	1	4	5	5	5	5	1
5	5	5	5	5	5	5	5	5
4	5	3	3	4	3	3	3	1
3	5	5	3	5	5	3	3	1
3	5	3	3	4	4	4	4	2
4	4	4	5	4	4	4	4	2
5	5	3	5	1	1	2	2	4
5	5	5	5	5	5	5	5	3
4	4	4	4	5	5	4	4	3
4	4	4	4	4	5	5	5	2
4	4	4	5	2	3	5	5	1
4	4	4	4	3	3	3	3	3
5	5	5	5	2	4	3	3	3
3	3	3	3	4	3	4	4	1
4	4	4	4	5	5	4	4	1
5	4	2	4	2	2	2	2	1
3	4	3	3	3	3	4	4	3
5	5	5	5	5	5	4	4	1
3	5	5	4	5	5	5	5	3
4	4	4	4	2	2	2	2	2
5	5	5	5	5	5	5	5	4
4	4	3	3	2	3	4	4	1
4	4	4	4	5	5	3	3	1
4	4	4	4	4	4	4	4	2
5	5	3	4	3	3	3	3	1
5	5	5	5	4	4	4	4	3
4	3	3	5	3	4	3	3	2
5	4	3	5	2	5	5	5	2
5	5	5	5	1	1	1	1	5
3	3	3	3	3	3	3	3	1
2	4	3	2	2	3	5	5	1
4	4	4	4	3	3	4	4	2
4	4	4	3	5	5	5	5	3
4	4	2	4	2	2	5	5	2
3	4	4	4	3	3	3	3	3
4	4	4	4	2	2	2	2	4
3	5	4	4	5	3	4	4	2
4	4	3	3	4	4	4	4	2
5	5	5	5	1	1	1	1	5
3	3	3	3	3	3	3	3	2
3	5	3	4	5	5	5	5	3
5	4	4	4	5	4	5	5	1
3	4	3	3	1	1	5	5	1
3	4	3	3	2	3	5	5	1
4	5	4	4	4	4	5	5	2
4	5	4	5	5	3	4	4	3
3	3	3	4	3	4	4	4	1
4	4	5	4	3	4	3	3	1
5	5	5	5	2	1	1	1	1
4	4	4	5	2	3	3	3	3
5	5	5	5	5	5	5	5	3
4	5	3	4	4	4	3	3	1
5	5	5	5	5	5	2	2	4

5	5	5	5	1	2	3	3	5
4	3	4	4	4	4	4	4	2
4	4	4	4	4	4	4	4	4
5	5	5	5	2	2	2	2	4
5	3	2	4	2	3	5	5	1
4	4	4	4	3	3	4	4	1
2	2	3	2	2	3	5	5	1
3	4	4	3	5	5	3	3	3
3	3	3	3	3	3	3	3	3
5	5	5	5	5	5	5	5	3
3	4	3	3	2	2	3	3	3
5	5	5	4	5	5	5	5	3
5	5	5	5	4	4	3	3	1
5	5	5	5	1	5	5	5	1
4	4	4	4	2	3	3	3	2
4	4	5	5	3	4	4	4	3
5	5	5	5	5	5	5	5	4
4	4	4	4	4	4	4	4	3
4	5	3	5	4	4	3	3	4
4	4	4	3	4	4	4	4	3
5	3	5	5	1	2	5	5	1
5	5	5	5	5	5	5	5	5
3	4	4	4	3	3	5	5	3
4	4	4	5	3	3	3	3	3
4	4	4	4	2	2	2	2	1
5	5	3	4	4	4	4	4	4
4	4	4	4	4	4	5	5	3
3	2	2	3	2	2	3	3	2
4	4	4	4	3	3	3	3	2
3	3	4	3	2	2	3	3	4
4	5	5	5	4	5	2	2	4
5	4	4	4	1	2	3	3	3
5	5	5	5	3	4	5	5	5
5	5	4	5	5	5	3	3	3
4	4	3	3	4	4	4	4	2
4	3	3	4	2	3	3	3	3
3	4	4	3	3	4	5	5	2
5	5	5	5	1	1	4	4	3
5	5	5	1	5	5	5	5	2
2	4	3	2	2	2	5	5	2
3	3	2	3	4	3	3	3	1
5	5	4	4	4	4	3	3	2
4	5	4	4	2	3	4	4	3
3	4	4	3	3	4	5	5	1
5	4	4	4	2	2	5	5	1
5	5	5	5	4	4	4	4	3
4	4	3	3	5	5	4	4	1
4	5	4	4	3	3	4	4	2
5	5	5	5	5	5	5	5	5
5	5	4	4	4	3	3	3	3
4	4	5	5	2	2	2	2	2
3	4	4	3	4	3	3	3	3
5	5	5	5	2	2	3	3	3
3	4	4	3	4	4	5	5	1
4	4	4	4	3	3	2	2	1

5	5	4	3	5	5	5	5	1
5	5	4	3	5	5	3	3	4
5	5	4	5	5	4	4	4	5
5	5	5	5	1	5	5	5	3
4	5	3	2	4	5	4	4	3
4	5	5	4	4	4	4	4	1
4	3	4	4	3	3	3	3	2
4	4	3	4	2	2	2	2	2
5	5	5	4	4	4	3	3	3
4	4	4	4	3	3	3	3	1
3	5	4	3	2	3	4	4	1
4	5	4	4	3	3	4	4	4
4	4	4	4	2	2	2	2	4
4	4	4	3	5	5	5	5	1
5	5	5	5	5	5	3	3	5
5	5	5	5	5	5	5	5	3
3	3	3	3	3	3	4	4	2
5	5	5	5	2	3	4	4	3
5	5	4	5	5	4	4	4	1
4	4	3	3	4	2	5	5	1
4	4	4	4	5	4	5	5	1
5	5	4	4	3	4	4	4	5
5	5	5	5	3	3	3	3	2
3	4	4	3	3	2	3	3	3
4	4	4	3	4	4	4	4	3
3	3	2	3	2	2	5	5	1
5	5	3	4	3	3	3	3	3
3	3	2	4	4	4	5	5	2
4	4	4	4	5	5	5	5	1
4	4	5	4	2	4	4	4	2
5	5	4	5	2	3	4	4	2
5	5	5	5	3	3	5	5	2
4	5	4	3	1	4	3	3	4
4	4	3	4	2	1	3	3	2
4	4	3	4	3	4	3	3	1
4	4	4	4	5	2	2	2	2
5	5	5	4	5	3	4	4	5
4	4	4	2	5	4	4	4	3
5	5	5	5	5	4	3	3	3
4	4	4	3	3	3	4	4	2
5	5	5	2	4	4	4	4	4
4	5	3	4	3	3	3	3	1
3	4	2	3	3	3	3	3	2
4	5	5	5	3	3	3	3	2
3	4	2	3	5	4	5	5	3
4	4	4	4	5	5	5	5	1
4	3	3	3	3	3	3	3	3
3	3	2	3	1	1	3	3	2
3	4	4	4	2	2	2	2	1
4	4	4	4	2	4	4	4	2
4	4	4	4	4	5	3	3	2
4	4	3	3	4	3	4	4	1
5	5	5	5	3	3	3	3	2
4	4	3	4	2	3	3	3	3
4	4	4	4	2	3	3	3	3

4	4	3	4	3	4	4	4	3
4	4	3	4	3	4	3	3	3
3	4	5	4	1	3	5	5	1
4	4	4	4	4	4	3	3	4
5	5	5	5	5	3	5	5	4
4	4	3	5	5	4	4	4	1
3	4	4	4	4	4	5	5	1
5	5	5	5	4	5	5	5	4
4	5	4	4	4	4	5	5	3
3	5	2	3	2	2	4	4	1
5	5	3	5	1	4	4	4	5
5	3	3	4	5	5	5	5	1
4	5	4	3	3	3	4	4	2
4	4	5	4	2	3	4	4	4
5	5	5	5	1	1	1	1	1
4	4	4	4	5	5	5	5	1
5	5	4	5	1	2	4	4	2
5	5	5	5	5	5	5	5	4
5	5	5	5	3	3	4	4	2
5	5	5	5	3	3	3	3	1
4	3	3	4	2	2	3	3	2
4	4	4	4	5	5	5	5	1
4	4	4	4	5	3	3	3	3
4	3	2	4	3	3	5	5	1
5	5	5	5	1	2	2	2	2
5	5	5	5	2	3	3	3	2
3	5	4	4	3	3	5	5	1
5	5	5	5	4	4	3	3	3
3	2	4	2	5	4	5	5	3
4	4	3	4	3	3	4	4	3
5	5	4	4	5	5	5	5	4
4	4	3	4	3	4	4	4	2
5	5	5	5	4	4	4	4	5
4	4	4	4	5	5	5	5	1
4	4	4	4	4	4	3	3	3
4	4	4	3	4	4	3	3	2
4	4	4	3	5	4	3	3	3
4	3	3	3	5	5	5	5	3
3	4	4	3	4	4	4	4	2
4	4	4	4	3	4	5	5	4
3	3	3	3	3	3	3	3	3
3	4	4	4	2	3	3	3	2
4	4	4	4	5	5	5	5	2
5	4	4	4	1	2	4	4	4
5	5	3	4	5	5	4	4	3
5	5	5	5	3	3	3	3	3
4	5	5	5	1	3	4	4	5
5	5	5	5	4	4	1	1	5
5	5	5	4	5	5	5	5	3

JR11 (1=Never;2 =Seldom;3= Sometimes;	JR12 (1=Never;2 =Seldom;3= Sometimes;	JR13 (1=Never;2 =Seldom;3= Sometimes;	JR14 (1=Never;2 =Seldom;3= Sometimes;	JR15 (1=Never;2 =Seldom;3= Sometimes;	W_FC1 (1=Strongly disagree;2= Disagree;3=	W_FC2 (1=Strongly disagree;2= Disagree;3=	W_FC3 (1=Strongly disagree;2= Disagree;3=	W_FC4 (1=Strongly disagree;2= Disagree;3=
1	1	1	2	1	5	5	5	5
3	2	2	3	3	3	3	3	3
1	1	4	2	5	5	5	5	5
1	1	2	3	2	4	5	5	5
1	1	3	4	2	5	4	5	4
3	4	4	5	5	4	4	4	4
2	1	1	3	3	3	5	4	3
3	3	3	3	3	3	3	3	3
2	2	2	2	2	4	4	3	3
4	4	5	5	5	3	3	3	1
2	2	3	3	3	4	4	4	4
3	3	5	4	4	2	3	3	3
2	1	3	3	3	5	4	4	3
3	3	3	3	3	3	3	3	3
3	4	3	3	3	4	3	3	3
1	3	4	3	3	4	4	4	4
1	1	3	4	4	4	3	3	3
3	4	3	4	4	2	2	2	2
5	4	5	5	4	3	2	3	3
3	4	4	3	3	3	3	3	3
2	3	4	4	4	4	4	4	4
2	1	1	5	5	5	5	5	5
3	4	4	3	3	3	4	3	4
4	3	4	4	4	2	3	3	3
2	2	3	3	3	3	3	3	4
1	3	2	3	1	4	4	5	3
3	4	3	4	4	3	4	4	3
3	3	4	4	4	2	3	2	1
3	3	3	3	3	3	3	3	3
1	2	3	3	3	4	3	4	4
2	2	3	3	3	4	4	4	4
1	2	3	3	3	4	4	4	4
2	2	2	3	2	4	4	4	4
1	1	2	3	3	5	5	5	5
2	4	4	4	4	4	4	4	3
5	4	5	1	1	3	3	2	3
2	2	2	3	3	5	5	5	5
4	4	5	5	5	3	3	3	3
2	3	3	3	3	4	3	4	3
2	4	2	3	3	3	3	3	3
2	4	4	4	4	2	3	2	2
1	1	1	1	1	5	5	5	5
3	3	3	3	3	3	3	3	3
3	3	3	3	3	4	4	3	4
3	4	4	3	3	4	3	3	3
1	3	2	1	1	3	4	3	3
1	2	4	4	4	4	4	4	4
2	2	3	4	4	3	3	3	3
2	2	4	3	3	2	3	3	3
3	2	2	2	3	4	3	4	2
1	1	5	5	5	4	2	4	2

3	3	3	3	3	5	4	4	5
1	1	3	3	3	3	4	4	3
3	2	3	3	2	3	4	4	4
3	1	1	1	1	4	4	4	4
1	2	4	4	3	4	3	3	3
3	2	3	3	3	4	3	3	3
3	3	4	3	3	3	3	4	2
2	2	3	3	3	3	3	3	3
3	3	3	3	3	2	2	3	3
1	2	1	2	2	5	5	5	5
1	1	3	2	2	4	4	5	5
3	3	4	3	3	3	3	3	3
4	4	4	4	4	4	3	3	4
3	2	2	2	2	4	4	4	4
3	2	3	2	4	3	4	4	4
3	3	4	3	3	3	3	4	3
3	4	3	4	3	4	4	4	4
1	2	2	1	1	5	4	4	4
1	1	2	2	2	3	3	3	3
4	3	5	4	3	3	3	3	3
2	1	4	3	3	4	3	3	4
1	1	4	4	3	5	5	5	5
4	4	3	4	3	3	3	3	3
1	1	1	2	2	5	4	4	4
3	1	4	2	2	5	5	5	5
1	1	1	1	1	5	5	5	5
5	5	3	3	3	2	2	2	2
2	2	2	2	2	4	4	4	3
1	1	2	1	3	5	5	5	5
1	1	2	2	2	4	4	4	4
2	3	3	3	2	4	4	4	4
3	3	4	2	4	2	2	2	2
1	2	2	2	2	4	4	4	4
5	4	4	4	4	3	3	3	2
3	3	3	2	3	4	3	3	4
5	5	5	5	5	1	1	1	1
3	2	3	3	2	3	4	3	4
2	2	3	3	3	4	3	3	3
3	4	3	3	3	2	1	2	2
2	2	3	5	3	5	5	5	5
3	3	4	5	5	3	3	3	3
1	1	1	1	1	5	5	5	5
5	5	5	5	5	1	2	2	3
3	3	4	3	3	3	3	3	3
1	2	2	2	3	4	4	4	4
1	1	1	2	2	5	4	5	5
5	5	5	5	5	4	4	4	3
3	3	3	3	3	3	2	2	3
2	3	2	3	3	4	4	4	3
3	3	3	2	2	3	3	3	4
1	2	3	3	3	4	4	3	4
1	1	2	3	3	5	4	4	4
1	1	1	3	3	3	3	3	4
4	4	4	5	5	3	2	2	2
4	2	3	1	2	4	4	4	4

3	3	3	3	3	4	4	4	4
3	2	3	3	3	3	3	4	4
3	2	3	3	3	4	5	3	4
4	3	3	2	2	5	5	5	5
1	1	1	2	3	5	5	5	5
1	1	5	1	1	1	1	1	3
3	1	1	2	2	3	4	4	3
1	1	1	2	2	5	5	5	5
3	2	2	2	1	5	5	5	5
3	3	3	3	3	3	3	3	3
3	4	3	3	3	3	4	4	4
1	2	2	2	2	3	4	4	3
3	3	3	3	3	4	3	3	4
1	1	2	2	2	4	3	3	4
1	3	5	4	4	4	5	5	4
1	2	2	3	2	4	4	4	5
2	2	3	3	3	4	3	3	3
2	3	2	3	3	4	5	4	4
4	3	3	1	3	4	4	4	4
1	4	2	3	2	4	3	3	4
2	2	3	3	2	3	4	4	4
5	4	4	4	4	3	4	3	3
2	1	1	5	5	3	3	3	3
2	2	2	2	2	4	4	3	4
2	2	2	3	3	4	4	4	3
4	4	5	4	4	3	3	2	3
2	2	4	5	5	2	2	3	2
5	4	3	2	2	5	5	5	5
1	1	3	3	2	2	3	3	3
4	5	5	5	5	2	2	4	3
1	2	3	3	3	3	3	3	3
2	1	1	2	2	3	4	4	4
2	2	1	2	1	4	4	4	5
1	1	1	1	1	4	4	5	5
4	3	3	2	2	2	3	3	3
1	1	1	1	1	5	5	5	5
2	4	3	2	2	3	4	4	4
2	3	1	3	1	4	4	4	4
1	1	1	2	1	2	3	3	3
2	2	4	4	4	5	4	5	4
1	1	3	2	2	5	5	5	5
3	3	3	3	3	4	3	3	4
4	2	4	4	4	5	5	5	5
4	5	4	5	5	3	3	2	3
2	3	3	3	3	3	4	4	3
1	1	1	2	2	5	5	5	5
3	3	3	3	3	2	3	3	2
1	1	1	2	2	3	5	4	4
2	4	4	4	4	4	4	4	5
5	5	5	4	4	1	1	1	1
3	3	4	3	3	3	3	3	3
3	5	5	5	5	1	1	1	1
2	2	2	2	2	4	3	4	4
3	1	2	3	2	5	5	5	5
3	3	3	3	3	3	3	3	3

1	1	1	1	1	5	5	5	5
3	3	4	2	2	4	4	4	4
4	2	4	4	4	4	4	4	4
4	4	5	5	5	3	3	3	3
3	3	4	5	5	5	3	2	3
2	3	3	3	3	3	3	3	3
2	2	2	3	2	4	4	4	4
3	3	2	2	2	4	3	4	3
1	1	1	1	1	5	5	5	5
3	3	4	4	5	3	3	3	3
2	3	5	4	4	2	3	2	2
2	2	4	4	4	3	5	4	3
3	3	3	3	3	3	3	3	2
1	1	3	2	2	3	3	5	5
2	2	2	2	1	3	3	2	2
3	3	3	2	2	4	3	3	4
4	4	3	3	3	3	3	4	4
1	1	1	5	5	5	5	5	5
1	1	1	1	1	5	5	5	5
4	4	4	3	1	4	2	2	2
3	4	3	3	3	3	3	3	3
4	4	4	3	3	2	3	2	2
1	2	3	5	5	4	5	5	5
3	4	5	4	4	3	3	3	3
1	3	3	4	4	3	4	3	2
3	3	5	5	4	3	4	5	3
1	3	5	5	3	5	5	3	3
2	5	4	4	4	3	3	3	3
5	5	5	5	5	2	4	2	2
1	2	3	5	5	4	3	3	3
1	3	2	3	3	3	2	2	3
4	4	4	4	4	3	3	2	4
1	1	3	3	2	4	4	4	5
3	3	3	3	3	3	4	3	4
2	4	5	5	5	3	3	3	3
3	4	5	5	5	3	3	3	3
1	1	1	3	5	5	5	5	5
3	4	5	5	5	4	3	3	3
2	3	3	2	2	2	2	3	3
3	3	3	2	3	3	3	3	3
5	5	5	4	4	1	2	2	3
2	2	3	3	3	3	4	4	4
2	3	3	2	2	3	3	3	4
3	2	4	3	2	3	3	3	3
3	3	3	4	4	3	3	3	3
1	3	3	3	3	4	5	4	4
2	2	3	3	3	3	3	4	4
2	3	3	3	3	2	2	2	3
1	2	2	2	2	3	4	4	3
3	2	4	5	4	3	3	3	2
3	3	2	2	2	4	4	3	3
3	3	3	3	3	4	4	4	4
2	2	4	4	4	4	3	3	4
1	3	3	3	3	3	4	4	4
2	2	2	3	2	4	3	3	3

4	2	3	2	3	4	5	5	5
2	3	5	5	5	3	3	3	3
1	1	1	1	1	4	5	5	5
3	2	2	2	2	4	4	4	4
1	1	2	2	2	5	5	5	5
2	2	4	4	4	2	1	1	1
1	1	2	2	2	2	5	5	4
1	1	1	1	1	3	5	5	5
2	3	2	2	2	2	2	2	2
5	5	5	5	5	2	2	2	2
3	3	3	3	3	3	3	3	3
2	2	2	2	2	4	4	4	4
1	3	2	2	2	3	3	2	3
3	3	3	3	3	3	3	3	3
1	1	2	3	2	4	3	4	4
2	2	2	3	2	5	5	5	5
3	3	4	5	4	3	3	4	4
1	1	1	1	1	4	4	5	5
3	3	2	2	2	3	3	3	4
2	3	2	3	2	5	5	5	5
5	3	2	2	2	4	4	3	4
3	4	5	5	5	2	4	3	2
1	1	3	3	3	5	5	5	5
2	2	4	4	4	3	3	3	3
2	2	2	3	2	3	4	4	4
1	1	5	1	5	5	5	5	5
2	3	2	2	2	3	3	3	3
3	3	3	3	3	3	3	3	4
3	3	2	3	2	5	4	4	4
3	3	3	3	3	3	3	3	3
2	4	4	4	4	1	1	1	1
3	4	2	2	2	3	3	4	3
1	2	4	4	4	3	4	4	3
1	3	3	3	3	3	3	3	3
1	1	2	2	2	5	5	5	5
4	4	4	4	4	2	2	2	2
3	3	2	3	2	4	4	3	4
3	3	3	3	3	3	3	3	3
2	3	3	3	3	3	3	3	3
3	3	5	5	5	3	4	4	4
1	1	1	3	1	4	3	2	4
3	2	3	3	3	3	3	4	5
3	2	3	4	3	3	3	3	2
3	4	3	4	3	3	4	4	4
5	5	5	5	5	2	2	2	2
3	2	5	5	5	2	2	4	2
3	3	4	4	4	3	4	4	4
3	2	3	2	3	5	5	5	5
1	1	2	2	2	5	3	3	3
3	4	5	4	5	2	3	3	3
3	3	5	4	5	2	2	2	2
3	3	3	3	3	3	3	3	3
4	4	5	5	5	3	3	2	3
3	3	4	4	4	3	4	4	3
3	3	4	4	4	4	3	3	3

2	2	1	2	1	4	3	4	3
3	2	3	3	3	5	5	5	4
2	2	3	3	3	3	3	3	3
2	3	2	2	2	4	4	4	3
1	1	3	4	3	5	5	5	5
2	3	2	2	2	4	3	4	5
5	3	2	2	2	2	2	1	1
3	3	4	4	4	4	3	3	3
3	3	5	5	5	2	2	2	2
2	2	3	1	3	3	3	3	4
3	3	2	2	2	4	4	3	4
3	4	4	4	4	2	2	2	3
4	3	3	2	3	4	3	3	3
3	2	3	3	3	4	4	3	4
1	1	3	3	3	5	5	5	5
2	2	2	2	2	3	3	3	3
3	1	3	3	3	3	3	3	2
3	3	3	3	3	3	4	4	4
3	3	3	3	3	3	3	3	3
4	1	1	1	2	1	4	4	4
3	3	4	4	4	3	3	3	3
4	5	5	5	5	2	2	2	2
3	4	4	3	3	3	2	1	1
5	5	5	4	4	1	1	1	1
4	4	3	3	3	3	3	2	3
3	3	3	3	3	3	3	3	2
3	3	3	3	3	4	4	4	4
4	3	3	5	5	4	3	3	4
3	3	4	4	4	4	4	4	4
1	1	3	2	3	3	2	2	3
4	4	5	4	5	2	2	2	1
3	3	3	3	3	3	5	3	3
3	5	5	4	4	1	2	2	2
2	2	2	2	2	3	3	4	5
3	3	3	5	5	3	3	3	2
3	2	3	3	3	3	3	3	3
2	2	2	3	3	3	4	3	3
1	2	1	3	2	1	3	2	1
4	3	3	4	4	2	2	2	3
2	3	4	4	4	1	4	2	3
4	4	5	5	5	3	3	3	3
3	4	4	4	4	3	3	3	3
2	2	2	3	3	4	4	4	4
1	1	3	3	3	3	2	2	2
3	3	3	3	3	3	3	3	4
1	1	1	5	5	5	5	5	5
2	2	2	5	2	5	5	5	5
3	3	5	5	5	3	1	1	1
2	3	3	4	4	4	4	4	5
4	2	5	3	3	3	3	3	3
2	2	5	4	4	3	2	4	3
3	2	2	1	1	3	5	5	4
2	1	2	2	2	4	4	5	4
5	5	5	3	4	1	3	2	1
2	2	3	4	3	3	4	4	3

2	2	2	3	3	3	3	3	3
2	3	3	3	3	3	4	3	3
2	3	4	4	4	2	4	2	2
1	1	3	3	3	3	4	3	4
3	3	3	3	3	3	4	2	4
4	2	3	3	2	2	5	3	3
3	3	4	3	3	3	3	3	2
1	1	1	2	3	5	5	5	5
2	2	2	3	3	5	5	5	4
3	3	4	4	4	3	3	4	3
3	3	3	4	4	3	4	3	3
2	3	4	4	4	3	3	3	3
1	1	3	3	3	3	3	3	3
2	3	3	3	3	2	2	2	3
1	4	4	5	5	3	4	3	3
1	1	1	2	2	4	4	4	5
5	5	5	5	5	1	1	1	1
1	1	1	2	2	5	5	5	5
3	4	4	2	5	3	3	2	2
3	3	3	4	3	3	3	3	2
3	2	4	3	2	3	4	4	4
4	4	5	5	4	2	2	2	2
4	5	5	5	5	1	1	1	1
4	5	4	5	1	3	3	3	3
1	1	1	2	2	3	4	5	5
3	2	4	4	4	3	3	3	4
4	5	5	1	1	1	1	1	1
3	2	4	4	4	3	4	4	4
4	4	4	2	2	3	3	3	3
3	3	5	5	5	2	5	4	2
5	5	5	5	5	1	1	1	1
5	5	5	5	5	1	1	1	1
4	4	4	2	2	3	3	3	3
2	3	3	3	3	3	4	4	4
3	2	3	3	2	3	4	4	4
3	3	4	4	3	3	4	4	3
5	5	5	5	5	1	1	1	1
3	4	5	3	4	3	4	4	4
4	3	4	5	5	3	5	5	5
2	1	3	3	3	4	5	5	5
5	2	4	3	3	2	2	2	3
3	4	4	4	4	4	3	3	3
3	4	5	5	5	2	2	2	2
2	2	3	3	3	2	3	3	3
3	3	3	3	3	3	3	3	3
2	3	4	4	4	4	4	5	4
3	2	3	3	3	3	4	4	3
2	1	3	2	2	2	3	3	4
2	2	1	2	2	5	4	5	5
4	3	3	4	4	3	4	4	4
2	3	3	3	2	3	3	4	3
3	3	2	2	2	3	3	3	3
1	1	5	5	5	5	5	5	5
2	2	3	3	3	3	3	3	3
4	3	2	2	2	3	4	3	3

3	3	4	4	4	2	5	5	4
3	3	5	5	5	3	3	3	3
3	2	2	2	2	4	4	4	4
3	3	2	2	2	4	3	2	2
2	2	2	2	2	3	4	4	4
1	1	3	3	3	4	5	5	4
3	1	3	3	3	3	4	4	4
2	2	5	5	5	4	4	4	4
2	3	3	3	3	4	3	3	3
2	2	3	3	3	5	5	4	4
2	2	4	4	4	4	4	4	4
3	1	2	2	2	5	5	5	5
1	1	1	1	2	5	5	5	5
3	4	5	5	5	1	3	1	1
3	4	3	3	4	3	2	2	1
2	2	4	4	2	4	3	3	4
4	3	5	5	5	2	1	1	3
1	3	4	4	5	5	5	5	5
2	2	3	3	3	4	4	4	4
2	3	4	4	4	3	3	3	3
4	4	3	3	3	3	3	3	3
3	2	3	3	3	3	4	4	3
2	4	4	4	2	3	3	3	2
5	4	1	1	2	5	5	5	5
3	3	5	5	5	3	1	2	2
1	1	3	3	3	4	4	4	5
2	2	2	2	3	4	4	4	4
4	1	5	5	5	1	3	3	5
3	3	3	3	3	3	3	3	3
3	4	4	4	3	3	3	3	3
2	2	2	2	2	4	4	4	4
3	2	2	2	1	4	4	4	4
3	4	4	4	4	3	3	4	4
3	4	5	5	5	5	5	5	5
3	3	4	4	4	4	4	4	4
3	3	3	3	3	3	3	3	4
3	2	3	3	3	5	3	3	3
3	4	5	5	5	3	2	3	2
2	2	3	3	3	2	2	2	2
1	1	3	3	3	3	3	3	2
2	1	3	3	3	4	3	3	3
2	3	3	3	3	4	3	5	5
3	2	3	3	2	4	4	4	3
3	3	5	5	5	1	3	1	2
5	5	5	5	5	4	3	3	4
3	3	3	3	3	3	3	3	3
1	1	4	4	4	3	5	5	5
5	5	5	5	3	3	3	5	2
2	2	4	4	4	4	4	4	4
2	2	3	3	2	4	5	5	5
3	4	3	3	3	3	3	3	3
1	1	4	4	4	4	5	4	5
5	4	4	4	4	2	2	2	2
2	2	3	3	4	4	4	3	4
1	1	2	2	2	4	4	4	4

2	2	4	4	4	4	5	5	4
1	4	5	5	5	4	4	4	4
3	3	3	3	3	4	4	3	4
1	1	2	2	2	4	3	4	4
4	3	4	4	3	2	4	4	4
1	1	1	1	1	4	4	4	2
3	4	3	3	2	3	3	3	3
3	3	3	3	3	2	2	2	2
3	3	2	2	2	4	4	4	4
1	1	3	3	2	4	4	4	4
2	2	5	5	4	2	3	3	3
2	3	2	2	3	4	4	4	4
3	2	3	3	4	3	4	3	3
1	2	4	4	5	3	3	3	2
3	4	3	3	3	2	3	3	2
5	5	3	3	3	1	1	2	2
2	4	3	3	3	5	4	4	4
3	3	4	4	4	4	4	4	3
4	4	4	4	3	3	3	3	3
4	1	3	3	3	4	3	3	4
2	2	2	2	2	5	4	4	3
3	3	3	3	3	3	4	4	3
2	2	2	2	2	3	3	3	4
3	4	3	3	3	4	4	4	4
1	1	4	4	4	4	4	3	4
1	2	3	3	3	4	4	4	3
1	3	4	4	4	3	5	5	5
3	3	3	3	3	5	5	5	5
2	3	3	3	3	3	4	4	4
3	3	3	3	3	3	2	2	2
1	1	1	2	2	5	5	4	5
4	3	4	2	3	4	4	4	4
2	1	4	3	3	3	3	3	3
4	3	2	2	2	3	1	4	5
1	1	3	1	1	4	4	4	4
2	2	2	2	2	3	5	4	4
4	3	4	3	3	4	4	3	4
3	2	3	3	3	3	3	3	3
4	3	3	3	3	3	3	3	3
2	2	2	2	2	4	4	4	5
3	3	3	3	3	3	3	3	3
3	3	3	3	4	3	2	3	3
4	4	3	3	2	3	3	3	3
2	2	2	2	2	4	4	4	3
1	1	1	1	1	4	4	4	4
3	3	4	3	3	2	3	3	3
3	2	3	5	4	5	3	3	4
3	3	3	3	3	3	4	5	5
1	1	1	3	2	5	4	4	4
3	3	3	4	4	3	2	2	2
3	3	3	3	3	3	3	3	3
3	3	3	3	3	5	5	5	5
4	5	5	5	5	3	3	3	2
1	1	2	2	2	4	5	5	4
3	3	4	3	4	4	4	4	4

2	3	2	2	2	3	3	3	3
2	3	2	2	2	4	4	4	4
3	3	4	2	2	4	4	4	3
5	5	5	5	5	1	1	1	1
1	2	3	3	3	4	5	3	3
3	3	5	5	5	1	4	4	4
1	3	3	3	3	2	3	3	3
1	1	1	2	2	4	5	5	5
2	2	2	2	2	3	3	3	3
3	4	4	3	5	2	3	2	1
3	3	5	5	5	1	1	1	1
1	1	2	3	3	5	4	4	5
2	2	3	4	4	3	3	3	4
4	2	1	2	2	4	4	3	5
3	4	3	3	3	2	3	3	2
4	3	4	2	2	4	4	4	4
3	3	4	4	4	2	2	2	3
2	3	3	4	4	3	3	3	3
2	2	4	3	2	3	4	3	3
4	2	5	3	3	2	2	2	2
3	2	3	5	5	1	4	3	3
1	2	3	3	3	3	5	4	4
2	2	2	2	2	5	5	5	5
3	4	3	3	3	1	3	2	2
1	3	4	4	4	3	3	3	3
2	3	3	3	3	3	3	4	4
4	3	4	3	3	2	2	2	2
2	3	3	3	3	4	4	3	3
3	1	2	4	4	3	3	3	3
3	3	3	3	3	4	4	3	4
2	4	5	4	4	3	2	2	1
4	3	5	1	2	3	4	2	2
3	2	4	4	4	3	3	2	3
3	2	3	3	2	5	4	3	4
3	2	3	3	3	2	3	3	3
5	5	5	4	4	1	1	1	1
3	3	3	4	4	3	4	3	3
2	2	3	3	2	3	4	3	3
2	3	4	3	3	3	3	3	3
3	3	1	3	2	3	2	2	2
3	3	3	3	2	4	4	4	4
2	2	3	2	2	3	4	4	4
1	2	2	2	2	5	4	4	3
3	3	3	3	3	4	4	4	4
2	2	3	3	3	4	4	4	4
4	4	5	5	5	1	1	1	1
3	3	3	3	2	4	4	3	3
4	4	4	2	2	2	2	3	2
4	1	2	2	2	5	5	5	5
3	5	4	4	4	3	2	3	3
2	2	3	3	4	3	4	3	4
3	4	3	4	2	2	2	2	2
2	3	4	3	3	3	3	3	3
2	2	2	2	2	5	5	5	5
3	3	3	3	3	5	3	3	5

1	3	2	2	2	5	5	5	5
1	1	2	2	2	4	5	5	5
4	4	2	3	2	4	3	4	2
3	4	4	4	4	3	4	3	3
2	2	3	3	3	3	3	3	3
4	5	2	2	2	2	1	2	1
2	2	3	3	3	3	3	4	4
2	1	1	2	1	5	5	5	5
3	3	3	3	3	3	3	3	3
1	5	5	5	5	2	2	2	1
2	2	2	2	2	4	3	4	4
4	3	3	3	3	3	2	2	3
3	3	5	4	5	2	2	3	3
4	3	4	4	4	3	3	3	3
1	1	3	3	3	4	4	5	5
3	4	3	3	3	3	3	3	3
3	3	2	4	2	2	2	2	2
3	3	4	4	4	4	3	5	4
1	1	2	1	2	5	4	4	5
2	3	2	2	2	3	3	2	4
3	3	3	3	3	3	3	3	3
1	3	3	2	3	3	3	3	3
4	5	5	5	5	2	2	2	2
1	1	1	1	1	3	3	4	4
2	1	2	3	2	3	4	4	4
1	1	2	2	2	3	5	4	4
3	2	5	5	5	3	3	3	4
2	2	2	2	2	4	3	4	4
1	3	3	3	3	5	5	5	5
2	5	5	5	5	1	1	1	1
3	3	3	3	3	3	3	3	3
3	3	3	3	3	4	5	5	4
2	2	3	3	3	4	5	5	5
3	4	5	5	5	2	2	2	2
1	2	2	3	2	3	4	3	3
1	1	1	1	1	3	5	5	5
1	1	2	2	2	3	4	3	2
2	2	1	1	1	5	5	5	5
2	3	2	3	2	4	3	3	4
3	3	3	3	3	4	3	3	4
2	2	4	2	4	3	3	4	4
1	1	4	4	4	3	5	5	5
3	2	3	3	3	4	3	3	3
4	4	5	5	5	1	1	1	1
4	4	4	4	4	3	3	2	3
3	3	3	3	3	4	4	4	4
1	1	1	2	1	5	5	4	5
3	3	3	3	3	3	3	3	3
3	3	4	4	4	3	3	3	3
1	2	2	2	2	4	4	4	5
3	2	3	3	3	4	4	4	4
3	3	2	2	2	1	3	3	3
1	1	2	3	2	5	5	4	4
1	1	3	3	3	3	3	3	4
2	3	4	4	4	3	3	2	2

4	3	3	4	3	3	3	3	3
3	4	4	4	4	3	2	2	2
3	3	3	3	3	4	3	3	5
3	3	4	4	4	3	2	2	3
3	3	3	3	3	3	5	5	5
1	1	2	3	2	4	5	5	5
2	2	3	3	3	3	3	3	3
3	3	3	3	3	3	3	3	3
5	5	5	5	5	1	1	1	1
5	5	2	2	2	1	1	1	1
3	3	4	4	4	3	3	3	3
1	2	3	3	3	3	4	4	3
4	3	4	4	4	4	2	2	2
2	2	3	4	3	2	2	3	2
3	4	5	4	5	3	3	3	3
5	5	4	4	4	3	1	1	2
3	3	3	3	3	3	4	3	3
4	4	4	2	4	3	3	3	3
3	3	4	2	2	5	5	5	5
2	1	1	1	1	5	5	5	5
3	3	4	3	3	3	4	3	3
3	3	4	4	4	3	3	3	2
3	4	4	3	3	2	2	2	3
3	3	5	5	5	1	1	1	1
3	4	3	2	3	2	2	2	2
3	3	3	3	3	3	3	3	3
4	4	3	3	2	3	3	3	3
3	3	3	3	3	3	3	3	3
2	3	2	1	1	3	4	4	5
3	3	3	3	3	4	4	4	4
4	5	4	3	3	2	2	2	2
1	1	1	2	2	4	4	4	4
4	3	4	3	3	3	3	3	3
2	3	3	3	3	3	4	4	3
2	1	3	1	1	5	5	3	5
3	2	2	3	3	4	4	4	4
3	4	3	5	4	2	3	3	3
4	4	4	4	4	2	3	2	2
3	2	3	5	5	3	4	4	3
2	4	4	5	5	2	2	2	2
2	2	3	3	3	3	3	3	3
4	4	5	4	4	2	2	4	1
1	1	4	3	4	4	4	3	3
3	2	3	3	3	4	4	4	4
3	4	4	4	4	2	2	2	2
3	3	3	4	3	2	3	3	2
1	3	3	4	3	2	3	3	2
2	2	3	3	3	3	3	3	3
1	1	1	2	2	4	5	5	5
2	2	3	3	3	4	4	4	4
4	5	5	5	5	1	1	1	1
3	2	3	3	3	3	3	3	3
5	5	5	5	5	1	1	1	1
2	3	5	1	1	5	4	4	4
3	4	3	4	3	2	4	4	4

2	2	2	3	3	4	4	4	4
5	5	5	5	5	1	1	1	1
1	1	3	3	3	4	3	3	4
1	1	3	2	2	3	3	3	4
3	3	4	5	5	4	4	4	4
3	4	5	5	5	3	3	1	1
3	3	4	4	4	3	3	4	4
1	2	2	3	3	5	4	3	4
4	2	4	2	2	2	2	2	2
2	2	4	3	3	5	5	5	5
3	3	3	3	3	3	3	3	3
1	2	2	2	2	4	4	4	5
3	3	2	2	3	3	2	3	4
1	1	1	2	1	5	5	5	5
2	3	3	3	3	3	3	3	3
2	3	3	3	3	3	3	3	3
3	3	3	4	3	3	4	4	3
5	5	5	5	5	1	1	1	1
3	3	3	3	3	3	3	3	3
2	3	4	3	4	3	3	2	2
3	3	4	3	3	3	2	3	3
3	4	3	4	4	2	4	4	3
3	3	3	3	3	2	3	3	2
1	1	2	2	1	3	2	2	3
3	3	4	3	3	2	3	3	4
3	2	2	2	2	3	3	3	2
4	4	4	3	3	1	1	1	1
1	1	1	1	1	1	3	3	4
4	1	3	3	3	4	4	4	4
2	5	1	5	5	4	4	4	4
3	4	4	3	3	2	3	3	4
2	3	1	3	3	4	4	4	4
1	1	3	3	3	3	3	3	3
3	4	4	3	2	3	4	3	3
1	1	1	5	5	5	5	5	5
1	1	2	4	4	4	5	5	5
2	3	2	1	1	4	4	4	4
3	3	4	4	4	3	2	2	3
2	3	3	3	3	3	4	3	3
3	4	4	3	3	3	3	2	2
4	5	4	3	4	2	2	2	2
5	5	5	5	5	2	1	1	1
2	2	5	5	5	5	4	3	4
3	3	4	3	4	5	5	5	5
3	1	3	3	3	5	4	4	4
1	1	3	3	3	5	5	5	5
1	1	1	3	3	2	3	2	2
4	3	4	4	4	4	4	4	4
1	3	5	5	5	3	4	4	4
2	5	5	3	3	3	3	3	3
1	2	3	3	3	4	3	3	3
1	4	3	3	3	3	3	3	3
1	1	1	1	1	4	4	4	5
2	3	3	3	3	4	5	4	4
4	2	3	3	4	2	2	2	2

4	2	3	4	4	3	4	4	3
2	3	4	4	4	4	4	3	3
1	1	3	3	3	4	3	4	5
2	2	2	4	4	4	4	4	4
4	2	4	4	4	3	3	3	2
2	3	5	4	4	2	2	2	1
1	2	3	3	2	4	5	5	5
4	3	5	5	5	1	3	1	2
1	1	3	3	3	4	3	3	3
4	4	4	5	5	2	2	2	2
1	1	1	2	2	5	5	5	5
4	4	4	2	2	3	3	3	3
4	2	3	2	2	3	4	3	3
3	2	3	2	3	4	4	4	4
3	4	3	3	3	2	2	2	2
1	2	5	3	3	5	5	5	5
3	2	3	3	3	3	3	3	3
3	3	4	5	5	3	3	3	3
4	4	4	4	3	3	2	3	2
1	2	2	2	2	5	4	4	4
3	3	4	4	4	3	2	2	2
2	2	3	2	2	4	4	3	2
4	4	4	4	4	3	2	3	3
3	2	4	4	3	4	4	4	4
1	1	1	1	1	3	4	4	5
2	3	4	2	2	3	3	3	2
1	3	4	3	3	3	3	3	3
2	2	3	3	3	4	4	4	4
2	2	3	3	3	4	4	4	3
3	4	4	4	4	3	3	3	3
2	2	2	2	2	4	5	5	5
3	3	5	5	5	1	1	1	1
2	3	4	4	4	5	4	4	4
2	2	2	2	3	4	4	4	4
3	3	4	4	3	4	4	4	3
3	3	3	3	3	3	2	2	2
2	2	2	3	3	4	5	5	5
3	3	4	4	4	3	3	3	2
1	1	1	2	2	5	5	5	5
1	2	2	3	2	3	5	4	4
4	3	5	4	4	1	2	3	3
4	3	3	4	4	2	3	3	4
1	1	2	4	3	5	5	4	4
2	2	4	3	2	3	3	3	3
1	1	1	2	4	4	4	4	4
1	1	2	2	2	3	5	4	4
3	2	3	3	3	2	2	3	4
3	3	3	3	3	4	3	4	4
2	1	3	2	3	4	4	5	5
1	2	4	3	3	3	5	5	5
1	1	1	2	2	5	5	5	5
2	1	3	4	5	4	3	3	5
4	4	3	4	4	3	3	3	4
1	1	1	2	2	3	3	3	3
3	3	4	3	3	3	3	3	3

3	4	1	5	5	4	4	4	4
1	1	2	3	2	3	4	4	3
3	3	3	2	2	3	2	3	2
1	1	2	2	2	5	4	4	5
3	4	3	3	3	3	3	3	4
1	1	4	5	5	5	5	5	5
1	2	5	4	4	4	4	5	5
1	1	1	3	3	4	5	5	5
3	3	3	3	3	3	3	3	3
3	3	4	4	4	5	5	4	4
1	1	1	3	2	4	4	3	3
2	2	3	3	3	3	3	3	3
3	3	2	2	3	3	4	4	4
3	2	3	2	2	3	4	3	4
3	4	4	5	5	2	2	2	2
2	2	3	3	3	3	4	3	2
1	1	1	3	3	2	3	3	3
1	1	3	4	2	3	4	3	3
2	4	4	3	3	3	2	2	3
2	1	3	2	2	4	4	4	4
3	3	2	4	4	4	4	3	3
2	1	3	3	3	4	3	3	3
1	2	3	2	2	3	5	5	5
3	3	3	3	3	3	3	3	3
4	4	5	5	5	2	3	3	2
1	1	1	2	1	5	5	5	5
2	2	3	2	2	3	3	2	2
2	2	3	3	3	3	3	3	3
4	4	4	3	3	3	3	3	3
3	3	3	3	3	3	3	3	3
5	1	5	5	5	1	1	1	1
5	5	5	4	4	3	3	3	3
4	3	5	5	5	4	2	2	2
3	3	3	3	3	4	3	3	3
1	1	2	4	3	4	4	4	3
2	3	2	2	2	4	3	4	4
2	4	1	3	5	3	5	5	5
1	3	3	3	3	3	3	3	3
2	1	3	3	3	2	3	3	4
1	1	1	3	3	5	4	4	4
4	3	4	3	3	3	3	3	3
4	4	4	3	3	3	2	2	2
3	3	3	3	3	4	4	4	4
5	2	3	1	1	3	3	4	4
1	1	5	5	5	4	3	3	3
3	4	4	3	4	2	3	4	2
2	2	3	3	5	4	4	4	3
4	2	4	4	4	4	3	3	3
3	3	3	3	3	3	4	3	2
3	3	5	1	3	3	2	2	2
1	1	1	2	2	5	5	5	5
4	3	4	3	3	3	3	3	3
3	4	5	5	5	1	1	1	1
2	3	3	3	2	3	3	3	3
3	4	4	4	4	1	1	1	1

3	4	4	3	3	4	4	4	3
2	2	2	2	2	4	4	3	4
2	2	2	3	2	4	4	4	4
4	1	4	3	3	4	4	4	4
1	1	1	1	1	1	5	5	4
2	2	2	3	2	5	5	5	5
4	3	3	4	3	3	3	3	3
2	3	2	3	3	3	4	4	3
4	2	2	1	1	5	5	5	5
2	2	4	4	4	3	5	5	3
4	4	3	3	3	4	3	3	3
2	2	2	3	3	4	3	4	4
2	3	4	2	2	3	2	2	2
2	2	2	3	3	4	5	5	5
1	1	2	3	4	4	4	3	3
3	3	3	3	3	4	4	4	4
2	2	3	3	3	3	4	4	4
5	5	5	4	3	1	1	1	1
2	3	3	3	3	2	2	2	2
1	1	1	2	3	3	4	4	4
4	3	3	2	3	5	5	5	5
3	4	3	3	3	2	4	3	2
2	3	4	4	4	3	4	4	4
3	3	2	2	2	4	4	4	4
1	1	1	4	3	5	5	5	4
2	3	3	4	4	2	3	3	2
4	4	5	4	4	3	3	2	3
2	3	3	3	3	3	2	2	3
3	4	4	3	3	2	2	2	2
5	5	5	5	5	2	2	2	2
5	5	5	5	5	1	1	1	1
3	3	4	3	3	3	3	3	3
3	3	3	3	3	3	4	4	4
3	1	3	2	3	4	5	5	4
4	3	4	4	1	4	4	4	4
3	2	3	3	3	4	4	4	4
5	4	5	5	5	3	3	1	3
1	1	2	1	1	5	5	5	3
2	2	3	3	3	4	5	4	5
1	2	3	5	4	4	5	5	3
2	2	2	3	2	4	3	4	4
5	5	5	5	5	2	2	2	3
1	3	3	3	3	3	5	5	5
5	5	3	3	2	3	4	4	3
2	2	3	3	3	3	3	3	3
3	2	4	2	2	4	3	3	4
3	2	4	5	4	3	4	2	1
5	5	5	5	5	2	2	2	3
3	4	4	3	3	2	2	3	2
2	1	1	2	2	5	5	4	5
3	3	3	4	4	5	4	5	5
1	1	1	2	2	5	5	5	5
2	3	5	4	4	3	2	2	2
1	2	4	4	4	4	4	4	4
1	3	4	3	3	3	4	4	2

4	4	4	4	4	1	1	1	1
3	3	3	3	3	3	3	4	4
1	3	2	3	3	4	4	4	4
4	4	4	3	2	2	2	2	2
2	3	2	2	2	4	3	3	3
1	3	3	3	3	4	4	5	4
3	4	3	4	4	4	3	3	2
3	2	3	3	3	4	3	3	3
3	4	3	3	2	4	2	3	2
1	3	4	3	3	3	3	3	3
4	5	4	5	4	2	2	2	2
1	3	3	4	4	3	2	2	2
1	1	1	2	2	5	5	5	5
3	3	3	3	3	5	5	5	5
1	1	3	4	4	4	3	3	2
3	3	3	4	4	5	3	1	1
2	2	3	2	2	4	4	4	4
4	2	2	3	2	4	3	5	5
5	5	5	5	5	1	1	1	1
2	3	3	5	4	4	4	4	3
3	3	4	4	4	3	3	3	3
1	1	3	1	1	5	5	5	5
1	1	3	2	2	3	5	5	5
3	3	3	5	4	3	3	3	3
1	1	3	3	3	5	3	3	3
4	4	4	5	5	4	3	2	2
4	4	3	3	3	2	3	3	2
1	2	2	3	2	4	4	4	4
1	1	2	2	3	4	5	4	3
1	1	3	3	4	3	4	4	3
1	1	1	2	2	4	4	4	4
4	3	3	2	3	3	3	4	4
1	1	1	3	5	5	5	5	5
3	2	4	4	4	5	5	5	5
3	3	3	2	3	4	4	4	4
1	3	4	2	2	3	3	3	3
5	5	5	4	4	1	1	1	1
1	1	2	1	5	5	5	5	5
4	3	3	4	4	3	3	3	3
3	4	2	3	3	3	2	3	3
4	3	3	3	3	4	3	3	3
1	3	3	2	2	3	2	2	4
3	3	4	4	4	4	4	4	4
3	3	4	4	4	3	3	3	3
2	3	3	5	4	1	4	3	5
2	3	4	3	2	2	4	4	4
3	2	3	2	2	4	4	4	4
1	1	1	2	2	3	4	4	4
2	2	3	2	2	4	4	3	3
2	2	3	2	4	4	4	4	4
3	3	4	3	3	3	4	3	3
3	3	4	3	3	2	4	4	3
3	3	4	2	2	4	3	3	4
1	3	5	5	5	1	3	3	3
2	3	3	4	2	3	3	3	3

3	2	4	2	2	4	5	4	5
3	4	3	3	3	3	3	3	3
3	3	3	3	3	3	3	3	3
1	3	2	3	3	5	4	4	5
4	3	4	5	4	2	3	3	3
1	2	5	2	2	2	2	2	2
1	1	3	3	2	3	4	4	3
3	2	3	3	3	4	4	4	4
3	3	3	3	3	4	4	4	4
5	5	5	5	5	1	1	1	1
3	3	3	3	3	3	3	3	3
4	4	5	4	4	2	2	2	2
3	3	4	4	3	3	3	3	3
3	3	5	4	4	3	4	2	2
4	4	3	3	3	4	4	4	4
3	4	4	2	2	4	4	4	5
3	3	3	2	2	2	3	2	2
1	1	4	5	5	4	5	4	2
2	3	4	4	4	3	3	3	3
3	3	4	5	5	3	3	3	3
3	3	3	3	3	4	3	3	3
1	1	2	2	2	4	4	5	4
3	2	3	3	3	3	5	4	4
3	3	3	5	5	4	3	3	4
3	2	3	3	3	4	4	5	4
3	3	4	2	2	3	4	3	2
2	2	4	2	2	4	3	3	3
3	4	4	4	4	3	4	3	2
2	2	4	4	4	2	2	2	1
4	4	3	5	5	2	2	2	2
4	2	3	3	3	4	3	3	3
2	3	3	4	3	3	4	3	3
2	2	2	2	2	4	4	4	4
3	3	2	2	2	3	5	5	4
2	2	2	3	2	3	4	4	4
1	3	3	3	2	3	3	3	3
3	3	3	3	3	3	4	4	4
1	4	4	3	3	2	4	4	5
3	3	3	3	3	4	4	4	5
2	3	4	3	4	4	4	4	3
2	3	4	4	4	2	3	3	2
2	3	3	3	3	4	2	2	3
1	2	4	2	2	4	5	4	3
5	5	5	5	5	1	1	1	1
2	3	3	2	2	3	4	4	4
2	3	5	5	4	1	1	3	2
3	1	3	3	2	4	3	3	3
1	1	2	2	2	5	5	5	5
2	2	2	2	2	5	5	5	5
5	5	5	5	5	3	2	2	2
3	3	4	3	3	4	4	4	4
2	3	3	3	3	3	3	3	3
4	3	3	3	2	3	4	3	4
3	3	4	3	3	3	4	3	2
1	1	3	1	1	5	5	5	5

1	1	1	1	2	5	5	5	5
3	2	2	2	2	3	3	3	3
3	3	3	2	2	2	4	3	3
2	2	2	3	4	4	4	4	4
5	3	5	3	3	3	4	3	3
2	2	2	3	3	4	3	3	4
1	1	1	2	2	5	5	5	5
2	2	3	2	2	2	2	3	2
2	3	4	2	3	3	4	3	4
2	3	3	3	3	4	4	4	5
1	2	3	3	3	3	3	4	4
3	3	3	4	3	3	4	4	3
4	4	5	5	5	1	3	2	3
3	3	3	3	2	5	4	3	4
2	3	5	5	5	1	3	1	2
4	4	4	5	5	3	4	4	4
3	1	2	4	4	4	4	4	4
3	3	3	3	3	4	3	3	3
3	3	2	3	1	3	2	3	3
3	4	4	4	4	3	3	3	3
2	4	4	4	4	3	3	3	2
3	3	3	2	2	4	3	3	4
3	4	5	5	5	2	3	3	2
1	1	1	2	2	5	5	5	5
4	3	5	5	5	2	3	3	2
1	5	5	5	5	1	3	3	4
1	3	3	4	4	2	2	2	3
3	3	4	4	4	4	4	3	3
4	4	3	3	3	2	2	2	1
3	2	3	3	3	2	3	3	3
2	2	3	3	3	4	4	3	4
1	1	1	2	2	4	5	5	4
3	2	3	2	2	3	4	3	4
1	2	4	4	4	2	4	4	4
4	2	3	3	2	4	4	4	3
3	1	4	2	2	3	3	3	4
3	2	3	4	3	4	4	4	4
4	3	3	5	5	3	3	3	3
2	3	3	4	4	4	3	3	3
3	4	5	5	5	1	1	1	1
2	2	3	3	3	3	2	4	4
3	4	4	4	4	3	2	3	2
2	2	3	4	3	3	3	4	3
2	2	3	3	3	2	4	3	3
2	2	5	4	4	2	2	3	1
2	3	3	2	2	4	4	3	5
1	3	3	3	3	2	3	3	2
2	2	2	2	2	4	4	4	4
3	3	3	3	3	3	4	3	2
3	4	3	2	2	4	3	4	4
3	3	3	4	3	3	3	4	3
1	2	3	2	2	4	4	4	5
2	2	2	4	4	5	5	5	5
2	3	2	3	2	5	3	2	5
3	3	3	3	3	3	3	3	4

1	1	2	4	3	5	5	5	5
2	5	5	5	5	1	1	1	1
4	4	3	4	3	3	3	3	3
3	3	4	3	4	4	2	2	2
2	3	2	3	3	3	3	3	2
1	1	2	3	2	5	5	5	5
2	4	3	3	3	2	4	4	2
4	5	3	4	4	3	2	2	2
4	3	4	4	3	3	3	3	3
1	1	5	5	5	2	2	4	4
2	2	3	2	2	3	2	2	1
5	5	5	5	5	1	1	1	1
1	2	1	2	2	2	3	3	3
2	2	2	2	3	3	5	4	3
2	2	4	4	3	2	3	3	2
1	1	2	3	3	4	4	4	4
1	1	3	1	1	5	5	5	5
3	3	4	4	4	2	4	3	3
1	1	4	2	2	5	5	5	5
4	4	4	5	5	2	2	2	2
1	1	3	3	3	2	3	3	3
1	2	1	1	1	5	5	5	5
3	1	3	3	3	4	5	5	4
3	3	3	3	3	3	3	3	3
2	1	1	2	2	5	5	5	5
1	2	3	4	4	3	3	4	2
4	3	3	5	5	4	3	3	3
3	3	2	2	2	4	3	3	4
4	3	3	2	3	3	3	3	2
4	3	4	4	4	4	3	2	3
2	1	2	2	3	5	5	5	5
3	3	3	3	3	2	2	2	2
1	1	1	1	1	5	5	5	5
3	3	5	5	5	2	2	2	2
4	5	5	4	5	2	2	1	2
2	4	5	3	3	3	1	2	3
1	2	4	5	4	3	4	4	3
4	4	4	4	3	2	2	2	2
2	2	2	3	2	5	5	4	4
1	3	4	4	3	3	2	2	2
4	2	2	3	3	4	4	4	3
1	2	4	3	3	3	4	3	3
1	2	3	1	2	5	4	4	4
4	4	4	5	5	2	2	2	2
2	2	3	3	3	4	4	4	3
1	1	1	3	3	5	5	5	5
3	3	3	2	3	4	4	4	3
1	1	1	2	2	5	5	5	5
3	2	3	4	4	4	4	4	3
1	1	3	3	3	3	4	4	4
3	2	3	4	3	5	4	4	4
1	1	2	3	2	4	4	4	4
1	1	1	1	1	5	5	5	5
3	4	4	5	4	2	2	2	4
4	3	5	1	1	1	1	1	1

2	3	3	2	2	3	4	4	4
4	4	3	5	5	4	3	3	3
2	1	2	2	2	4	4	4	5
1	1	2	2	2	5	4	5	4
3	3	4	2	2	2	2	2	2
1	3	4	3	3	5	5	5	5
1	1	1	5	5	5	5	5	5
4	2	5	5	5	3	3	3	2
2	4	5	1	1	2	2	2	2
2	3	2	3	3	2	3	4	2
3	2	5	5	4	3	4	4	4
3	3	3	2	2	3	3	3	3
1	1	2	2	2	4	5	5	4
2	3	4	4	4	4	4	4	4
2	1	3	2	2	3	3	3	3
1	2	1	2	2	5	5	5	5
2	5	5	5	5	3	3	3	3
5	5	5	5	5	1	1	1	1
2	5	4	2	2	3	4	3	5
4	4	3	3	2	4	3	3	4
4	4	4	2	2	3	3	2	3
3	3	3	3	3	3	3	3	3
1	1	2	2	2	5	3	3	5
1	2	3	3	2	4	4	4	4
2	2	4	4	4	2	3	3	2
1	1	1	2	2	3	4	3	3
3	2	5	5	5	2	4	3	3
4	4	2	2	3	3	3	3	3
2	2	3	3	3	3	3	3	4
3	3	4	3	3	2	4	2	2
3	4	3	4	4	3	2	2	2
2	3	3	3	3	4	3	3	4
2	3	4	4	3	3	3	3	3
3	4	4	3	3	3	3	3	3
5	5	5	4	4	1	1	1	1
3	3	3	3	3	3	3	3	3
3	5	5	5	5	2	2	2	2
5	2	4	4	4	3	2	2	2
4	3	3	3	3	3	2	2	2
3	3	2	3	4	3	3	3	4
2	2	3	3	3	3	3	4	4
2	2	3	2	2	4	4	3	3
3	4	5	4	4	3	3	3	3
2	3	4	5	4	3	4	3	4
1	1	1	5	4	4	4	4	4
2	3	4	4	4	2	2	2	2
2	2	3	2	2	4	4	4	4
1	1	1	3	2	5	5	5	5
2	3	3	3	3	4	4	4	4
2	2	3	2	3	3	3	3	3
3	3	3	3	3	4	4	4	4
1	1	1	1	1	5	5	5	5
3	3	2	2	2	4	5	5	4
4	2	1	1	1	5	5	5	5
3	3	4	3	3	4	4	4	4

1	3	3	4	4	3	1	2	1
1	1	1	1	1	5	4	5	5
5	5	5	5	5	1	1	1	1
2	3	3	3	2	3	4	3	3
4	3	4	3	4	5	4	4	4
2	2	2	2	2	4	4	4	4
3	1	3	5	5	2	3	2	2
5	5	5	4	4	2	2	3	3
1	2	3	3	3	4	4	4	4
3	3	5	4	4	1	1	1	1
1	1	4	2	2	2	2	2	2
5	5	5	4	4	1	1	2	1
2	1	5	1	1	2	3	3	3
3	3	3	3	3	2	2	2	2
1	3	5	5	5	5	3	4	1
2	1	3	2	3	4	4	5	5
5	5	5	4	4	1	1	1	1
1	2	4	3	3	3	3	4	3
5	5	5	5	5	1	1	1	1
4	3	4	4	4	3	3	2	2
3	5	5	5	5	1	1	1	1
1	1	1	2	2	3	4	4	3
3	5	5	3	3	3	3	3	3
3	3	4	4	5	5	5	5	3
4	3	3	3	3	2	3	3	4
5	3	1	5	5	4	4	3	5
3	2	2	2	2	5	3	3	4
4	3	3	3	3	3	4	4	4
3	3	5	5	5	2	4	2	1
2	1	2	2	2	5	5	5	5
1	1	1	5	5	3	3	3	3
1	4	5	5	5	3	3	3	1
4	3	3	4	3	3	4	4	4
1	3	4	2	2	2	2	2	2
4	3	3	3	3	4	2	2	3
2	1	3	3	3	3	3	3	3
2	3	1	2	2	4	5	5	5
1	2	4	1	5	4	5	4	3
1	1	1	2	2	5	5	5	5
4	3	3	4	4	3	4	4	4
2	3	3	3	3	2	3	3	2
1	1	3	2	2	4	4	4	4
3	4	4	3	3	4	3	3	4
2	3	3	3	4	4	3	4	5
5	3	2	2	2	3	4	5	4
3	3	4	2	2	3	3	3	4
1	3	3	3	3	5	3	3	3
1	1	3	5	5	4	3	3	5
3	4	4	3	3	3	2	2	2
2	2	2	2	2	3	4	3	4
2	4	3	4	4	4	3	3	3
4	4	4	4	4	2	5	4	4
1	1	5	1	1	5	5	5	5
3	4	5	5	5	1	1	1	1
4	3	5	5	5	4	4	4	1

2	4	5	5	5	3	3	3	3
2	1	2	3	3	3	4	4	4
3	1	2	2	2	5	5	5	5
5	5	5	5	5	1	1	1	1
1	1	2	2	3	5	5	5	5
3	5	3	2	1	4	4	4	1
2	2	3	3	3	4	4	4	4
2	2	1	4	4	4	4	4	4
5	5	5	5	5	2	2	2	2
3	5	5	5	5	2	4	1	1
3	5	5	5	5	4	4	4	3
3	3	4	3	3	3	2	2	2
2	3	3	5	5	3	5	5	5
2	3	3	3	3	3	3	3	3
3	4	4	3	3	3	2	2	2
1	2	2	1	1	3	5	5	4
1	1	3	2	2	5	5	5	3
2	1	2	2	2	4	4	4	4
2	2	3	2	2	4	5	5	5
2	4	5	5	5	2	3	3	3
1	2	3	3	3	4	4	4	5
2	2	2	2	2	4	4	4	4
5	5	4	5	5	1	1	2	3
1	1	3	3	3	5	5	5	5
1	1	2	2	2	2	4	3	3
2	2	3	3	3	4	4	4	4
1	2	2	2	2	5	5	5	5
4	4	4	4	4	2	3	2	2
3	3	2	2	2	2	4	4	4
2	3	5	5	5	4	5	3	3
5	5	5	5	5	1	1	1	1
1	1	5	1	5	5	5	5	5
1	2	4	3	3	5	3	3	5
3	2	3	3	3	4	4	3	3
3	3	3	3	3	2	3	4	3
1	2	4	4	4	3	3	3	3
4	3	2	2	2	3	4	4	3
4	4	4	4	4	2	3	3	3
3	3	3	3	3	4	3	3	4
2	3	4	3	3	2	3	4	3
1	5	5	1	1	1	1	1	1
3	3	3	3	3	4	3	3	4
4	3	3	3	3	5	4	4	3
1	2	4	2	2	4	4	4	4
1	1	1	1	1	4	3	3	3
4	2	3	3	3	4	5	4	5
1	3	3	2	2	4	3	3	3
3	2	5	5	5	3	3	3	2
2	1	1	1	1	5	5	5	5
1	2	3	3	3	5	4	5	5
3	3	2	2	2	4	5	5	5
3	4	4	4	4	2	2	2	2
3	3	4	3	3	3	3	3	3
1	1	5	4	4	3	5	4	3
4	5	5	5	5	1	1	1	1

4	5	5	4	4	2	2	1	1
3	3	3	3	3	4	4	4	4
3	3	3	3	3	4	3	3	3
4	5	5	3	2	1	1	1	1
2	1	4	3	3	3	4	3	3
2	1	1	2	1	3	4	4	5
1	1	1	3	3	5	4	4	4
3	3	3	2	3	3	3	3	3
2	3	2	2	4	4	4	4	4
4	3	3	4	4	3	4	4	3
1	1	4	4	4	3	4	3	3
3	3	4	2	1	2	3	3	3
1	1	2	4	4	3	4	4	4
1	1	1	2	1	5	5	5	5
2	1	3	3	3	5	5	5	5
3	4	3	3	3	3	4	4	4
4	5	5	5	4	3	3	3	3
3	4	4	4	4	3	3	3	2
3	3	5	4	4	2	2	2	3
3	3	4	3	4	4	4	4	4
1	1	1	5	5	5	5	5	5
1	2	4	5	5	5	5	5	3
3	4	4	4	3	5	5	5	5
4	5	5	5	5	2	1	1	2
1	1	2	3	3	5	5	5	5
3	4	4	4	4	2	2	2	2
3	3	3	4	4	4	3	2	3
3	2	2	2	2	4	4	4	4
2	2	4	4	4	4	4	4	4
3	5	3	2	2	3	3	4	2
3	3	4	3	3	4	4	3	4
3	4	4	4	4	4	3	3	3
5	5	5	5	5	1	1	1	1
4	5	5	5	5	2	2	3	2
2	2	3	3	3	4	4	4	4
1	3	2	3	2	3	4	4	4
3	3	4	5	5	2	3	2	2
2	2	2	3	4	3	3	3	4
1	1	3	3	3	3	3	3	3
1	1	1	5	1	3	3	3	5
1	1	1	3	3	5	5	5	5
2	3	4	3	3	3	3	3	3
4	3	2	2	3	3	4	4	3
1	1	2	2	2	3	2	3	4
4	1	3	3	2	4	3	3	4
4	4	4	3	3	3	3	3	3
2	2	2	2	3	4	3	4	4
2	3	5	5	5	1	3	3	2
5	3	5	5	5	3	3	3	3
2	3	3	3	3	2	3	3	3
2	2	2	3	3	3	3	3	3
3	3	3	3	3	3	3	3	3
2	3	2	2	2	3	3	3	2
1	1	1	2	2	4	4	5	5
4	3	2	2	2	4	4	3	3

3	4	4	4	2	3	3	3	4
3	3	5	5	5	3	3	3	3
4	3	5	4	4	1	1	1	4
4	4	5	5	5	1	5	5	3
3	2	1	3	2	4	4	4	4
1	2	5	3	3	4	3	3	5
2	2	3	1	1	3	3	4	4
1	2	2	2	2	4	5	5	4
4	5	5	5	5	3	2	2	3
1	3	2	2	2	3	2	3	2
3	3	2	3	3	4	4	3	4
2	2	3	2	1	2	2	3	2
4	4	3	3	3	4	4	4	4
2	2	3	5	5	3	5	4	4
5	5	5	5	5	1	1	1	1
5	5	5	5	5	3	2	3	2
1	2	2	3	4	4	5	5	5
4	3	3	3	4	2	2	3	2
3	3	4	4	2	3	3	3	3
1	1	1	2	2	4	4	4	4
1	1	2	2	2	5	5	5	5
4	4	4	4	4	3	2	2	2
3	2	3	3	3	3	4	2	3
2	2	2	2	2	3	4	4	4
3	3	4	4	4	4	4	3	4
1	1	1	1	1	5	5	5	5
3	3	3	3	3	4	4	3	2
2	3	4	2	4	5	5	5	5
1	3	3	2	2	5	5	4	4
2	2	3	4	4	3	4	4	4
2	3	4	4	4	4	3	3	3
3	4	4	3	2	3	2	2	3
2	4	2	3	2	2	3	2	3
2	1	2	2	2	3	4	4	4
1	2	2	2	2	5	4	4	4
3	2	2	4	4	4	4	4	4
3	5	5	4	4	3	4	4	4
3	3	3	3	3	4	3	3	2
4	2	2	2	1	3	4	4	4
2	3	3	2	2	3	3	3	3
4	4	4	3	3	3	3	3	3
3	1	1	2	2	4	3	3	5
2	1	3	2	4	4	4	4	4
3	4	4	4	4	2	3	3	2
2	4	4	4	4	3	4	5	4
4	1	3	1	1	4	4	4	4
3	3	3	3	3	3	3	4	4
2	2	3	4	4	4	4	4	4
1	1	2	2	2	5	5	5	5
2	2	4	4	4	5	5	3	3
4	4	4	5	5	3	3	3	2
3	1	4	2	2	4	4	4	5
2	2	2	4	4	3	3	3	3
3	3	2	4	3	4	4	4	4
3	2	3	3	3	3	4	4	4

3	3	4	3	3	4	4	4	4
2	3	2	2	2	3	4	4	3
1	1	4	3	3	5	3	3	3
4	5	4	3	3	3	2	2	3
5	5	5	5	5	1	1	1	1
4	2	3	3	3	3	5	3	5
1	1	2	3	3	3	4	4	3
2	1	1	1	1	4	4	5	4
2	2	5	5	5	4	3	2	2
1	2	5	4	4	4	4	4	3
1	1	4	2	2	3	4	4	2
3	1	1	2	2	5	5	5	5
2	2	3	3	3	3	4	3	5
4	4	5	4	4	4	3	3	3
2	1	4	1	2	5	5	4	4
1	1	4	5	5	3	3	3	3
4	2	3	2	3	3	2	2	3
4	5	4	2	2	1	3	3	2
3	3	3	3	2	5	5	4	5
4	1	3	5	5	1	3	3	4
2	2	2	3	3	4	4	4	4
1	3	3	3	3	3	3	3	3
3	4	3	4	4	2	3	3	3
1	1	3	5	5	3	5	4	5
2	2	3	3	3	3	3	3	3
3	2	2	3	3	3	4	4	4
2	1	3	3	3	3	5	4	3
2	3	5	2	2	2	3	2	2
1	2	2	2	2	4	4	3	5
3	2	3	4	3	4	3	3	4
3	4	5	4	4	2	2	2	2
2	3	3	1	1	4	5	4	3
5	5	2	4	4	1	2	2	2
1	1	1	1	2	4	5	5	4
3	4	3	3	3	2	3	3	3
2	3	4	2	2	2	4	4	4
3	4	3	4	4	4	4	4	4
3	1	2	4	3	4	4	4	3
2	3	3	4	3	4	4	4	4
3	3	4	3	3	4	4	4	4
3	3	3	3	3	3	3	3	3
2	2	2	3	3	4	3	3	4
2	2	3	2	3	3	3	3	4
4	3	4	2	3	2	3	3	2
3	2	4	4	4	2	2	3	3
4	3	4	5	5	2	3	3	3
5	5	5	3	3	1	1	2	2
5	5	5	4	4	1	1	1	1
3	4	4	5	2	2	2	2	1

F_WC1 (1=Strongly disagree;2= Disagree;3=	F_WC2 (1=Strongly disagree;2= Disagree;3=	F_WC3 (1=Strongly disagree;2= Disagree;3=	F_WC4 (1=Strongly disagree;2= Disagree;3=	W_FE1 (1=Strongly disagree;2= Disagree;3=	W_FE2 (1=Strongly disagree;2= Disagree;3=	W_FE3 (1=Strongly disagree;2= Disagree;3=	F_WE1 (1=Strongly disagree;2= Disagree;3=	F_WE2 (1=Strongly disagree;2= Disagree;3=
5	5	5	1	1	3	1	1	1
3	3	3	3	4	4	3	3	4
3	3	3	3	3	3	3	3	3
2	2	2	2	2	2	2	2	2
1	1	1	1	3	3	2	5	5
2	2	3	2	5	5	5	5	5
1	3	4	1	3	3	2	3	5
3	2	2	2	4	4	4	4	4
2	4	2	2	3	2	2	4	4
1	1	1	1	4	4	4	5	5
3	3	2	2	3	3	3	3	4
3	3	2	2	3	2	3	4	4
1	1	1	1	4	4	2	4	5
3	3	2	2	2	2	2	3	3
3	3	3	3	3	3	3	3	3
3	3	4	4	3	4	4	4	4
3	3	3	3	4	4	4	4	4
2	2	2	2	3	3	3	3	3
1	1	2	1	3	3	4	4	5
2	2	2	2	3	3	4	4	4
1	1	1	1	3	3	3	3	4
5	5	5	5	5	5	5	5	5
1	1	1	1	4	4	2	3	4
1	1	1	1	3	3	4	4	4
3	3	2	2	4	4	3	3	3
1	1	1	1	4	4	3	3	5
1	1	3	2	4	4	4	4	4
1	1	1	1	3	4	3	3	4
3	3	3	3	3	3	3	3	3
1	1	1	1	3	3	3	3	3
3	4	4	3	3	2	2	4	4
3	2	2	1	4	2	2	2	4
2	2	3	2	3	3	2	2	2
3	2	4	3	2	3	1	2	5
2	1	1	1	3	3	3	4	5
1	1	1	1	5	5	5	5	5
1	1	2	1	2	1	1	1	5
1	1	1	1	2	2	2	3	4
3	3	3	2	3	3	3	3	3
3	3	3	3	3	3	3	3	3
2	3	2	1	5	5	5	5	5
2	2	2	1	2	1	1	2	4
3	3	3	3	3	3	3	3	3
1	1	1	1	4	4	3	5	5
2	2	2	2	3	2	3	3	4
3	3	3	2	1	1	1	2	2
2	2	2	2	4	3	5	5	5
3	3	3	3	3	3	4	4	4
2	2	2	2	3	3	3	3	3
2	1	2	1	2	3	2	4	4
2	2	2	2	5	5	5	5	5

4	3	4	4	4	4	3	4	4
4	4	4	4	4	5	4	4	4
3	2	2	1	1	4	3	3	5
3	3	4	3	3	2	1	3	4
2	2	2	2	4	3	2	3	4
3	3	3	3	3	4	3	4	4
3	3	3	3	4	2	3	2	3
1	1	1	1	3	3	3	3	4
2	2	2	2	2	1	2	4	4
5	3	3	3	2	2	1	4	3
3	3	3	3	3	2	1	2	3
3	3	3	3	3	3	3	3	3
4	3	2	2	3	4	4	4	4
2	2	2	2	3	3	4	4	4
3	2	2	2	2	2	2	2	2
2	1	1	1	3	3	3	3	4
2	2	2	2	3	3	3	3	3
2	2	2	2	3	3	3	3	4
2	2	2	2	4	4	3	4	4
3	3	3	3	3	3	3	3	3
4	3	2	2	3	3	3	3	3
3	2	2	1	4	5	4	4	4
3	3	3	3	3	3	3	3	3
3	3	3	2	3	3	2	3	3
1	1	1	1	4	4	4	4	4
5	5	5	1	1	5	1	5	1
3	1	1	1	1	1	1	5	5
2	2	1	1	3	3	3	3	3
5	5	5	5	2	2	2	1	1
3	1	1	1	3	3	2	5	5
2	2	2	2	3	3	3	4	4
2	2	2	2	3	3	3	4	4
3	3	3	3	3	3	3	3	4
2	2	2	2	1	1	1	1	4
3	2	2	2	3	3	2	3	3
1	1	1	1	4	4	5	5	5
3	3	2	2	4	3	3	3	3
3	3	2	2	3	2	2	5	5
2	2	2	2	4	4	4	4	4
3	3	4	4	3	3	3	3	5
3	3	3	3	4	4	3	4	4
5	5	5	5	5	5	3	3	3
2	2	2	2	4	4	4	4	4
2	2	2	2	4	4	3	4	4
2	3	3	2	3	3	3	3	3
3	4	4	3	3	3	3	4	4
2	2	2	2	4	4	3	3	4
1	1	1	1	3	4	4	4	4
2	2	2	2	4	4	3	4	4
4	4	4	4	3	4	3	3	4
3	3	3	2	4	5	4	5	5
4	4	4	4	2	2	2	2	2
2	2	3	3	3	2	3	3	3
2	2	2	2	4	3	3	3	4
3	3	4	3	3	2	2	3	3

2	3	4	2	4	4	4	4	4
3	4	4	4	4	4	5	5	5
1	1	1	1	2	2	2	2	4
3	3	4	2	3	4	2	3	3
2	3	4	2	3	3	3	3	4
1	1	1	1	2	3	2	2	3
3	3	3	2	3	3	3	3	3
3	3	3	2	2	4	2	3	2
2	2	2	2	2	5	2	3	4
3	3	3	3	3	3	3	3	3
3	3	3	3	3	2	2	3	3
3	3	2	2	2	2	2	3	4
2	2	2	2	2	3	3	3	4
4	4	2	2	3	3	3	3	3
3	3	3	2	3	4	3	5	5
4	3	2	2	2	2	2	2	2
3	3	3	2	4	4	4	4	4
3	3	3	3	2	3	3	4	4
4	4	4	4	4	4	4	4	3
3	3	3	2	3	3	2	3	4
3	3	3	3	3	4	3	3	4
2	3	2	3	2	2	5	2	3
1	1	1	1	5	5	5	5	5
2	2	1	1	1	1	1	4	3
4	4	5	5	4	4	4	4	4
2	2	2	2	3	3	2	2	4
2	2	2	2	4	4	4	4	5
2	2	2	2	2	2	2	2	2
2	2	2	2	2	2	2	3	4
1	1	1	1	5	5	5	5	5
3	3	3	3	3	3	3	3	3
4	4	4	3	3	3	2	4	4
5	3	3	2	2	4	3	4	3
4	4	4	4	4	4	4	4	4
2	2	2	2	2	2	2	2	4
5	5	5	5	1	1	1	1	3
5	5	5	4	4	4	3	3	4
3	2	2	2	3	3	2	4	4
1	1	1	1	2	1	1	5	5
3	4	3	2	4	5	2	4	5
2	2	2	2	1	1	1	2	3
2	3	2	1	4	4	3	4	4
2	2	2	2	4	2	2	2	2
2	2	2	1	4	4	4	5	5
2	2	2	2	3	3	3	4	4
5	5	5	2	2	3	1	3	2
2	1	1	1	2	2	3	3	5
3	2	2	2	3	1	3	3	4
2	2	2	2	3	4	4	3	3
1	1	1	1	4	5	5	5	5
2	2	2	2	4	4	4	3	3
1	1	1	1	5	5	5	5	5
4	3	4	3	4	3	2	4	4
3	2	1	1	3	3	3	3	3
3	3	3	3	3	3	3	3	3

3	3	4	4	1	3	1	3	3
2	2	2	2	3	3	3	3	3
3	2	3	3	4	4	3	4	4
3	2	2	2	4	3	4	4	4
3	1	1	1	2	4	3	5	5
3	3	3	3	3	3	3	3	3
4	4	3	3	3	3	3	3	3
2	2	2	2	4	4	3	4	4
5	5	5	3	3	3	1	5	5
2	2	1	1	3	3	3	4	4
2	2	2	1	4	4	4	4	4
2	1	1	1	3	3	3	4	4
2	2	2	2	3	3	3	4	4
5	5	5	5	4	4	4	4	4
1	1	1	1	3	3	4	4	4
3	2	2	2	3	2	1	3	3
3	3	3	3	3	3	3	3	3
4	4	4	4	4	4	4	4	4
1	1	1	1	1	1	1	1	5
2	2	1	1	4	4	3	3	4
3	3	3	3	3	3	3	3	3
2	2	2	2	3	3	3	3	3
3	1	1	1	4	5	5	5	4
3	3	3	3	4	4	4	4	4
2	2	3	2	5	5	3	3	5
1	1	2	1	4	4	4	4	5
1	3	3	3	1	1	1	1	3
2	2	2	2	4	4	4	4	4
1	1	1	1	4	4	4	4	4
2	2	2	2	3	4	4	4	4
4	3	2	2	4	4	3	4	4
2	2	2	2	3	1	2	4	4
4	3	3	3	3	3	3	3	4
3	3	3	2	3	3	3	3	3
1	1	1	1	3	4	3	5	5
1	1	1	1	3	5	5	5	5
2	2	2	2	4	4	2	3	3
1	1	1	1	3	3	1	5	5
2	1	1	1	4	4	3	3	4
3	3	3	3	3	4	3	3	3
1	1	1	1	3	5	5	5	5
2	2	2	2	2	2	3	3	4
2	2	2	2	2	2	2	3	3
4	3	3	3	3	4	3	3	3
2	2	2	2	3	4	3	4	4
3	4	2	3	4	4	4	4	4
2	2	2	2	4	4	3	4	4
3	3	2	2	3	4	3	3	3
2	2	2	2	3	2	2	3	3
1	2	2	2	3	4	3	5	5
2	2	2	2	3	3	3	2	3
3	3	3	3	3	3	3	3	3
3	3	3	3	3	3	3	4	4
3	3	3	3	3	3	1	4	4
3	3	3	2	4	4	3	3	3

2	2	2	2	3	1	2	3	4
2	2	2	2	3	3	3	5	5
5	4	5	4	1	1	1	1	4
2	2	2	2	3	4	4	4	4
3	3	3	3	3	3	3	3	3
1	1	1	1	5	5	5	5	5
1	1	1	1	1	1	1	5	5
1	1	1	1	1	1	1	3	3
2	2	2	2	3	3	3	2	2
2	2	2	2	4	4	4	4	4
3	3	3	3	3	3	3	3	3
2	2	3	1	1	1	1	2	3
2	2	2	2	3	4	3	4	4
3	1	1	1	3	2	1	3	4
2	2	2	2	3	3	3	3	4
4	3	3	2	2	2	2	3	3
2	2	2	2	3	4	4	4	4
5	5	5	5	3	3	3	4	4
3	3	3	3	3	3	2	3	3
3	3	3	3	3	3	3	3	5
2	2	2	1	4	2	2	3	4
2	2	2	1	3	3	3	3	4
2	5	5	2	5	4	4	3	5
3	3	3	3	3	3	3	3	3
3	3	4	3	3	3	2	3	4
5	5	5	5	1	1	1	5	5
3	2	2	2	3	3	3	4	5
3	4	4	3	3	5	3	3	2
2	2	2	2	4	4	3	3	5
3	3	3	3	3	3	3	3	3
1	1	1	1	2	2	2	2	2
3	2	3	3	4	3	3	3	5
1	1	1	1	3	4	4	3	3
2	2	2	2	3	3	3	4	4
3	4	4	3	4	4	4	4	4
2	2	2	2	4	5	5	5	5
3	3	2	2	3	3	3	2	2
3	3	3	3	3	3	3	3	3
1	1	1	1	3	3	3	3	3
4	3	4	4	4	4	4	4	4
4	4	4	4	4	4	4	4	4
3	3	4	2	2	4	4	4	4
2	2	2	2	2	3	3	3	3
2	2	2	1	3	4	4	5	5
1	1	1	1	2	4	3	5	5
1	1	1	1	4	4	3	3	4
4	3	3	3	3	3	3	3	4
4	4	4	3	2	2	2	3	4
2	2	3	2	3	3	2	3	4
3	3	3	2	4	4	3	5	5
2	2	2	2	3	3	3	3	4
1	1	1	2	4	3	3	3	4
2	2	2	2	4	4	4	4	4
3	3	3	3	3	3	3	3	3
2	2	2	2	3	3	3	3	3

2	3	3	2	2	2	2	3	4
4	3	4	4	5	5	5	5	5
2	2	2	2	3	3	3	3	3
1	2	2	2	2	1	1	2	3
4	4	4	3	4	4	4	4	4
2	3	4	4	4	3	3	3	2
1	2	2	2	2	2	3	4	4
2	2	2	2	3	4	3	4	5
2	2	2	1	3	3	4	5	4
3	3	2	3	2	2	3	3	4
3	3	3	4	3	3	3	4	3
2	2	2	1	4	4	4	4	4
3	2	2	1	1	2	1	2	3
3	3	3	3	3	3	3	3	3
5	1	1	1	3	3	4	3	5
3	3	3	3	3	3	3	3	3
3	3	3	3	4	3	3	3	4
3	2	1	1	3	3	3	3	3
3	3	3	3	3	3	3	3	3
2	2	2	2	2	3	3	4	4
2	2	2	2	3	2	2	4	4
1	1	1	1	3	3	3	3	3
1	1	1	1	3	3	3	4	4
1	1	1	1	1	1	1	1	1
2	3	3	2	3	2	3	3	3
2	2	2	2	3	3	3	3	3
4	3	4	2	2	2	2	3	3
2	2	2	2	4	3	3	3	3
2	2	3	2	5	5	5	5	5
2	3	3	2	3	2	2	4	4
1	1	1	1	4	5	4	5	5
2	2	2	2	4	4	4	4	4
2	2	2	2	2	2	2	4	4
2	4	2	1	2	3	2	2	3
2	2	2	2	4	4	4	4	4
3	2	2	2	4	4	4	4	4
2	3	3	2	4	4	4	4	4
2	2	2	2	3	3	3	4	4
2	2	3	2	4	4	4	4	4
2	2	2	2	3	3	3	3	3
2	2	2	2	5	3	5	5	5
2	2	2	2	4	4	4	4	4
3	3	3	3	2	3	3	3	3
2	2	2	2	4	2	4	4	4
3	3	3	3	3	3	3	3	3
3	2	5	5	5	5	5	5	5
5	4	4	4	3	3	2	3	4
1	1	1	1	1	5	1	1	1
1	1	1	1	1	3	3	4	5
2	2	2	2	2	2	2	4	4
3	2	2	1	4	4	3	4	4
1	1	1	1	1	1	1	1	1
2	2	2	2	3	4	2	4	5
1	1	1	1	4	5	5	4	5
3	3	3	1	3	3	3	3	4

3	3	4	3	3	2	3	3	3
2	3	2	2	4	2	4	4	4
1	1	1	1	4	2	4	4	4
2	2	3	2	2	1	2	4	4
1	1	1	1	3	4	4	4	4
2	2	2	2	3	2	3	4	4
2	2	2	2	4	4	4	4	4
3	5	3	2	3	3	2	2	3
2	2	2	2	4	4	3	4	4
3	2	2	2	3	4	4	4	5
2	3	3	2	2	3	3	3	3
2	2	2	1	4	4	4	4	4
2	2	2	2	3	3	3	3	3
2	2	2	2	3	4	3	4	4
2	2	2	2	2	3	3	3	3
5	5	4	4	3	3	3	5	5
1	1	1	1	4	4	4	4	4
4	4	3	4	4	4	4	4	4
3	3	3	2	3	3	3	3	3
2	2	2	2	4	4	3	3	4
1	1	1	1	2	3	3	4	4
2	2	2	2	4	4	4	4	4
1	1	1	1	5	3	3	3	5
2	2	2	2	3	3	3	4	4
1	1	1	1	3	3	2	1	3
2	2	2	2	3	4	4	4	4
1	1	1	1	1	1	1	1	1
2	2	2	2	3	3	3	4	4
3	3	3	3	3	3	3	3	3
1	1	1	1	5	5	5	5	5
1	1	1	1	4	4	4	4	4
1	1	1	1	5	4	5	5	5
2	2	2	2	3	3	3	2	2
2	1	1	1	3	3	3	3	3
1	1	1	1	3	1	2	1	3
2	2	2	2	3	3	3	3	3
1	1	1	1	5	5	5	5	5
1	3	1	1	3	3	3	3	3
1	1	1	1	5	5	3	2	5
2	4	2	1	4	2	3	4	5
2	2	2	2	3	2	2	2	4
2	3	3	2	3	4	3	4	4
2	2	2	2	4	4	4	4	4
2	2	2	2	3	2	2	3	4
2	2	2	2	2	2	2	3	3
2	2	1	1	3	3	2	2	1
3	3	3	3	2	2	2	4	4
1	2	2	2	3	3	2	3	4
3	1	4	1	4	5	3	3	5
2	2	2	2	5	5	5	5	5
4	4	3	3	3	2	2	2	3
2	2	2	2	2	3	3	3	3
1	1	1	1	4	3	3	3	3
3	3	3	3	3	3	3	3	3
1	1	1	1	2	3	2	5	5

2	2	3	2	4	5	4	4	5
2	2	1	1	2	3	3	4	4
3	3	3	3	2	2	2	4	4
2	2	2	2	3	2	2	3	4
3	2	3	2	3	3	3	3	4
4	4	4	4	4	3	3	5	3
2	2	2	1	3	3	2	4	4
3	3	3	3	4	4	4	4	4
2	2	2	1	4	2	2	3	4
3	3	3	3	3	3	3	3	3
3	3	3	3	2	3	3	3	3
5	5	5	5	3	3	2	3	3
3	3	2	1	4	3	1	2	5
2	2	2	2	4	3	3	3	3
1	1	1	1	3	3	3	2	4
3	3	4	2	3	3	3	3	3
1	3	4	1	5	5	3	5	5
1	1	1	1	1	1	1	1	1
2	2	2	2	3	3	3	3	3
3	3	3	3	3	3	3	3	3
1	1	1	1	3	3	3	3	3
2	2	2	1	3	3	3	4	4
2	2	2	2	4	3	2	4	5
1	1	1	1	4	3	3	5	5
2	2	2	2	4	4	3	4	5
1	1	1	1	1	1	1	1	5
3	1	1	1	4	4	3	2	4
4	5	5	5	2	5	5	5	5
2	3	3	3	3	3	3	3	3
1	1	1	1	3	4	2	4	4
3	3	3	3	2	2	2	2	2
3	3	3	3	3	4	2	3	3
3	2	2	2	2	3	3	4	4
1	1	2	1	1	2	1	2	4
2	2	2	2	4	4	4	3	4
2	2	2	2	3	3	3	3	3
2	2	3	2	3	4	3	4	4
2	2	2	2	4	5	5	5	5
2	2	2	2	3	2	2	4	4
2	2	2	2	3	4	4	4	5
3	3	5	4	4	3	2	4	4
3	4	3	3	3	4	3	2	3
3	3	3	3	3	3	3	4	3
1	1	1	1	5	5	5	5	5
4	4	3	3	4	4	4	4	4
3	3	3	3	4	4	4	4	4
4	4	4	4	1	1	1	1	1
1	1	1	1	4	4	3	3	4
2	2	2	2	3	3	3	3	4
3	3	3	2	4	3	3	3	4
3	3	3	3	5	5	4	4	4
1	1	1	1	4	4	4	3	4
2	2	2	2	4	4	4	4	4
3	3	3	3	3	4	4	3	4
4	2	2	2	3	3	3	3	5

1	1	1	1	3	3	4	3	5
1	1	1	1	5	5	5	5	5
2	2	2	2	3	3	2	3	5
3	3	2	2	2	2	2	4	4
2	2	2	2	4	4	4	4	4
2	3	2	4	2	1	1	1	3
2	2	2	2	3	3	3	4	4
1	1	1	1	3	3	3	3	4
3	3	2	2	3	3	2	3	3
4	4	4	4	3	4	3	3	3
2	1	1	1	5	5	5	5	5
3	3	2	2	3	4	4	4	4
2	3	3	1	3	2	3	5	5
1	1	1	1	4	4	4	4	5
2	2	2	1	4	3	3	4	4
1	1	1	1	3	3	3	3	4
1	1	1	1	3	2	2	2	3
2	2	3	2	3	2	3	3	4
1	1	1	1	3	4	3	3	5
3	4	4	3	3	3	3	3	2
2	2	5	2	2	3	2	2	3
2	2	2	1	4	3	2	3	3
2	2	2	2	2	2	2	2	3
3	2	2	2	3	3	3	3	3
3	3	3	2	4	3	4	4	4
2	2	3	2	3	3	4	4	4
2	2	3	2	3	3	2	3	3
5	5	5	5	5	5	5	5	5
4	4	4	3	3	4	3	3	3
2	2	2	2	3	3	3	3	3
3	2	2	1	2	2	2	3	4
5	4	3	4	3	3	3	2	2
1	1	1	1	4	4	3	3	3
4	4	3	3	3	3	3	3	3
3	1	1	1	2	2	3	4	4
2	2	2	2	3	3	2	3	4
3	3	4	3	4	4	3	4	4
3	3	3	3	2	3	2	3	3
3	3	3	3	3	2	2	3	4
1	3	2	2	3	2	2	3	3
3	3	3	3	3	3	3	3	3
3	3	3	3	4	4	4	4	4
1	1	1	1	3	4	3	3	3
2	3	3	2	4	4	3	3	3
3	3	3	3	1	1	1	3	3
2	2	2	2	2	2	2	4	4
1	1	1	1	3	3	3	3	5
3	1	1	1	3	3	3	3	4
2	2	2	2	3	3	3	5	5
2	2	2	2	4	3	3	3	4
3	3	3	3	3	3	3	3	3
5	5	5	5	5	5	5	5	5
2	2	2	2	4	4	4	4	4
2	2	2	1	3	2	2	3	3
1	1	1	1	3	3	3	3	3

3	3	3	3	3	3	3	3	3
2	3	2	2	2	3	3	3	4
3	3	3	3	3	3	3	3	3
1	1	1	1	5	4	4	5	5
3	3	3	2	4	3	3	3	4
4	4	2	2	2	4	4	4	4
3	3	3	3	3	2	2	3	3
2	2	2	2	4	3	3	4	4
1	1	1	1	4	4	4	4	4
1	1	2	1	3	1	2	4	4
1	1	1	1	5	5	5	5	5
2	2	2	2	4	4	2	1	1
3	3	3	3	3	4	4	4	4
3	5	4	2	3	2	3	3	4
2	2	1	2	3	3	2	3	3
3	2	3	2	3	3	3	3	3
2	2	2	2	3	3	3	3	3
2	2	2	2	3	3	4	4	4
2	3	2	2	4	3	3	3	4
2	2	2	2	4	4	4	4	4
3	3	2	1	3	5	4	5	5
2	2	3	2	3	3	3	4	4
3	4	4	3	3	3	2	3	3
2	3	2	2	4	4	4	4	4
3	3	3	2	4	3	3	3	4
2	2	1	2	3	3	2	2	3
2	2	2	2	3	4	3	4	4
2	2	1	1	3	3	3	4	5
3	3	3	3	3	3	3	3	3
3	3	3	3	4	3	3	3	4
1	1	1	1	4	2	5	5	5
2	1	1	2	5	4	5	4	5
1	1	1	1	3	3	3	4	4
3	3	2	2	2	2	2	3	3
3	3	2	2	4	4	4	4	4
1	1	1	1	4	4	4	4	4
3	3	3	3	3	3	3	3	3
3	4	4	4	3	3	2	3	3
1	1	1	1	3	2	2	4	5
2	2	2	2	3	3	2	4	4
4	4	4	4	4	3	3	3	3
2	2	2	2	2	3	2	2	3
2	2	2	2	2	2	2	2	3
4	4	4	4	4	4	4	4	4
3	3	3	3	3	3	3	4	5
1	1	1	1	1	5	5	5	5
1	1	1	4	2	3	1	3	5
2	2	1	1	5	3	2	4	4
3	3	1	3	1	2	1	3	3
3	2	2	2	3	3	4	4	4
2	3	2	1	4	1	1	2	4
1	1	1	1	4	3	3	3	4
3	2	2	2	3	3	3	4	4
4	3	4	3	4	3	3	3	4
2	1	2	2	4	5	2	5	5

1	1	1	1	4	4	3	3	4
1	1	1	1	4	4	3	3	4
2	2	2	2	4	4	3	4	4
3	2	2	2	4	4	4	5	5
2	3	4	2	3	2	4	4	4
1	1	1	1	5	3	3	3	3
2	2	2	2	2	3	2	3	4
2	2	2	1	4	1	2	4	4
3	3	3	3	4	3	2	2	3
1	1	1	1	4	4	4	4	4
1	3	4	1	4	4	4	4	5
2	2	2	2	4	4	4	4	2
2	1	1	1	5	5	5	5	5
3	3	3	3	3	4	3	3	4
3	2	2	1	4	4	3	3	4
3	3	3	3	3	3	3	3	3
2	2	1	2	4	3	3	3	3
1	1	1	1	4	3	3	4	4
2	3	2	2	3	3	2	3	4
1	1	1	1	4	4	3	3	3
3	3	3	3	3	3	3	3	3
3	3	3	3	3	3	3	3	3
5	5	5	1	5	5	5	5	5
1	1	1	1	2	3	3	4	3
2	2	2	2	3	2	2	3	4
4	4	4	4	4	4	4	4	4
1	1	1	1	5	5	5	5	5
2	3	3	2	1	1	1	1	3
5	5	3	5	3	3	2	2	5
1	1	3	1	3	3	3	3	5
3	3	3	2	3	3	3	3	4
2	2	1	2	3	3	3	3	3
4	4	4	3	4	4	3	4	5
2	2	2	2	3	3	2	4	4
1	1	1	1	2	3	3	3	4
3	3	2	2	4	3	2	2	5
2	1	2	2	4	2	2	4	4
5	5	5	5	2	2	1	1	2
3	2	3	2	4	4	2	2	4
3	3	3	2	2	2	2	2	3
3	3	4	3	3	3	2	2	4
3	2	1	1	4	3	4	4	4
3	2	3	2	4	4	4	4	4
1	1	1	1	5	5	5	5	5
2	2	2	2	3	3	3	3	4
4	3	3	3	3	3	3	3	3
5	5	3	3	3	1	1	3	3
3	3	3	3	3	3	3	3	3
1	1	1	1	3	3	3	3	4
2	2	2	1	3	2	2	3	3
3	4	4	4	3	4	3	4	4
1	1	1	1	4	2	2	3	3
2	2	1	1	5	1	1	3	4
4	4	3	3	3	4	4	3	3
2	2	3	3	3	3	3	4	4

2	2	2	2	3	3	3	3	3
1	1	1	1	3	3	3	3	4
5	5	1	1	1	1	1	3	4
2	2	2	2	4	4	3	4	4
2	2	2	2	3	3	3	3	4
4	2	2	2	1	2	1	2	3
3	3	3	3	4	4	4	4	4
3	3	3	3	3	3	3	3	3
1	1	1	1	3	1	3	4	5
1	1	1	1	2	1	2	2	4
2	2	2	2	4	4	3	3	3
3	3	3	4	3	3	3	3	4
2	2	3	1	3	3	2	3	3
2	1	1	1	3	3	3	3	3
2	2	3	2	4	4	3	3	3
1	1	1	1	5	5	3	5	5
3	3	3	3	3	3	4	4	4
3	3	3	3	3	3	3	3	3
5	5	5	5	3	3	1	3	3
1	1	1	1	5	5	2	2	5
2	3	3	2	3	4	3	3	3
2	2	2	2	4	4	3	3	4
2	2	2	2	3	3	3	3	4
1	1	1	1	5	5	5	5	5
2	2	2	2	3	3	3	4	4
2	2	2	1	3	4	3	3	3
3	3	3	3	3	3	3	3	3
3	3	3	3	3	3	3	3	3
4	5	4	2	3	3	2	2	3
2	2	2	2	2	3	3	4	4
2	2	2	2	4	3	3	3	3
4	4	4	4	3	3	3	3	3
3	3	3	3	3	3	3	3	3
2	2	2	2	3	4	3	3	4
2	1	1	1	4	4	3	5	5
4	3	3	3	3	2	2	2	3
2	2	2	2	2	3	3	3	4
2	2	2	2	4	4	4	4	4
2	1	4	1	4	4	3	5	5
2	2	2	1	4	2	3	2	5
3	3	3	3	3	3	2	4	4
1	1	1	1	3	2	4	4	5
2	2	2	2	3	4	3	3	3
2	2	2	2	3	2	2	3	4
1	1	2	1	4	4	4	5	5
2	2	2	1	3	3	3	3	3
2	3	3	3	4	3	3	3	4
2	2	2	2	2	3	3	3	4
5	5	5	5	4	4	2	3	4
2	2	2	2	3	3	3	3	3
1	1	1	1	2	2	2	1	1
2	2	1	1	4	5	4	3	5
1	1	1	1	5	5	5	5	5
2	2	3	1	3	3	2	3	5
2	2	2	2	3	3	4	4	4

2	2	2	2	3	3	3	3	4
1	1	1	1	5	5	5	5	5
4	4	3	3	3	3	3	3	3
3	2	3	2	1	2	2	4	4
4	4	3	2	4	4	4	4	4
1	1	1	1	3	3	4	4	4
2	2	3	2	4	3	3	3	4
4	2	2	2	2	3	2	2	3
1	1	1	1	2	3	3	3	4
1	1	1	1	1	1	1	4	4
3	3	3	3	3	3	3	3	3
4	4	3	4	2	3	2	4	4
4	5	5	3	2	4	5	5	5
1	1	1	1	5	3	1	3	2
2	2	2	2	3	3	3	4	4
2	2	2	2	3	3	3	4	4
2	2	2	2	2	2	2	3	4
1	1	1	1	5	5	5	5	5
3	3	3	3	3	3	3	3	3
4	3	5	3	4	3	3	3	4
3	3	2	2	2	2	3	3	4
2	2	2	3	3	3	3	3	3
3	3	3	2	3	3	3	3	3
1	1	1	1	4	4	4	4	4
3	2	2	1	2	2	2	2	3
2	2	3	2	3	3	3	4	3
1	1	1	1	3	3	3	3	3
4	3	3	3	3	3	3	3	3
1	1	1	1	3	3	3	3	5
3	3	3	3	3	3	3	3	3
2	2	2	2	3	3	4	4	4
4	3	4	2	3	3	3	3	3
3	3	3	3	3	3	3	3	3
2	2	3	2	3	3	3	3	3
5	5	5	5	1	3	1	5	5
2	2	3	3	3	2	2	2	3
4	4	4	4	4	4	4	4	4
3	3	2	2	4	4	4	4	4
3	2	2	2	3	3	2	3	3
1	1	1	1	4	4	4	4	5
1	2	2	2	4	4	3	3	5
1	1	1	1	5	5	5	5	5
1	1	1	1	3	3	3	4	4
5	3	2	2	3	3	3	3	4
4	4	5	3	3	3	3	3	3
1	1	1	1	3	3	3	4	5
2	2	2	2	3	2	2	3	3
4	4	4	2	3	3	2	4	4
1	1	1	1	5	5	5	5	5
3	3	3	3	3	3	3	3	3
2	2	2	1	3	3	3	3	4
3	3	3	3	2	2	2	3	3
4	5	5	5	5	4	2	4	4
3	3	2	2	4	3	2	3	3
2	2	2	2	3	4	4	4	4

1	1	1	1	5	5	4	4	4
2	2	2	2	4	4	4	4	4
3	2	3	2	4	4	3	2	4
4	4	4	4	4	4	4	4	4
2	2	4	2	2	4	4	4	4
1	1	1	1	4	4	4	4	4
3	2	2	2	4	3	3	3	3
1	1	1	1	1	5	5	5	5
2	2	2	2	3	1	2	4	5
2	2	2	2	3	3	3	3	3
3	3	3	3	2	1	1	3	2
3	3	3	3	3	3	3	3	3
2	2	2	2	3	3	3	4	4
4	4	5	4	3	3	3	3	4
2	2	2	2	3	3	3	3	4
1	1	1	1	1	1	1	1	1
2	3	3	3	3	3	3	3	3
1	1	1	1	3	3	3	4	4
4	3	3	3	4	4	3	4	5
2	2	2	1	1	3	2	2	2
2	1	1	1	3	4	4	4	4
2	2	3	2	2	4	4	4	4
3	2	2	2	4	4	4	4	4
1	1	1	1	5	5	5	5	5
5	5	5	4	1	1	1	3	2
2	2	2	2	5	5	3	3	4
1	1	1	2	3	3	3	4	4
3	3	3	3	3	3	3	3	3
2	2	2	2	3	2	2	3	3
2	2	2	2	3	3	3	3	4
5	5	5	5	3	3	3	1	3
1	1	1	1	3	1	3	5	5
4	4	4	4	4	4	4	4	4
4	4	4	4	3	3	3	4	4
3	3	3	3	3	3	3	4	4
3	2	4	2	4	4	2	3	4
3	2	2	2	3	4	4	4	5
3	3	1	1	3	1	3	3	5
2	2	3	2	3	3	3	3	4
4	3	4	3	4	3	1	1	3
2	1	1	1	4	5	5	5	5
2	2	2	2	4	4	3	4	4
1	1	1	1	3	4	4	4	4
2	2	2	2	4	4	3	3	3
1	1	1	1	2	2	2	4	4
3	3	3	3	3	3	2	3	4
1	1	1	1	3	3	4	4	4
2	2	2	2	3	3	3	4	4
3	4	3	2	4	3	2	3	3
3	3	4	3	1	1	1	4	5
2	1	5	2	3	4	4	2	4
2	1	1	1	4	3	3	3	4
2	2	2	1	3	4	3	4	5
3	3	3	3	3	3	3	3	3
2	2	2	2	3	3	3	3	3

1	1	1	1	5	5	5	5	5
3	3	4	3	2	3	2	3	2
2	3	2	2	3	2	3	3	2
5	4	4	3	2	2	1	3	3
3	3	3	3	3	3	3	3	4
2	1	2	1	3	3	1	4	4
5	5	5	4	2	2	2	3	3
1	1	1	1	1	1	1	1	4
3	3	3	3	3	2	2	3	4
4	4	5	4	3	4	3	3	4
2	2	2	2	3	3	2	2	2
2	2	2	1	2	1	1	3	3
2	2	3	2	4	2	2	3	3
3	3	3	3	3	3	3	3	3
3	2	2	2	5	5	5	5	5
1	1	1	1	3	2	2	5	5
2	1	1	1	2	3	3	3	5
3	3	3	2	3	3	3	3	3
1	2	2	2	2	3	3	4	4
2	2	2	2	2	3	2	2	3
2	2	1	1	4	4	4	4	5
2	2	3	2	3	3	3	3	3
2	2	2	2	2	2	2	4	4
3	3	3	3	3	3	3	3	3
2	1	2	1	4	4	4	5	5
1	1	1	1	1	1	1	1	1
2	2	2	2	2	3	2	4	4
3	3	3	3	3	3	3	3	3
1	1	1	1	4	4	3	4	5
3	3	3	3	3	4	3	4	4
1	1	1	1	5	5	5	5	5
3	3	3	4	4	4	3	4	5
1	1	2	1	4	3	3	3	4
2	2	2	2	4	4	4	4	4
3	3	3	3	4	4	3	2	3
2	2	2	2	2	2	2	4	4
1	1	1	1	3	2	3	5	5
2	2	2	2	3	3	3	3	4
4	3	2	3	2	3	3	4	3
4	1	1	1	3	1	4	4	4
1	1	1	1	3	3	3	3	3
2	2	2	2	3	3	3	4	4
3	3	3	3	3	3	3	3	3
1	1	1	1	2	2	2	2	3
1	1	1	1	3	4	1	4	5
2	2	2	2	3	3	3	3	3
2	3	2	2	4	4	4	4	4
2	2	2	2	2	2	2	4	4
1	1	5	1	3	3	3	4	4
2	2	1	1	3	2	2	3	4
5	5	5	5	2	1	1	1	2
1	1	1	1	3	1	1	1	3
1	1	1	1	4	4	4	4	4
3	3	3	3	3	3	3	3	3
1	1	1	2	4	4	4	4	4

2	2	2	2	3	3	2	3	4
2	2	3	1	4	3	3	3	5
2	1	2	2	3	3	2	4	4
2	2	2	2	3	2	2	4	4
1	1	1	1	1	1	1	5	5
5	5	5	5	2	4	2	4	4
3	3	2	3	3	3	3	3	3
3	3	3	2	3	4	3	4	4
5	5	3	2	4	4	2	4	3
1	1	1	1	4	4	3	3	4
3	3	3	3	3	3	3	3	3
3	3	3	3	2	3	3	3	3
2	2	2	1	4	1	1	3	2
1	1	1	1	1	1	1	5	5
4	3	3	3	4	3	3	3	5
3	3	3	3	3	3	3	3	3
4	3	3	3	2	2	2	4	4
1	1	1	1	5	5	5	5	5
2	2	2	1	4	4	3	4	4
3	3	3	3	3	3	3	3	3
3	3	3	2	3	3	3	3	3
2	2	2	2	3	3	3	4	4
4	4	4	4	4	3	3	4	4
3	3	3	3	3	3	2	4	4
4	4	4	4	3	3	4	4	4
1	1	1	1	3	4	3	4	4
2	2	2	2	4	4	4	4	4
1	1	1	1	1	1	1	4	4
2	2	2	2	4	3	3	4	4
2	2	2	2	4	4	4	4	4
1	1	1	1	1	4	4	4	4
3	3	3	3	3	3	3	3	3
4	4	4	4	3	3	3	3	3
2	2	2	2	3	2	2	2	4
2	2	2	2	2	2	2	4	4
4	4	4	4	3	3	3	3	3
3	3	2	3	4	5	4	3	3
1	1	1	3	2	1	1	4	5
3	3	3	2	3	4	3	3	4
3	2	1	1	4	3	4	3	3
2	2	2	2	2	2	2	2	3
3	2	2	2	4	4	3	3	5
2	2	2	2	1	3	2	4	4
2	2	2	1	4	3	2	2	4
3	3	3	3	4	4	3	4	4
3	3	3	3	3	4	3	4	4
1	1	2	3	3	3	3	4	4
2	2	2	2	5	3	4	4	5
2	2	2	2	3	3	2	4	4
5	5	5	5	4	3	2	4	4
3	3	2	2	4	4	4	4	4
1	2	2	1	3	3	3	4	4
2	2	2	2	4	4	4	4	4
3	2	2	2	4	3	3	3	4
1	1	1	1	3	3	3	3	3

1	1	1	1	4	4	4	4	4
2	4	2	2	3	3	3	3	3
3	3	2	2	3	2	3	4	4
2	2	2	2	2	2	3	3	3
3	3	3	3	2	2	3	2	3
2	2	3	2	3	2	3	2	3
1	1	1	1	4	3	3	5	5
3	2	2	2	3	3	3	3	4
2	2	2	2	2	2	2	3	4
3	3	3	3	3	3	3	3	3
1	1	1	1	4	3	3	4	4
3	3	3	2	2	2	2	2	3
5	5	5	5	1	1	1	2	2
3	3	3	3	2	3	2	2	3
1	1	1	1	1	3	1	3	4
1	1	1	1	4	4	5	5	5
4	4	4	4	3	3	3	3	3
3	3	2	2	3	3	3	3	3
1	1	1	1	5	5	5	5	5
1	1	1	2	4	3	3	5	5
2	2	2	2	3	3	3	3	3
1	1	2	1	2	1	1	2	3
5	5	3	4	4	3	4	4	3
2	3	3	3	4	2	3	3	4
1	2	3	1	4	4	3	5	5
2	2	2	2	2	1	1	3	4
2	2	2	2	3	3	3	3	3
2	2	2	2	3	3	3	4	4
2	3	3	2	4	4	4	4	4
3	2	2	2	2	2	3	4	5
3	3	3	3	3	3	3	3	3
4	4	3	3	3	3	2	2	2
4	2	3	1	2	3	1	3	3
4	3	5	2	4	2	2	4	5
4	4	4	4	4	4	4	4	4
2	1	1	2	2	3	3	2	4
1	1	1	1	5	5	5	5	5
3	3	2	1	5	3	3	5	5
1	1	1	1	5	5	5	5	5
2	2	3	2	2	3	3	3	3
4	3	3	3	3	3	3	3	3
3	2	2	2	1	3	2	4	3
2	2	2	2	3	4	4	4	4
3	3	3	3	3	3	3	3	3
2	1	1	1	3	1	2	3	3
2	3	4	1	4	4	2	2	4
4	4	4	4	4	4	4	4	4
4	4	4	4	4	4	4	4	4
3	3	2	2	2	2	2	4	4
3	2	2	2	2	2	2	3	3
3	3	3	3	2	3	2	3	3
2	2	2	2	4	3	3	4	4
3	3	3	3	3	3	3	5	5
1	2	1	1	5	5	5	5	5
1	1	1	1	3	2	2	3	5

1	1	1	1	1	1	1	4	3
2	2	2	2	3	3	4	4	5
3	4	3	3	3	3	3	3	3
4	3	3	3	3	3	3	4	4
2	2	2	2	3	3	4	4	4
1	1	1	1	2	1	1	5	5
3	3	3	3	3	3	3	3	3
4	4	3	3	3	3	2	3	4
4	4	4	4	4	4	4	4	4
1	1	1	1	5	5	5	5	5
3	3	3	3	3	3	3	3	3
2	2	2	2	4	4	4	4	4
3	3	3	3	3	3	3	3	3
2	2	2	2	5	5	5	5	5
4	4	4	4	4	4	4	4	4
4	4	4	4	4	4	2	2	3
1	1	1	1	1	4	3	4	5
2	2	2	2	2	5	5	5	5
1	1	1	1	4	3	3	4	5
3	2	2	2	4	4	4	4	5
2	2	2	2	3	3	3	4	4
1	1	1	1	2	2	1	3	3
2	2	2	1	2	2	1	2	4
1	1	1	1	3	3	3	4	4
2	2	3	2	3	3	3	4	5
2	2	3	2	2	3	2	2	3
3	3	2	2	3	3	3	3	4
2	2	2	2	4	4	4	4	5
1	1	1	1	3	2	1	4	5
2	2	2	2	4	4	4	4	4
3	4	3	3	3	3	3	4	3
3	3	3	3	3	3	3	3	3
2	2	2	2	3	3	2	4	4
2	2	2	2	2	2	2	2	4
1	1	1	1	4	1	1	1	3
2	1	2	1	2	3	2	2	3
4	4	4	4	3	3	3	3	3
5	3	4	4	2	2	3	4	4
3	3	2	2	3	3	2	3	3
3	2	2	3	4	4	3	3	3
3	2	2	2	3	3	3	3	3
2	2	1	1	4	4	3	4	4
2	3	3	2	3	4	3	3	4
1	1	1	1	5	5	5	5	5
2	2	2	2	3	2	2	4	4
1	2	1	2	2	4	3	4	4
3	3	3	3	2	3	2	3	3
2	2	2	2	4	3	3	5	5
2	2	2	3	3	3	3	4	4
2	2	2	2	4	4	4	4	4
2	2	2	2	3	2	2	3	4
5	5	5	2	2	2	2	4	3
2	3	2	2	4	3	3	2	5
3	2	2	2	4	4	4	4	4
5	3	5	3	1	1	1	3	4

2	2	2	1	1	1	1	1	3
3	2	3	2	2	2	2	3	4
2	2	2	3	2	2	2	3	3
3	2	2	2	4	3	3	3	4
2	1	1	1	3	3	2	4	5
3	3	3	2	2	2	2	1	1
5	5	5	5	3	3	3	3	3
3	2	2	2	3	3	3	3	3
2	2	2	2	2	2	2	3	3
4	4	4	4	4	4	3	4	4
3	3	3	3	3	3	3	3	3
2	1	2	1	3	2	2	4	4
2	2	2	2	5	5	5	5	5
3	3	2	3	3	3	3	3	3
1	1	1	1	4	5	4	4	5
2	2	2	2	3	4	4	4	4
4	4	4	4	4	4	3	3	3
2	2	3	2	3	2	2	4	4
3	3	3	3	3	3	2	2	3
2	2	2	2	3	3	3	4	4
1	1	1	1	4	4	4	4	4
2	2	2	2	4	3	4	4	5
2	1	1	2	4	4	4	4	4
3	3	3	3	2	2	2	4	4
1	1	1	1	3	4	5	5	5
1	1	1	1	1	5	5	5	5
2	2	2	2	3	3	3	3	4
3	2	3	2	2	2	2	4	4
1	1	1	1	3	3	3	5	5
2	1	1	1	2	4	3	3	4
3	4	4	4	4	4	4	4	4
2	2	2	2	4	4	2	2	3
2	3	3	1	3	3	2	4	5
2	2	1	1	4	4	4	5	4
3	3	3	2	2	2	3	4	4
4	4	4	4	4	4	3	3	3
3	4	4	4	4	4	4	4	4
3	3	3	3	3	3	3	3	3
3	3	4	3	4	4	4	4	4
1	1	1	1	4	4	4	4	5
3	3	2	2	3	4	3	4	4
3	2	1	2	4	4	3	3	5
2	2	2	2	2	2	4	4	4
2	2	2	2	3	2	2	2	4
1	2	2	2	4	4	5	4	4
1	1	2	1	2	1	1	5	5
2	2	2	2	4	4	4	4	4
4	3	2	2	3	3	3	4	4
2	1	1	1	3	4	4	4	4
4	3	5	3	2	2	2	3	3
3	2	3	1	2	2	2	4	4
3	4	3	2	1	1	1	4	4
3	3	3	3	2	2	2	2	2
1	1	1	1	5	5	5	5	5
3	3	3	2	4	3	3	4	4

4	4	3	3	3	3	3	3	3
1	1	1	1	4	4	3	2	5
3	3	3	3	3	3	3	3	3
3	2	3	2	4	4	4	5	5
2	3	3	2	2	3	3	3	4
4	4	3	2	4	4	3	4	3
3	3	5	1	4	5	4	5	5
3	2	2	2	4	4	4	4	4
2	2	2	2	3	3	3	3	4
2	2	2	3	2	2	2	4	5
1	1	1	1	3	2	1	3	1
1	1	1	1	5	5	5	5	5
2	2	1	1	2	1	3	3	5
2	2	2	2	1	1	1	1	2
2	2	2	2	4	4	3	4	4
4	3	2	1	3	3	3	4	4
3	3	3	2	2	4	3	4	5
1	1	1	1	4	4	4	4	4
4	4	5	5	3	2	2	3	2
2	2	2	2	3	2	2	2	3
2	2	2	1	4	4	3	5	5
3	5	4	2	5	1	1	3	5
4	3	2	2	3	3	3	3	3
2	2	2	2	3	4	4	4	4
4	3	5	3	3	3	3	3	3
2	2	2	2	5	4	5	5	5
2	2	3	1	3	3	3	3	5
2	2	2	2	3	3	3	3	3
2	2	2	2	4	4	4	4	4
4	2	3	2	4	4	4	4	4
4	4	4	3	1	2	2	2	5
2	2	2	2	2	2	2	2	2
4	3	5	3	1	4	3	3	5
2	2	2	2	5	5	5	5	5
1	1	1	1	4	5	5	5	5
1	1	1	1	4	4	4	4	5
2	2	1	1	4	4	4	4	4
2	1	1	1	4	3	3	4	4
4	4	4	4	1	3	3	4	4
2	3	1	1	3	3	3	5	5
3	3	3	3	3	2	2	2	3
2	2	2	2	3	2	2	3	4
3	3	3	2	2	2	2	4	4
2	2	2	2	4	4	4	4	4
2	3	3	2	3	3	3	5	5
3	3	3	2	3	3	3	3	3
2	2	1	2	2	4	4	3	5
5	4	5	3	3	3	1	3	3
2	2	2	2	4	4	4	4	4
4	4	4	4	4	4	4	4	4
4	3	4	2	4	4	4	4	4
4	3	3	3	3	3	3	3	3
5	5	5	5	5	1	1	3	3
1	3	4	2	5	5	4	4	1
1	1	1	1	4	4	4	4	4

1	3	4	3	4	4	2	4	4
1	1	1	1	5	5	5	4	5
4	4	4	3	3	3	2	3	4
3	2	3	3	4	2	1	1	3
2	2	2	3	5	5	5	5	5
5	5	5	4	4	3	3	4	4
5	5	5	5	1	1	1	5	5
2	2	3	1	4	4	4	4	4
2	2	2	2	4	4	4	4	4
2	2	3	2	1	1	3	3	5
2	2	2	2	2	2	2	4	4
2	2	2	2	3	3	3	3	3
2	3	5	2	2	3	3	4	4
1	1	1	1	2	1	1	1	4
3	3	3	2	3	4	2	3	3
5	5	5	5	2	3	2	1	3
3	3	3	3	3	3	5	5	5
1	1	1	1	5	5	5	5	5
2	2	2	2	2	3	3	2	3
4	3	2	4	2	2	2	4	4
2	2	2	2	3	2	3	3	3
3	3	2	2	3	3	3	3	4
4	4	5	2	4	3	2	5	3
2	2	1	1	3	3	3	3	3
2	2	1	1	3	3	3	3	4
3	4	5	3	4	4	2	4	4
3	2	5	1	4	3	3	4	4
3	3	3	3	3	3	3	3	3
3	4	4	3	3	5	3	2	2
2	2	2	2	3	3	3	4	4
1	2	3	2	4	4	4	4	4
2	2	2	2	4	3	3	4	4
3	3	3	3	3	3	3	3	3
3	3	3	3	3	3	3	3	3
1	1	1	1	4	4	4	4	4
3	3	3	3	3	3	3	3	3
1	1	1	1	5	5	5	5	5
1	1	1	1	3	4	4	4	5
2	2	2	2	5	5	5	5	5
2	2	3	2	3	3	3	2	4
2	3	2	1	4	4	3	4	5
3	3	3	3	3	3	3	3	3
3	3	3	3	3	3	3	3	3
2	2	2	2	3	4	3	4	4
4	4	4	4	2	2	2	2	2
2	2	2	2	4	4	4	3	4
3	3	3	3	2	2	2	2	2
5	5	2	2	3	2	2	3	3
3	3	3	3	3	3	3	3	3
3	3	3	3	3	3	3	3	3
3	2	2	1	2	3	3	3	3
5	5	5	5	1	1	1	1	1
3	3	3	2	3	3	3	4	4
1	1	2	1	3	3	3	5	5
3	3	3	3	4	3	3	3	3

1	1	1	1	1	4	3	3	4
1	5	5	5	1	1	1	1	4
1	1	1	1	5	5	5	5	5
2	2	2	2	4	4	4	4	4
2	2	2	2	2	3	2	2	2
2	2	2	2	2	2	2	2	2
3	2	2	2	4	4	4	4	4
1	1	1	1	3	1	1	1	1
4	4	4	3	2	4	3	3	3
1	1	1	1	5	5	5	5	5
1	1	1	1	3	2	3	2	5
1	1	1	1	5	3	3	5	5
2	2	1	2	3	3	3	2	2
2	2	2	2	2	2	2	2	3
1	1	1	1	5	5	5	5	5
3	3	3	2	3	3	3	3	3
1	1	1	1	4	4	4	4	4
2	2	2	2	3	3	3	2	3
1	1	1	1	1	3	3	4	4
2	1	2	1	4	3	3	4	5
1	1	1	1	5	5	5	5	5
2	2	4	1	3	4	3	5	4
3	3	3	3	3	3	3	3	3
5	5	1	1	4	4	4	5	5
2	3	2	2	4	3	3	3	4
1	5	1	1	4	5	5	5	4
4	4	4	2	5	5	5	5	5
3	2	3	3	3	3	3	3	3
1	1	1	1	4	3	4	4	5
4	2	2	2	3	4	3	4	3
2	2	2	2	2	2	2	4	4
2	1	1	1	3	3	3	5	5
4	4	4	4	1	3	3	3	3
2	2	2	2	3	3	3	4	4
2	3	2	1	4	4	4	4	5
2	2	2	2	3	3	2	3	3
4	4	4	3	2	2	2	3	4
2	2	2	1	2	4	4	5	5
2	2	4	3	2	2	2	2	3
1	3	1	1	4	3	3	3	4
2	2	2	2	3	4	3	4	4
3	2	2	2	2	3	2	3	4
2	2	2	2	4	4	5	5	5
2	2	2	3	3	3	3	4	5
3	3	5	3	3	3	3	3	3
3	3	3	3	3	3	3	3	3
2	2	3	2	3	4	4	3	4
5	5	5	5	4	4	4	4	3
2	2	2	2	4	4	4	4	4
3	3	3	2	3	2	3	3	4
2	1	1	1	3	2	2	2	3
2	2	1	1	2	2	3	5	5
5	5	5	5	5	5	5	5	5
1	1	1	1	5	5	5	5	5
1	1	1	1	3	3	3	3	3

1	1	1	1	4	4	4	4	4
3	4	4	4	2	3	3	3	3
2	2	2	2	1	3	3	2	3
1	1	1	1	5	5	5	5	5
3	3	3	2	3	3	3	5	5
2	2	2	2	3	3	1	4	4
4	4	4	3	3	3	3	3	3
1	1	1	1	4	2	2	4	5
2	2	2	2	2	4	4	4	5
1	1	1	1	3	5	5	5	5
2	1	1	1	4	4	4	4	5
3	2	3	1	4	4	3	4	5
2	2	3	2	4	4	3	1	5
3	3	3	3	3	3	4	4	4
2	2	2	2	3	4	4	4	5
5	1	1	1	2	3	2	4	3
2	2	3	2	4	4	3	4	5
2	2	2	2	2	2	2	3	3
4	4	4	4	3	3	3	3	4
1	3	2	2	5	5	5	5	5
4	4	4	4	4	4	4	4	4
3	3	3	3	3	2	1	2	4
2	5	4	2	5	5	5	5	5
3	2	2	2	2	2	2	3	3
2	2	2	1	4	3	3	5	5
2	1	2	1	4	4	4	4	4
5	5	5	5	5	5	5	5	5
2	2	2	2	4	4	4	4	4
3	2	2	1	3	4	1	3	4
2	1	1	1	4	1	3	4	5
1	1	1	1	5	5	5	5	5
1	1	1	1	3	3	3	3	3
3	2	1	1	1	3	2	3	3
3	3	3	2	3	3	3	3	3
2	2	4	3	3	3	3	3	3
2	2	3	2	3	3	3	3	3
2	2	2	2	3	3	3	3	4
3	3	3	3	3	3	3	3	3
3	3	3	3	4	3	3	3	3
3	2	2	2	3	3	3	4	4
1	1	1	1	1	1	1	1	1
2	2	2	2	3	3	3	3	3
5	5	3	3	3	1	1	3	5
1	1	1	1	1	3	3	3	3
3	3	3	3	3	3	3	3	3
3	3	3	2	3	3	2	3	4
2	2	2	2	2	2	2	4	4
3	2	2	2	4	4	4	4	4
2	2	4	2	4	4	2	3	4
1	1	1	1	4	3	3	4	5
2	2	1	1	2	2	2	2	2
2	2	2	2	4	4	3	4	4
3	3	3	3	3	5	5	5	5
3	2	1	1	3	2	4	5	5
1	1	1	1	5	5	5	5	5

1	1	1	1	4	1	1	4	4
3	2	2	2	3	3	3	3	3
3	3	3	3	3	4	3	3	3
1	1	1	1	1	3	3	3	3
3	2	2	2	4	3	3	3	4
3	5	3	3	4	4	4	3	4
3	2	2	2	3	3	2	3	3
2	3	3	3	2	3	3	3	5
3	3	3	3	3	5	4	5	5
4	4	3	2	2	4	3	3	3
2	2	2	1	3	2	3	4	3
2	2	3	2	2	2	2	3	3
2	2	2	2	3	2	3	3	4
5	2	4	1	5	3	1	4	5
2	2	2	2	3	2	2	3	3
2	3	2	1	2	1	2	2	3
1	1	1	1	3	3	3	3	3
2	2	2	2	3	2	3	3	4
1	1	1	2	4	4	3	4	4
4	4	3	2	4	4	3	3	3
2	4	3	2	2	1	2	2	4
1	1	1	1	5	5	5	5	5
2	2	2	2	2	2	2	2	4
1	1	1	1	5	2	4	5	4
4	3	3	3	3	3	3	3	3
2	2	2	2	2	2	2	3	3
2	2	2	2	4	4	3	4	4
4	3	3	3	3	3	2	2	2
2	2	2	2	3	4	3	4	3
2	2	3	2	2	3	2	3	4
2	2	2	2	4	3	3	4	4
3	2	4	2	4	3	3	4	4
1	1	1	1	3	3	3	4	4
1	1	1	1	4	3	4	4	5
3	2	2	2	3	2	2	2	2
3	3	3	3	4	4	3	4	4
1	1	1	1	4	4	4	4	4
4	4	4	3	3	3	3	3	3
3	3	3	3	2	2	3	4	4
4	4	2	1	2	1	1	1	3
5	5	5	5	4	3	3	3	3
2	2	2	2	4	4	3	4	4
2	3	4	2	3	3	3	3	3
4	3	3	2	4	1	2	3	4
2	2	2	2	3	4	2	4	4
2	3	2	2	4	4	4	4	4
2	2	2	1	3	3	4	3	3
1	1	1	1	3	4	3	3	5
1	1	1	1	4	4	4	4	4
2	3	2	1	3	2	3	3	3
3	3	3	3	3	3	3	3	3
2	2	2	2	3	2	2	3	3
2	1	1	1	3	2	2	5	5
2	2	2	2	2	2	2	2	2
2	2	2	2	4	4	3	4	4

2	2	2	2	3	3	4	4	4
1	2	3	2	4	3	3	4	4
5	2	4	2	2	5	4	4	4
1	1	1	1	5	3	1	1	5
4	4	4	2	3	3	3	3	4
2	2	1	1	3	2	1	4	3
2	3	4	2	4	3	2	2	2
4	4	4	3	2	2	2	2	4
1	1	2	2	4	4	4	4	4
2	2	2	2	3	2	2	3	3
3	2	2	2	3	2	2	3	3
2	2	2	2	3	2	2	2	3
1	1	1	1	3	3	2	3	3
1	2	3	2	3	4	3	5	5
1	1	1	1	1	2	4	2	4
1	1	1	1	4	4	4	5	5
3	3	3	2	3	5	3	5	5
2	2	3	2	3	3	2	4	4
2	2	2	2	3	3	3	4	4
2	4	3	3	4	3	3	3	3
3	3	3	3	3	3	3	3	4
2	2	2	2	3	3	3	4	4
2	2	2	2	3	3	3	3	3
2	2	2	4	3	2	2	2	3
3	3	3	2	4	4	4	4	4
3	3	3	3	2	2	2	2	2
2	2	2	2	3	3	4	3	4
2	2	2	2	4	2	2	3	4
3	2	2	1	2	2	2	2	2
2	2	2	1	4	5	5	4	5
2	3	3	2	3	2	2	3	3
1	1	1	1	3	3	2	3	4
3	3	3	2	3	2	2	3	3
3	2	5	2	4	4	3	4	4
3	3	3	2	1	1	2	3	4
4	4	3	3	4	4	4	4	4
1	1	1	1	1	1	1	4	5
2	2	2	2	3	3	3	3	3
2	1	2	1	3	3	3	3	3
2	2	2	2	3	1	1	2	2
2	2	2	2	3	3	3	3	3
3	3	2	2	3	3	2	3	3
1	1	1	1	3	2	2	3	4
1	1	1	1	5	5	5	5	4
2	2	2	2	4	4	4	4	5
3	3	3	3	3	4	2	4	3
3	3	3	3	3	3	3	3	3
4	4	2	3	2	2	2	2	4
3	2	4	1	3	2	2	4	4
1	1	1	1	2	2	2	5	5
3	3	2	2	2	3	3	4	4
2	2	2	2	3	4	3	3	4
3	3	3	3	3	3	3	3	3
3	3	4	3	3	3	2	3	4
2	2	2	2	2	2	2	2	3

2	2	2	2	4	4	4	4	4
2	2	2	2	3	3	3	2	2
3	3	2	1	3	3	4	5	5
2	2	2	2	4	3	3	4	4
1	1	1	1	5	5	5	5	5
2	2	5	2	3	3	2	5	5
2	2	2	2	2	3	3	4	4
2	2	2	1	2	3	2	2	2
2	2	1	1	2	4	4	5	5
1	1	1	1	4	4	3	3	5
1	1	1	1	1	3	3	3	3
2	4	2	1	4	3	2	4	5
1	1	1	1	4	4	4	4	4
2	2	2	2	3	4	4	4	4
4	1	5	2	5	1	1	1	2
2	2	2	2	3	5	5	5	5
1	1	1	1	4	3	3	4	5
1	1	1	1	5	5	5	5	5
3	4	3	3	3	3	3	2	3
1	1	3	1	3	3	3	3	5
4	4	4	2	2	4	4	4	4
3	3	3	3	3	3	3	3	3
2	2	2	3	3	3	3	3	3
2	2	3	2	4	4	4	4	5
1	1	1	1	3	3	3	3	3
2	4	3	2	3	3	3	3	3
1	1	1	1	4	3	2	2	3
1	1	1	1	2	2	2	2	2
3	2	5	1	5	3	3	5	5
2	2	3	2	2	2	3	4	4
1	1	1	1	4	4	4	4	4
2	2	2	2	3	3	2	4	4
2	2	2	2	3	3	4	4	4
2	2	2	2	3	3	3	2	3
3	3	3	3	3	3	4	3	3
3	2	3	2	3	4	2	3	5
2	3	2	2	3	3	2	3	3
2	2	2	2	4	4	3	4	4
2	2	3	2	4	4	4	4	4
3	3	3	3	2	2	2	3	4
3	3	3	3	3	3	3	3	3
3	4	4	3	3	3	3	3	3
1	1	2	1	3	5	3	3	4
2	2	4	2	3	4	3	4	4
2	4	4	1	5	5	4	5	5
1	1	1	1	5	5	5	5	5
2	2	4	2	4	4	4	4	4
1	1	1	1	5	5	5	5	5
1	1	1	1	5	5	4	4	4

F_WE3 (1=Strongly disagree;2= Disagree;3=	JE1 (1=Strongly disagree;2= Disagree;3=	JE2 (1=Strongly disagree;2= Disagree;3=	JE3 (1=Strongly disagree;2= Disagree;3=	JE4 (1=Strongly disagree;2= Disagree;3=	JE5 (1=Strongly disagree;2= Disagree;3=	JE6 (1=Strongly disagree;2= Disagree;3=	JE7 (1=Strongly disagree;2= Disagree;3=	Totle_score _JR
1	5	5	3	3	3	3	3	48
4	3	3	3	4	3	3	3	47
3	4	4	4	2	4	2	4	46
2	3	3	3	3	3	3	3	40
4	5	5	5	5	5	5	5	50
5	4	4	5	5	5	4	5	63
5	4	4	5	5	5	5	5	47
4	4	4	4	4	4	4	4	56
4	3	3	3	3	3	3	4	43
5	5	5	5	5	5	5	5	62
3	4	4	4	5	4	5	5	48
4	4	4	5	5	5	5	4	51
5	5	5	4	4	2	2	4	51
3	4	4	4	5	5	4	5	62
3	3	3	4	4	4	4	4	52
4	4	4	4	2	4	4	4	53
4	4	4	4	3	4	3	4	47
3	4	4	4	4	4	4	4	55
5	4	4	4	5	4	5	4	68
4	3	3	3	4	4	4	4	60
4	4	4	4	4	4	4	4	58
5	5	5	5	1	5	1	5	44
4	3	4	4	4	4	4	4	56
5	4	5	5	1	5	3	5	57
3	3	3	4	3	4	3	4	50
5	5	5	5	5	5	1	5	54
4	4	4	4	5	4	5	5	58
4	2	3	2	3	3	4	2	47
3	3	3	3	3	4	4	3	49
3	5	5	4	3	5	2	4	46
4	3	4	4	3	4	3	4	45
4	4	5	4	4	4	4	5	49
2	4	4	4	4	4	4	4	46
5	4	4	4	3	3	3	3	36
5	3	5	5	5	5	5	5	57
5	5	5	5	4	1	5	5	65
5	5	5	4	4	5	4	4	44
4	3	4	4	5	4	5	4	61
4	4	4	4	4	4	3	4	50
3	5	5	5	5	5	5	5	50
5	4	4	4	5	4	4	4	58
5	4	4	3	3	3	3	3	33
3	3	3	3	3	3	3	3	55
5	5	5	3	3	3	3	3	51
4	3	4	4	4	4	4	4	59
3	4	4	3	3	4	4	4	39
5	3	4	4	4	4	4	4	53
4	4	4	4	4	4	3	4	58
3	4	3	3	3	3	3	3	52
4	3	4	3	2	3	3	4	41
5	5	5	5	1	5	1	5	63

4	4	4	4	4	4	4	4	51
4	5	5	5	5	5	1	5	51
5	3	3	3	3	4	4	4	50
4	4	4	3	3	3	3	3	34
4	4	4	4	4	4	3	3	51
4	4	4	4	4	4	4	4	51
3	3	3	3	3	3	3	3	44
4	3	4	3	5	4	3	3	51
4	4	4	2	4	4	4	3	53
4	4	4	1	2	3	3	3	41
3	3	4	4	3	4	4	4	43
3	3	3	4	4	3	4	2	46
4	5	5	5	5	5	5	5	63
4	3	4	4	4	4	4	4	46
2	3	3	4	4	4	4	4	49
4	4	5	5	4	4	5	4	57
3	4	4	4	4	4	2	4	57
4	4	4	4	4	4	4	4	42
4	4	4	4	4	4	3	4	39
3	3	3	4	4	4	4	4	52
3	3	3	3	3	4	3	4	59
4	3	5	5	4	5	5	3	43
3	4	4	4	3	3	3	3	54
3	3	4	3	3	4	3	2	38
4	3	3	3	3	3	3	3	44
5	5	5	1	4	5	4	2	27
5	3	3	4	3	4	2	4	69
3	3	3	3	3	3	3	3	50
1	3	4	4	5	5	5	3	41
5	3	3	3	3	3	3	3	43
5	3	4	4	4	4	3	4	50
4	4	4	4	4	4	4	4	58
4	4	3	3	3	4	3	3	34
4	5	5	5	5	5	3	5	66
4	4	5	3	4	4	3	4	47
5	5	5	5	5	5	5	5	75
3	3	3	4	3	3	3	4	52
5	4	4	3	4	4	3	4	40
4	3	3	4	4	2	4	4	54
5	5	5	5	4	4	3	3	48
4	4	4	4	5	1	5	5	63
5	5	5	3	3	5	3	3	34
4	5	4	5	5	5	5	5	71
4	4	4	4	5	5	5	5	57
3	4	4	3	3	3	3	3	36
4	4	4	3	3	4	3	3	33
4	4	4	4	5	4	5	4	68
4	2	2	4	3	4	5	4	59
4	4	4	4	4	4	4	4	49
4	4	5	5	4	4	4	5	52
5	4	5	5	4	5	4	5	50
2	2	3	3	2	4	2	3	36
3	3	3	3	3	3	3	3	40
4	5	4	4	5	4	5	5	67
3	3	3	3	3	4	3	3	41

4	3	3	3	3	2	3	4	44
5	4	5	5	4	4	4	5	45
4	4	5	5	4	4	4	4	49
3	3	3	3	3	4	3	4	47
4	4	4	4	4	5	4	5	45
5	5	5	5	5	5	5	5	47
3	3	3	3	3	3	3	3	47
3	3	3	3	3	4	3	4	32
5	4	5	5	4	5	4	3	48
3	2	4	4	2	4	2	4	44
3	3	3	3	3	3	3	3	51
4	4	4	4	4	4	4	4	38
3	5	4	4	5	4	5	4	53
3	3	3	3	3	3	3	3	42
5	5	5	5	5	5	5	5	57
3	4	4	4	4	4	4	3	34
4	3	3	3	3	4	3	4	50
4	5	4	4	5	4	5	4	41
3	3	3	3	3	3	3	3	43
3	4	4	4	4	4	4	5	50
4	4	4	4	4	4	4	4	47
3	5	4	4	5	4	5	5	63
5	5	5	5	5	5	5	5	41
4	3	3	3	3	4	3	4	43
4	4	3	3	4	2	4	3	45
4	5	5	5	5	5	5	5	65
4	5	5	5	5	5	5	5	54
2	3	3	3	3	3	3	3	48
4	4	4	4	4	4	4	4	46
4	5	5	5	5	5	5	5	69
3	3	3	3	3	3	3	3	45
3	3	3	3	3	4	3	5	35
3	3	3	3	3	4	3	3	36
4	2	4	4	2	4	2	4	35
3	5	5	5	5	4	5	4	47
3	1	3	3	1	4	1	3	31
3	4	3	3	4	2	4	3	49
4	4	4	4	4	5	4	5	44
5	4	5	5	4	5	4	5	32
5	3	3	3	3	5	3	3	46
3	2	1	1	2	3	2	3	37
4	4	4	4	4	3	4	4	53
1	5	5	5	5	5	5	5	61
5	5	5	5	5	5	5	5	70
4	4	4	4	4	4	4	3	49
5	3	3	3	3	3	3	3	37
5	5	4	4	5	4	5	5	54
5	3	3	3	3	4	3	3	38
3	5	4	4	5	5	5	5	55
5	5	5	5	5	5	5	5	67
3	4	4	4	4	4	4	4	56
5	5	5	5	5	5	5	5	69
4	3	4	4	3	5	3	4	47
3	5	4	4	5	4	5	3	49
3	3	3	3	3	3	3	3	45

2	2	2	2	2	3	2	3	31
3	4	4	4	4	4	4	4	49
4	5	4	4	5	4	5	4	61
4	5	4	4	5	4	5	4	66
5	5	4	4	5	5	5	5	55
3	4	4	4	4	4	4	4	52
3	4	3	3	4	3	4	3	40
4	4	4	4	4	4	4	4	51
2	3	3	3	3	5	3	3	48
4	3	4	4	3	4	3	4	55
4	5	4	4	5	4	5	4	51
4	4	4	4	4	5	4	4	52
4	4	3	3	4	2	4	3	46
4	2	2	2	2	3	2	3	45
4	4	4	4	4	4	4	4	47
3	3	3	3	3	3	3	4	45
3	4	3	3	4	2	4	4	60
4	2	4	4	2	3	2	3	49
5	3	3	3	3	3	3	3	37
4	5	4	4	5	4	5	4	52
3	5	5	5	5	4	5	5	55
3	4	4	4	4	4	4	4	57
3	5	5	5	5	5	5	5	59
4	2	4	4	2	4	2	4	65
4	5	3	3	5	5	5	3	53
5	5	5	5	5	5	5	4	63
1	5	5	5	5	5	5	5	63
4	5	5	5	5	5	5	5	64
4	5	4	4	5	4	5	4	73
4	4	4	4	4	4	4	4	57
5	5	4	4	5	5	5	4	48
4	4	4	4	4	4	4	4	63
3	3	4	4	3	4	3	4	48
3	4	3	3	4	4	4	3	45
5	5	5	5	5	5	5	5	64
5	5	5	5	5	5	5	5	65
3	2	4	4	2	4	2	3	49
5	5	5	5	5	5	5	5	69
4	5	4	4	5	4	5	4	49
3	3	3	3	4	4	4	4	46
5	4	4	4	5	4	5	4	64
4	4	4	4	4	4	4	4	54
3	3	3	3	4	4	3	4	40
3	3	3	3	3	3	3	4	53
4	4	4	4	4	4	5	4	55
4	4	4	4	3	4	4	5	49
4	4	4	4	4	4	4	4	43
3	3	3	3	3	2	3	3	54
3	3	4	3	3	4	4	3	43
3	3	3	4	4	4	3	4	57
3	3	3	3	3	3	3	3	51
3	3	3	3	3	3	3	3	47
4	3	4	4	3	4	4	3	54
4	5	5	5	4	4	4	5	48
3	4	4	3	3	3	3	3	49

4	3	4	4	4	4	3	4	49
5	4	5	5	5	5	5	5	63
1	3	3	1	2	4	3	3	34
4	4	4	4	4	4	3	4	43
3	3	3	3	3	3	3	3	41
5	4	4	4	5	4	4	5	58
5	3	3	1	2	3	3	3	29
2	5	4	4	5	5	5	5	41
3	4	4	4	4	4	4	4	44
4	3	4	4	4	4	4	4	69
3	3	4	4	4	3	3	3	49
3	4	4	4	4	4	4	4	47
4	4	4	4	3	4	4	4	51
3	3	3	3	4	4	4	3	52
4	4	5	4	5	5	5	5	54
3	4	4	4	4	4	3	4	42
4	3	3	4	5	4	4	4	59
4	3	3	3	3	3	3	3	35
3	4	4	4	4	4	4	4	46
3	5	5	5	3	5	1	5	51
4	4	4	4	4	4	4	4	58
4	5	5	5	5	5	5	5	59
5	5	5	5	4	5	4	5	47
3	3	3	3	3	3	3	3	52
4	4	4	2	2	4	4	4	45
5	1	1	1	1	3	1	3	44
5	4	4	4	4	4	4	4	53
1	3	4	4	5	5	5	5	49
5	5	5	5	5	5	5	5	52
3	3	3	3	3	4	4	4	42
2	2	2	4	4	2	4	4	59
5	4	4	4	4	5	4	5	46
3	4	4	4	4	4	4	5	46
4	5	5	5	5	5	5	5	50
4	4	4	4	4	4	4	4	43
4	4	4	4	5	4	4	5	69
3	4	4	4	2	4	2	4	47
3	3	3	3	3	3	3	3	49
3	3	3	4	4	3	4	4	54
4	5	5	5	3	5	5	5	58
4	4	4	4	2	4	2	4	37
5	3	2	3	3	4	3	4	43
4	4	4	4	4	4	3	4	53
5	4	5	5	4	5	5	5	54
5	5	5	5	5	5	5	5	65
4	5	5	5	1	5	1	5	59
4	3	3	4	3	4	2	4	54
4	5	4	4	4	4	4	5	48
3	3	3	3	3	4	4	4	43
5	5	3	5	4	5	5	5	64
4	4	4	4	4	4	4	5	61
4	4	4	4	4	4	4	4	52
4	4	4	4	4	4	4	4	71
3	5	5	5	5	5	3	5	55
3	4	4	4	4	4	2	4	56

4	4	4	4	4	4	4	4	46
5	5	5	5	3	5	3	5	49
3	3	4	4	4	4	4	2	46
3	3	5	5	5	5	5	5	55
4	4	4	4	5	5	5	5	56
2	3	3	3	3	3	3	3	42
4	4	4	5	5	5	5	5	51
5	4	4	4	4	5	4	5	59
4	3	3	5	5	5	4	5	60
4	4	4	4	4	4	2	4	42
5	4	3	4	4	4	3	3	44
4	4	4	4	4	4	4	4	55
4	4	4	4	4	4	4	4	53
3	4	4	4	4	4	4	4	46
4	5	5	5	5	5	5	5	54
3	3	3	3	3	3	3	3	38
4	4	4	4	2	5	2	5	49
3	4	3	5	5	4	5	5	47
3	3	3	3	3	3	3	3	52
4	4	4	3	3	4	3	3	45
4	3	3	3	3	3	3	3	53
3	3	4	5	5	5	5	5	68
4	4	4	5	5	4	4	4	61
1	5	5	5	5	5	5	5	68
3	3	3	3	3	3	3	3	50
3	4	4	4	4	4	3	3	47
3	4	4	4	5	4	5	4	54
3	3	2	5	5	5	5	5	69
5	5	5	5	5	5	5	4	54
4	3	4	3	4	3	1	2	42
5	1	4	1	4	2	3	3	57
4	4	4	4	4	3	2	3	58
4	4	5	4	5	4	4	4	63
2	1	2	1	2	3	1	1	45
4	3	3	3	3	3	3	3	56
4	3	3	3	3	3	3	3	56
4	2	2	2	2	3	2	2	48
5	2	3	2	3	3	2	3	38
4	1	2	1	2	3	2	3	60
3	5	5	5	5	3	1	1	63
5	4	4	4	4	4	3	3	69
4	2	2	2	2	3	2	3	58
3	2	3	2	3	2	1	2	44
4	2	2	2	2	2	3	4	43
3	3	3	3	3	4	3	3	53
5	3	5	3	5	4	1	1	52
4	1	2	1	2	2	1	2	47
1	4	5	4	5	4	5	5	63
5	4	4	4	4	3	1	2	53
4	4	5	4	5	5	3	3	63
5	3	3	3	3	4	2	3	64
1	1	3	1	3	3	1	1	36
5	1	1	1	1	1	1	1	39
4	5	4	5	4	5	5	5	68
5	3	3	3	3	2	2	2	45

3	3	3	3	3	3	1	1	49
4	3	3	3	3	3	2	3	49
4	2	2	2	2	1	2	3	53
4	1	2	1	2	4	1	1	52
4	2	2	2	2	2	2	2	51
4	3	4	3	4	3	1	3	50
5	4	4	4	4	4	3	4	56
3	2	2	2	2	2	2	2	43
4	3	3	3	3	3	2	2	50
5	3	4	3	4	2	3	3	58
3	4	4	4	4	4	1	1	56
4	2	3	2	3	2	2	2	52
3	3	4	3	4	3	2	3	44
5	4	4	4	4	4	2	2	54
3	3	4	3	4	3	2	3	61
4	2	3	2	3	2	1	1	37
4	4	4	4	4	4	4	4	74
4	2	3	2	3	1	1	1	46
3	4	4	4	4	4	3	3	59
4	3	3	3	3	3	3	4	56
4	1	4	1	4	4	2	2	48
4	5	5	5	5	4	3	3	68
5	4	5	4	5	5	3	3	67
4	3	3	3	3	3	1	2	57
1	2	3	2	3	2	1	2	37
4	4	4	4	4	4	3	3	50
1	2	3	2	3	3	3	3	47
4	2	4	2	4	3	2	4	60
3	3	3	3	3	3	1	3	45
5	4	4	4	4	1	1	2	67
4	4	4	4	4	4	3	4	74
5	4	5	4	5	4	4	5	74
2	3	4	3	4	4	3	3	50
3	3	3	3	3	2	3	3	47
3	5	5	5	5	3	2	3	48
4	3	3	3	3	3	4	2	49
5	5	5	5	5	5	5	5	69
3	3	4	3	4	4	3	3	66
3	4	5	4	5	2	2	4	59
4	2	2	2	2	2	2	2	54
4	4	5	4	5	4	2	3	60
4	4	5	4	5	4	3	3	57
4	2	3	2	3	4	3	3	67
4	1	3	1	3	2	1	3	48
4	4	4	4	4	2	1	1	52
2	1	1	1	1	1	3	2	56
4	4	4	4	4	1	1	2	51
3	3	3	3	3	3	1	2	41
5	1	4	1	4	1	1	3	54
5	3	4	3	4	5	2	3	61
4	4	2	4	2	3	2	2	48
3	4	4	4	4	3	1	2	53
3	1	1	1	1	1	1	1	55
3	1	3	1	3	3	1	1	57
5	4	5	4	5	3	2	2	60

5	2	2	2	2	3	1	1	54
4	4	5	4	5	3	3	3	60
3	2	3	2	3	1	1	1	41
4	2	4	2	4	3	2	2	49
4	3	3	3	3	2	1	1	41
4	1	2	1	2	1	1	1	37
4	4	4	4	4	3	1	1	42
4	1	1	1	1	1	1	2	56
4	2	3	2	3	3	1	3	46
3	2	2	2	2	2	2	2	45
3	1	1	1	1	2	2	2	48
2	1	1	1	1	1	1	1	36
5	1	1	1	1	1	1	1	33
3	5	5	5	5	4	2	3	65
4	3	4	3	4	4	1	3	59
3	2	3	2	3	2	1	1	44
5	5	5	5	5	4	1	4	66
5	1	3	1	3	1	1	1	55
3	2	2	2	2	2	2	1	43
3	2	2	2	2	2	5	5	51
3	3	4	3	4	4	3	4	57
4	2	3	2	3	4	3	4	51
5	3	3	3	3	2	1	2	46
5	4	5	4	5	4	3	3	52
5	2	3	2	3	1	1	3	68
2	3	4	3	4	2	1	1	47
4	2	3	2	3	2	4	4	53
1	5	4	5	4	4	4	4	51
3	3	3	3	3	3	3	3	45
3	3	5	3	5	4	1	3	60
2	2	2	2	2	2	2	2	37
3	2	4	2	4	1	1	1	40
4	3	3	3	3	4	3	3	50
5	1	2	1	2	1	3	3	65
4	2	3	2	3	5	3	3	56
3	5	4	5	4	3	2	2	59
3	1	4	1	4	3	3	3	48
5	4	5	4	5	3	2	3	66
4	2	3	2	3	3	2	3	47
4	4	3	4	3	5	3	3	52
4	3	4	3	4	3	1	3	58
3	3	3	3	3	3	1	2	47
3	2	3	2	3	3	2	2	45
5	3	4	3	4	4	2	4	60
4	2	2	2	2	2	1	2	56
4	4	4	4	4	3	3	2	52
1	1	4	1	4	2	1	1	52
4	2	5	2	5	4	4	5	64
4	1	2	1	2	2	1	3	47
5	4	4	4	4	3	1	1	44
5	3	3	3	3	3	3	4	54
4	1	3	1	3	1	1	1	49
4	5	5	5	5	5	4	4	67
4	2	2	2	2	2	3	3	52
5	4	4	4	4	4	1	1	48

5	1	3	1	3	1	1	1	48
5	4	4	4	4	5	4	4	62
3	3	4	3	4	3	3	3	48
4	3	4	3	4	1	1	1	45
4	4	3	4	3	3	3	3	57
3	3	1	3	1	2	1	2	42
4	2	3	2	3	2	1	1	52
4	4	4	4	4	4	2	4	48
3	1	1	1	1	2	1	1	54
3	2	2	2	2	2	2	2	41
5	3	4	3	4	3	1	2	56
4	3	4	3	4	2	2	2	51
4	3	3	3	3	3	1	3	49
4	3	5	3	5	1	2	2	54
3	3	4	3	4	3	1	2	57
4	4	4	4	4	3	3	4	58
3	2	2	2	2	3	1	2	51
3	2	2	2	2	2	2	4	61
5	4	4	4	4	3	3	3	63
2	3	5	3	5	3	1	3	50
3	2	3	2	3	2	1	2	46
3	4	4	4	4	3	1	3	49
3	3	4	3	4	2	2	3	47
3	3	3	3	3	2	3	3	52
4	1	1	1	1	2	1	2	50
4	3	2	3	2	3	1	2	49
3	2	2	2	2	3	1	1	52
5	1	1	1	1	1	1	1	45
3	3	5	3	5	2	2	3	46
3	3	3	3	3	3	3	3	50
5	1	1	1	1	2	1	1	42
3	1	1	1	1	1	1	1	47
3	1	1	1	1	2	1	3	48
3	3	3	3	3	3	3	3	45
4	1	3	1	3	1	1	3	43
4	3	2	3	2	2	2	2	42
4	2	2	2	2	2	1	1	54
3	2	3	2	3	3	3	4	51
3	2	3	2	3	2	1	3	46
3	2	3	2	3	2	1	1	40
3	2	5	2	5	3	3	3	58
4	3	4	3	4	2	1	1	47
4	4	4	4	4	4	3	4	60
3	3	3	3	3	2	2	2	42
3	2	2	2	2	2	1	1	39
4	4	4	4	4	4	3	3	55
5	3	4	3	4	3	1	1	50
5	2	3	2	3	2	1	2	44
5	4	4	4	4	2	1	2	45
4	3	3	3	3	3	2	2	56
3	3	3	3	3	3	3	3	45
5	3	3	3	3	3	3	3	45
4	3	3	3	3	3	4	5	59
3	1	1	1	1	1	1	1	42
3	3	4	3	4	2	2	2	58

3	3	4	3	4	4	3	3	49
2	2	3	2	3	2	2	3	48
3	2	4	2	4	2	4	3	43
4	5	5	5	5	5	5	5	59
4	2	3	2	3	2	1	1	47
4	4	5	4	5	5	3	5	68
3	4	4	4	4	3	1	3	52
5	3	3	3	3	1	1	1	32
4	1	1	1	1	1	1	1	47
4	4	4	4	4	4	3	3	64
5	5	5	5	5	4	5	5	65
3	2	2	2	2	2	1	1	37
4	3	4	3	4	2	2	2	43
3	2	2	2	2	2	1	1	45
3	3	3	3	3	3	2	3	58
3	3	5	3	5	3	2	2	60
3	3	3	3	3	3	3	3	48
4	3	4	3	4	2	1	3	51
3	2	4	2	4	3	2	3	48
4	3	4	3	4	3	4	4	53
5	2	3	2	3	4	1	2	59
4	3	4	3	4	3	1	1	54
3	2	2	2	2	3	2	2	38
4	4	4	4	4	3	3	3	58
3	3	4	3	4	3	2	3	47
4	3	2	3	2	2	1	3	44
3	3	4	3	4	3	3	3	58
4	3	4	3	4	4	1	3	53
3	3	3	3	3	4	3	3	47
3	2	2	2	2	2	1	2	47
5	5	5	5	5	5	5	5	61
5	2	4	2	4	1	2	5	53
4	4	4	4	4	4	4	4	46
3	2	3	2	3	2	1	1	55
4	3	4	3	4	3	1	3	50
4	2	4	2	4	3	2	4	68
3	3	3	3	3	2	1	3	59
3	1	2	1	2	2	4	2	43
4	2	2	2	2	2	1	2	56
4	1	1	1	1	4	1	4	48
3	2	3	2	3	2	2	2	49
3	2	4	2	4	3	1	1	42
2	1	1	1	1	1	1	1	44
4	5	5	5	5	1	1	1	45
4	2	2	2	2	1	1	1	50
5	4	4	4	4	4	3	4	67
4	3	3	3	3	3	2	3	56
3	2	3	2	3	4	2	3	57
4	1	2	1	2	2	1	1	38
4	3	3	3	3	2	1	2	56
4	5	5	5	5	3	2	2	35
5	4	5	4	5	4	2	3	51
4	2	4	2	4	3	2	3	48
4	1	2	1	2	2	1	1	41
5	3	4	3	4	2	3	3	47

4	4	4	4	4	3	3	1	48
4	4	4	4	4	3	1	3	46
4	3	4	3	4	4	3	3	50
5	4	4	4	4	4	2	3	65
4	3	4	3	4	3	2	3	44
5	1	3	1	3	2	2	4	56
4	2	4	2	4	3	1	2	46
4	1	3	1	3	1	1	2	40
3	3	3	3	3	3	3	3	55
4	5	5	5	5	5	1	3	65
5	2	4	2	4	3	2	2	44
4	2	3	2	3	3	2	3	55
5	4	2	4	2	3	2	3	60
3	1	2	1	2	2	2	3	57
3	2	3	2	3	1	1	1	49
3	3	4	3	4	3	3	3	56
2	3	2	3	2	3	3	3	48
4	3	4	3	4	3	3	3	57
5	4	3	4	3	4	1	1	38
4	1	1	1	1	1	1	1	53
3	3	3	3	3	3	3	3	45
3	2	2	2	2	3	5	4	48
5	4	4	4	4	4	1	3	74
4	1	4	1	4	3	1	4	44
4	1	2	1	2	2	1	2	46
4	3	3	3	3	3	1	3	36
5	3	4	3	4	3	1	3	67
3	2	2	2	2	2	1	1	45
5	1	2	1	2	1	1	3	59
5	2	3	2	3	2	1	2	71
3	3	4	3	4	4	2	2	52
4	1	3	1	3	4	1	1	57
5	2	2	2	2	2	2	2	47
4	2	4	2	4	4	4	4	67
3	3	3	3	3	2	2	3	44
5	1	3	1	3	1	1	2	30
4	2	3	2	3	4	2	3	40
1	2	2	2	2	2	2	1	43
3	2	3	2	3	3	1	1	46
3	3	4	3	4	3	2	4	51
4	4	3	4	3	2	2	2	42
4	1	3	1	3	2	1	1	57
4	1	3	1	3	2	2	3	52
5	4	5	4	5	4	4	4	65
3	4	4	4	4	3	2	3	63
3	3	2	3	2	3	2	3	46
3	4	4	4	4	2	1	1	38
3	3	3	3	3	3	3	3	47
4	3	5	3	5	4	4	4	52
3	4	3	4	3	3	1	2	40
4	2	3	2	3	3	2	3	51
3	3	4	3	4	4	1	2	48
5	1	1	1	1	1	1	1	43
3	2	3	2	3	3	3	4	43
4	3	2	5	2	5	4	4	56

3	3	3	1	2	1	4	4	51
4	4	3	3	2	3	4	4	66
5	4	1	2	5	2	5	3	48
4	4	4	2	4	2	4	4	56
4	3	2	1	2	1	3	3	51
2	4	5	3	4	3	3	3	36
3	5	5	5	5	5	4	2	50
3	4	3	3	3	3	4	4	49
4	1	1	1	1	1	4	4	69
4	3	2	1	3	1	5	5	61
3	3	3	3	3	3	4	4	53
4	3	4	2	3	2	3	3	55
3	3	3	2	2	2	3	4	60
4	3	5	2	5	2	3	4	52
3	4	5	4	3	4	4	4	58
5	3	2	1	2	1	5	5	63
4	4	3	3	3	2	5	5	48
3	2	2	2	2	2	3	3	56
3	4	5	5	5	4	5	5	54
5	3	5	3	5	4	3	3	46
4	4	3	2	3	2	4	4	50
4	3	2	2	2	2	4	4	53
4	3	2	2	2	1	3	3	54
5	3	4	2	3	1	5	5	60
4	3	2	3	3	3	3	4	56
3	3	4	1	4	4	3	3	54
3	3	2	2	2	1	3	3	57
3	3	2	2	2	2	3	3	55
4	4	4	3	5	4	4	4	42
4	2	3	2	3	3	3	3	50
3	2	2	1	1	1	4	4	64
3	3	3	1	3	2	3	3	51
3	3	2	2	2	2	4	4	51
4	4	4	2	3	3	4	4	51
4	4	5	5	5	4	5	4	41
3	4	5	3	4	4	4	4	48
4	2	3	1	2	1	3	4	62
4	3	3	4	3	2	3	4	61
5	4	4	3	4	4	5	5	56
5	4	3	2	3	4	4	4	54
4	3	2	2	3	3	3	3	50
5	4	4	1	3	4	5	5	61
3	4	4	2	3	2	2	3	48
4	3	3	5	2	4	4	3	47
5	2	2	1	2	2	5	5	63
3	3	3	2	2	2	4	3	54
4	3	4	2	3	5	3	4	55
3	3	2	2	3	3	4	3	54
5	4	4	4	4	2	3	3	45
3	5	4	3	4	4	4	4	50
1	3	2	2	3	3	5	5	67
5	4	4	4	4	3	4	5	54
5	5	1	1	3	1	5	5	75
5	4	5	3	3	3	4	4	49
4	4	2	3	4	4	4	4	54

4	4	4	4	4	4	4	4	50
5	3	1	1	1	1	5	5	73
3	3	1	1	3	2	3	3	41
3	3	4	3	3	4	2	3	47
4	4	5	3	5	2	3	4	60
4	5	5	5	5	3	5	5	61
4	4	5	3	4	4	4	4	55
3	4	4	4	4	3	3	3	43
3	4	4	4	4	2	3	3	54
5	3	3	3	3	2	3	3	55
3	5	5	4	4	3	3	3	45
4	4	4	4	4	2	5	5	38
1	5	5	3	3	3	4	5	50
3	5	5	3	5	4	5	5	36
4	3	2	2	3	2	4	4	55
4	3	2	2	3	2	4	4	56
4	4	4	4	4	2	3	4	52
5	2	1	1	1	1	5	5	75
3	3	2	1	3	3	5	1	57
4	5	3	3	3	3	5	4	52
4	2	1	1	3	1	4	4	62
3	3	3	2	2	4	4	4	53
3	4	4	1	3	2	5	5	56
4	5	3	4	4	4	4	5	48
3	3	3	1	3	4	4	3	51
3	4	4	1	3	3	3	3	42
3	3	1	1	2	2	3	3	67
3	4	5	2	5	3	3	3	41
3	5	3	3	3	3	3	3	47
3	4	3	4	4	4	3	3	64
4	3	3	2	2	2	4	4	59
3	5	5	3	5	5	4	5	47
5	4	4	3	4	3	3	3	42
3	3	3	3	3	3	3	2	55
5	5	5	5	5	5	3	3	50
3	4	4	4	3	5	3	3	42
4	4	4	4	4	3	2	4	42
4	3	3	2	3	3	4	4	52
4	4	3	2	3	3	3	3	54
5	4	3	2	3	2	5	4	59
4	3	2	1	3	2	4	4	60
5	3	3	2	2	2	5	5	75
4	4	4	2	4	4	4	3	54
4	5	4	5	5	4	5	5	49
4	3	3	1	3	2	3	3	50
4	5	4	3	4	2	3	3	36
4	5	3	2	3	3	3	3	43
3	4	4	3	4	3	4	4	58
5	5	5	5	3	3	5	5	64
3	3	3	1	3	1	3	3	64
4	5	4	4	3	2	4	4	50
3	5	2	1	3	1	4	4	50
5	5	5	3	4	3	5	4	41
4	5	5	5	5	4	3	3	46
4	3	3	2	2	2	4	4	52

4	3	5	4	4	2	5	5	62
4	3	3	2	3	4	4	5	57
3	5	5	4	5	3	4	4	38
4	4	5	3	5	3	4	4	46
4	4	3	2	3	2	4	4	48
4	3	3	2	3	4	5	4	59
3	3	2	2	3	1	4	3	61
5	3	4	2	3	1	5	5	70
5	4	5	5	5	3	4	4	43
3	2	3	1	2	2	3	3	62
2	5	5	4	5	4	3	3	27
3	4	4	4	4	3	3	3	52
4	4	3	1	4	2	3	3	43
4	3	3	1	3	4	3	3	50
3	3	2	2	3	4	4	3	51
1	5	5	4	5	2	3	3	52
3	3	2	1	2	2	3	3	50
4	3	3	2	3	2	5	4	67
5	3	4	3	3	2	4	5	57
4	5	4	3	4	5	1	4	38
4	5	3	1	3	3	3	4	57
4	3	4	4	3	3	4	4	51
4	3	3	3	2	2	4	4	63
5	4	3	3	3	1	5	5	56
1	4	4	4	5	3	3	2	33
4	5	4	1	3	1	5	3	53
4	3	3	3	3	3	4	4	51
3	3	4	3	4	3	3	3	45
2	4	4	3	5	3	5	4	46
4	4	4	4	3	2	4	4	57
2	4	4	4	4	4	3	3	37
5	3	1	1	1	1	5	4	63
4	5	5	4	4	3	5	5	60
3	4	4	3	4	3	3	3	44
3	4	4	3	3	4	4	4	51
4	3	4	1	3	3	5	5	54
4	4	4	3	5	4	4	5	49
5	3	4	1	3	4	5	5	63
4	5	4	4	5	3	4	3	44
2	5	4	3	5	5	3	3	41
5	4	4	4	3	2	5	5	65
4	3	4	2	3	1	3	2	51
4	5	4	2	4	4	4	4	50
3	3	3	2	3	3	4	4	46
4	4	4	3	3	2	3	3	37
4	5	5	5	5	2	3	3	36
4	5	4	4	4	3	4	5	52
5	3	3	3	3	3	4	3	48
3	3	3	3	5	3	3	3	42
5	3	3	4	4	2	3	3	49
5	4	4	5	4	4	3	4	39
4	4	4	2	3	2	4	4	49
5	3	3	2	3	2	5	5	60
3	4	2	3	4	4	3	3	37
3	4	3	3	3	2	5	5	51

5	3	4	2	3	5	5	5	56
2	3	5	4	4	4	3	2	40
2	5	5	2	2	2	4	4	48
4	4	4	5	4	4	3	3	40
3	3	4	2	4	4	4	4	55
2	4	4	4	4	4	5	4	51
2	5	4	4	4	4	1	4	50
4	5	5	4	5	5	2	4	38
4	3	3	3	3	3	3	4	53
4	4	5	3	5	5	3	3	51
2	4	4	3	4	4	4	4	36
3	3	3	3	3	3	4	3	48
4	4	4	4	4	4	4	4	47
3	4	5	4	4	4	3	3	46
5	2	2	1	2	2	5	5	60
5	3	4	2	3	3	4	4	46
5	4	3	1	3	3	4	4	46
4	4	4	3	3	3	3	3	36
4	4	4	3	3	3	4	5	47
2	3	3	2	3	3	3	5	41
4	3	5	3	4	4	4	4	59
3	4	5	5	4	4	3	3	45
4	4	4	4	4	4	4	4	42
3	4	4	3	4	4	3	3	45
5	4	3	2	3	3	5	5	71
3	5	4	4	5	5	3	3	32
4	4	4	3	5	5	3	3	45
3	4	4	4	4	4	3	3	46
5	4	3	2	3	3	4	4	58
4	2	2	2	2	2	4	4	59
5	1	1	1	1	1	5	5	67
4	3	4	1	3	3	1	4	63
4	3	3	5	3	3	5	5	68
4	4	4	3	3	3	4	4	54
3	2	2	2	3	3	1	3	40
3	2	3	2	2	4	4	3	46
5	3	1	1	1	5	4	4	53
4	3	3	3	3	4	4	4	54
4	4	3	3	2	2	4	4	50
4	1	2	2	2	2	3	3	40
3	3	3	3	3	5	3	3	47
4	3	4	4	4	4	4	4	57
3	2	2	2	2	3	3	3	46
3	3	3	2	2	5	4	5	51
5	2	3	3	3	5	4	5	58
3	4	3	2	4	4	3	4	56
4	2	2	2	3	4	4	4	51
4	2	3	3	3	4	3	2	50
4	3	2	3	4	5	5	5	52
4	3	4	4	4	4	5	5	52
2	1	1	1	1	1	3	3	35
4	3	3	3	3	5	5	4	57
4	5	5	5	5	5	4	5	70
3	3	3	3	3	3	4	4	59
4	5	5	5	5	5	4	4	50

4	2	2	2	3	4	5	4	62
3	2	2	3	2	4	4	4	45
4	2	2	2	2	4	3	4	46
4	2	2	2	2	4	3	3	44
5	5	1	1	2	5	2	3	45
4	1	1	1	1	1	3	3	41
3	3	3	3	3	3	4	4	59
4	3	2	2	3	3	2	4	45
3	1	1	1	1	1	3	3	42
4	3	1	1	3	5	4	4	46
3	2	3	3	3	3	3	3	54
3	2	3	2	2	3	3	4	41
2	3	4	4	4	4	4	4	47
5	2	1	1	1	5	4	4	39
5	2	2	3	3	2	4	4	40
3	2	2	2	2	3	3	3	49
3	3	2	2	2	2	3	3	47
5	5	5	5	5	5	5	5	63
3	4	4	4	4	4	4	4	49
3	3	2	2	2	3	3	3	38
3	1	1	1	1	3	3	3	44
4	4	2	3	4	4	5	5	55
4	3	2	2	2	2	3	3	52
4	2	2	2	2	3	3	3	49
4	1	1	1	2	2	3	5	50
4	4	3	3	4	5	5	4	59
4	3	3	4	3	4	4	4	66
4	3	4	4	3	5	5	4	49
4	4	4	4	4	4	2	4	58
4	4	4	4	4	4	4	4	75
4	5	5	5	5	5	5	5	74
3	3	3	3	3	3	4	4	53
3	3	2	2	2	2	3	3	48
4	2	1	1	2	4	4	4	39
4	2	2	2	2	4	4	4	52
3	2	2	2	2	2	3	3	38
3	3	3	5	3	3	2	4	71
5	1	1	1	3	5	2	4	37
4	2	1	2	1	3	4	4	48
3	2	1	1	3	3	3	4	54
2	2	3	2	2	4	4	4	40
3	4	4	4	3	3	5	5	73
4	3	1	1	1	4	3	3	46
3	3	2	2	3	4	4	3	59
4	3	3	3	3	3	5	5	51
4	2	3	3	2	3	3	3	44
4	3	2	4	5	5	4	4	54
5	4	4	4	3	4	4	5	75
4	4	4	3	4	4	5	4	58
4	1	1	2	1	1	3	3	36
4	1	2	1	1	3	4	5	55
4	1	1	1	1	5	3	4	47
4	3	4	4	4	4	5	4	60
3	2	2	2	2	3	3	3	53
3	3	2	2	4	5	5	5	56

4	5	5	5	5	5	2	4	52
3	3	3	2	2	4	3	4	50
4	2	2	2	2	3	4	4	40
3	4	4	4	4	4	3	4	49
3	2	3	3	3	3	3	3	41
3	2	2	1	2	4	4	3	50
5	2	3	3	4	5	5	5	60
3	2	3	3	3	3	4	4	47
4	2	4	3	4	4	5	3	48
3	3	3	3	3	3	3	3	43
4	4	4	4	4	5	5	5	70
3	3	4	4	4	3	4	4	51
2	1	1	1	1	1	1	4	34
3	1	1	1	1	3	3	3	45
4	2	3	3	4	5	2	3	61
5	1	3	5	5	5	4	5	55
3	2	2	2	2	2	4	4	49
3	2	3	1	1	3	4	4	52
5	5	5	5	5	5	5	5	75
5	2	2	2	3	5	4	4	54
3	3	3	3	3	4	3	4	65
2	1	1	1	1	5	5	3	36
3	3	1	1	1	1	3	4	38
4	3	3	3	3	4	5	5	54
5	1	3	3	3	5	3	4	47
4	2	3	4	4	4	4	3	63
3	4	3	3	4	4	4	4	60
4	2	2	2	2	4	3	3	46
4	2	1	2	3	4	4	4	43
5	3	2	2	3	3	4	5	47
4	2	2	2	2	3	3	3	41
3	3	3	2	2	2	3	5	53
3	1	1	1	1	2	4	5	46
5	1	1	1	1	2	3	3	48
4	2	2	2	2	2	4	4	50
3	3	3	3	3	4	3	3	46
5	5	5	5	5	5	5	4	63
5	1	1	1	1	3	5	4	42
5	3	3	3	3	5	5	5	60
3	3	4	3	3	4	5	5	48
3	2	3	3	3	2	3	3	48
3	3	4	4	2	3	4	4	48
4	2	2	2	2	4	4	5	61
3	3	3	3	3	3	4	4	54
3	5	2	3	1	4	3	3	52
4	4	2	2	2	4	4	4	56
4	2	2	2	2	2	4	3	45
4	3	2	2	2	2	4	5	42
4	2	2	3	3	3	3	3	31
3	2	2	2	2	3	4	4	52
3	3	2	3	3	3	4	4	54
4	4	2	2	3	4	4	3	51
5	2	3	3	2	3	3	3	42
5	5	3	3	3	5	5	5	61
5	3	3	3	3	5	4	5	50

1	2	1	2	1	5	4	3	44
4	3	3	3	3	4	4	4	54
4	3	3	3	3	3	3	3	52
4	1	2	2	1	2	4	4	48
4	4	3	3	3	4	5	4	58
5	4	4	4	4	5	3	3	43
3	3	2	2	3	3	3	3	41
4	2	2	2	2	2	3	4	50
4	2	2	2	2	2	3	4	49
5	5	5	5	5	5	5	5	75
3	3	3	3	3	3	4	4	47
4	4	4	4	4	4	2	4	59
3	3	3	3	3	3	3	3	54
5	3	2	4	4	4	5	5	62
4	2	2	2	2	2	4	4	56
3	2	2	2	1	2	2	3	54
5	4	3	4	4	5	3	5	45
5	2	1	2	4	4	3	5	48
5	3	3	3	3	5	4	4	55
5	3	3	3	3	3	4	4	57
4	2	3	3	3	4	4	4	49
3	2	2	1	2	5	3	3	41
4	3	1	2	2	4	4	4	54
4	2	3	3	2	5	4	5	62
4	2	2	1	2	4	5	5	50
2	3	2	3	4	4	4	4	50
4	2	3	3	3	3	3	3	46
5	3	2	3	4	4	4	4	56
5	4	4	4	5	5	4	4	61
4	4	4	4	4	4	4	4	61
3	2	3	3	3	3	3	4	46
3	3	2	3	3	3	3	3	56
4	2	2	2	2	4	3	3	43
4	3	1	1	2	4	3	3	40
2	3	2	2	2	5	4	4	48
4	3	3	3	3	4	2	3	38
3	3	2	2	2	2	4	4	53
4	4	2	2	1	1	4	4	50
4	2	2	2	1	3	4	4	51
4	2	2	2	3	3	5	4	56
3	4	3	3	4	3	4	4	64
4	2	4	4	3	4	5	5	56
4	2	1	2	3	4	5	3	47
5	5	5	5	5	5	5	5	75
4	3	2	2	2	4	4	4	56
3	5	5	3	4	5	4	4	60
3	2	3	3	3	3	4	4	49
5	1	1	1	1	4	4	5	40
4	1	1	1	1	4	3	3	39
4	3	4	4	4	4	4	4	73
4	2	2	2	2	4	4	4	58
4	3	3	3	3	1	4	4	51
5	3	2	3	2	4	5	4	54
4	3	2	3	4	3	5	5	60
4	1	1	1	1	1	1	4	33

4	1	1	1	1	4	3	3	38
4	3	3	3	3	3	3	3	41
4	4	2	3	3	4	3	3	44
4	2	2	2	2	3	4	3	50
3	3	2	3	3	4	3	3	56
1	2	3	3	2	3	4	4	43
3	1	1	1	1	1	4	3	40
3	4	4	3	4	3	3	3	45
4	3	2	3	2	4	4	4	52
4	2	2	2	1	2	4	4	49
3	3	3	2	2	3	4	2	42
4	3	2	2	3	4	5	3	56
5	5	3	4	3	4	4	4	63
3	1	2	3	2	3	5	5	51
5	5	3	5	4	5	5	4	66
4	3	5	5	5	5	4	5	69
3	2	2	2	2	2	5	4	55
4	2	3	3	3	4	4	5	51
3	3	4	3	3	3	3	3	50
4	3	3	3	3	4	4	5	62
4	3	3	3	4	5	4	4	62
5	2	3	3	2	4	2	4	48
4	4	3	3	4	4	5	4	64
4	1	1	1	1	3	3	3	44
5	4	3	3	4	5	4	5	66
5	5	3	3	2	5	5	5	71
4	4	4	4	3	4	5	4	55
4	2	2	3	3	3	4	3	57
5	4	4	4	5	5	5	4	59
4	4	3	3	3	4	5	5	57
4	2	2	3	2	3	2	3	43
3	2	1	1	2	4	4	4	40
5	3	2	3	2	4	3	5	50
5	4	2	2	2	4	5	5	58
4	2	2	2	3	3	3	4	55
3	3	3	3	2	2	4	3	48
4	2	2	2	2	3	4	4	44
3	3	3	3	3	3	3	4	60
4	2	3	3	3	3	3	4	53
5	5	5	5	5	5	4	5	65
4	3	4	2	2	3	4	4	42
5	3	4	3	4	3	4	4	55
4	3	3	2	3	4	2	4	53
4	4	2	3	3	4	4	4	52
4	4	4	3	5	5	5	4	56
5	2	2	3	1	5	5	5	55
4	4	3	3	4	4	4	4	44
3	2	2	2	2	2	3	3	37
4	3	2	3	4	4	3	5	55
4	2	3	2	2	2	4	4	52
5	3	3	2	3	3	3	4	51
3	2	2	2	1	3	5	4	38
2	1	1	1	1	3	3	3	42
5	1	3	4	1	5	3	4	55
4	3	3	3	2	3	3	4	51

3	1	1	1	1	2	5	3	50
4	5	5	5	5	5	5	5	62
3	3	3	3	3	3	2	4	50
5	2	4	4	4	3	5	4	56
4	3	3	3	4	4	3	4	48
3	1	1	1	1	2	3	3	39
5	4	2	2	4	3	2	5	50
4	3	4	4	4	3	4	4	59
3	3	3	3	3	4	4	4	59
5	4	4	2	2	4	5	5	59
3	3	4	4	5	5	5	5	47
5	5	5	5	5	5	5	5	70
5	4	3	3	3	4	4	5	47
1	3	1	2	3	4	4	3	57
4	4	3	3	4	4	4	4	49
4	2	2	2	2	2	2	5	36
5	1	1	1	1	3	3	3	43
4	4	2	3	3	5	4	4	55
2	1	1	1	1	2	4	2	37
4	4	4	4	4	4	4	4	55
5	4	3	3	3	4	3	5	53
5	1	1	1	1	3	3	1	31
3	2	1	1	2	2	3	5	48
4	3	3	3	3	4	4	4	55
3	1	1	1	1	2	4	3	45
5	3	3	2	4	4	3	4	55
5	2	3	3	3	4	4	5	64
3	2	3	3	2	4	4	4	50
4	3	3	3	4	4	5	3	51
4	2	3	4	3	2	4	4	60
1	1	1	1	1	2	5	3	39
2	4	4	4	4	4	4	3	54
5	1	1	1	1	2	3	3	39
5	4	4	4	4	4	4	5	64
5	4	4	5	4	5	4	5	70
5	3	5	4	3	5	5	5	63
4	3	2	2	3	4	5	5	52
4	4	4	4	4	4	5	5	61
5	1	1	2	2	2	2	4	46
5	3	4	4	4	4	5	5	53
3	2	2	2	3	3	4	4	55
4	3	2	3	3	4	5	4	53
4	1	2	2	2	3	4	2	47
4	4	4	4	4	4	4	4	70
5	2	2	2	3	4	3	3	48
3	1	1	1	1	3	4	4	42
5	2	2	2	3	4	4	3	49
4	1	1	1	1	1	3	3	33
4	2	2	2	3	4	4	5	49
4	3	2	2	2	2	4	3	29
4	1	2	2	2	2	4	3	52
3	2	2	2	2	2	4	3	38
3	1	1	1	1	1	3	3	27
1	4	4	4	2	5	3	5	58
4	5	5	5	5	5	2	4	46

4	3	2	2	2	5	2	3	45
5	2	3	3	3	5	5	5	51
3	2	2	2	1	2	1	3	38
4	1	2	1	2	3	1	3	37
4	4	4	4	4	4	4	5	47
3	1	1	1	1	1	3	4	44
5	1	1	1	1	1	2	3	43
4	3	3	3	4	4	5	5	63
4	4	4	4	4	4	5	5	54
3	4	3	2	4	4	5	5	55
4	3	2	2	2	4	4	4	63
3	3	3	3	3	4	3	3	39
4	2	1	1	2	4	3	4	39
4	2	2	2	2	5	3	3	53
4	3	3	3	3	3	3	4	40
3	1	1	1	1	1	4	4	37
5	3	3	3	3	3	5	5	60
5	5	5	5	5	5	5	5	71
3	3	2	3	1	4	4	4	45
2	2	3	3	2	2	3	4	48
3	3	3	4	3	4	2	5	50
4	3	3	3	3	3	4	4	48
3	1	3	3	1	2	5	5	40
3	2	2	2	2	4	3	2	39
4	4	3	3	4	4	5	5	56
3	3	2	3	3	3	4	4	44
4	4	2	3	3	3	5	5	63
3	3	3	3	3	3	3	3	46
2	3	3	3	2	3	5	5	51
4	4	2	4	4	4	4	4	55
4	3	4	4	4	5	4	1	51
4	2	3	3	2	4	4	4	51
3	3	3	3	3	3	2	4	44
3	3	3	3	3	3	3	4	60
4	5	5	5	5	5	4	4	69
3	3	3	3	3	3	4	3	50
5	4	4	4	4	5	5	5	69
5	3	4	4	4	5	5	5	65
5	3	4	4	4	4	4	4	58
4	3	3	3	2	4	5	5	52
4	3	3	2	2	4	5	4	45
3	2	2	3	3	3	3	3	43
3	3	3	3	3	3	4	4	51
4	3	2	3	2	4	4	4	62
2	2	2	2	2	2	3	3	44
4	4	4	4	4	4	5	5	54
2	2	2	2	2	3	3	3	41
3	1	1	1	1	1	3	5	45
3	2	2	2	2	3	4	4	43
3	3	3	3	3	3	4	3	42
4	2	2	2	2	3	4	4	58
3	1	1	1	1	1	3	3	48
4	2	1	1	2	3	5	4	46
3	1	1	1	1	5	3	5	46
3	2	2	2	2	3	4	4	53

3	3	5	4	5	5	5	5	53
1	1	2	1	1	5	5	5	31
5	5	5	5	5	5	5	5	65
4	3	2	3	3	4	5	4	61
2	1	2	2	2	4	4	3	61
4	2	2	2	2	4	2	2	38
4	4	3	4	4	3	5	5	62
1	4	4	3	3	5	5	4	69
3	2	2	2	2	2	4	3	42
5	5	5	5	5	5	5	5	65
5	4	4	4	4	5	5	4	46
5	5	5	4	5	5	5	5	70
5	4	3	3	3	4	5	5	51
3	4	4	4	4	4	5	5	60
5	1	3	2	5	5	5	5	67
4	2	2	1	1	3	3	3	47
4	5	5	5	5	5	5	5	68
3	3	3	2	3	4	4	4	44
4	5	5	5	5	5	5	5	73
5	3	3	4	4	4	4	5	56
5	5	5	5	5	5	5	5	73
4	3	2	2	3	4	3	3	39
3	3	3	3	3	3	3	3	59
5	1	1	1	3	1	4	4	59
4	4	3	3	2	4	5	5	54
5	2	2	3	1	5	3	5	59
5	1	3	3	2	2	3	3	46
3	3	2	2	2	3	4	3	49
5	4	2	4	5	5	5	5	65
3	1	1	1	1	2	4	4	42
4	3	3	3	3	4	4	4	39
5	3	3	3	5	4	5	4	63
3	4	5	5	4	4	5	5	58
4	4	4	4	4	4	4	4	50
5	2	4	4	3	4	4	4	54
2	3	3	3	3	4	3	3	45
5	2	1	1	1	2	4	4	42
5	2	1	2	3	4	5	5	51
3	1	1	1	1	4	3	3	38
3	3	2	2	2	5	4	4	56
4	4	3	3	4	4	4	4	48
4	2	2	2	2	3	4	4	46
5	2	3	3	2	4	5	5	58
5	2	3	2	1	4	5	5	54
3	3	2	1	2	3	4	4	60
3	3	3	3	2	3	3	3	52
4	1	3	3	3	4	4	4	51
3	2	3	3	1	1	4	4	50
4	3	4	4	4	4	4	4	52
4	2	2	3	3	2	2	2	44
4	4	4	4	5	4	4	5	59
5	4	2	3	3	5	5	5	51
5	5	5	5	1	5	1	5	51
5	4	5	5	5	5	5	5	71
3	1	5	3	5	5	3	5	66

4	5	5	5	5	5	5	5	69
4	4	4	4	4	4	4	4	43
2	3	3	2	1	3	3	3	44
5	4	4	5	5	5	5	5	75
4	4	4	4	4	4	5	4	42
4	4	4	4	5	4	2	4	52
3	1	3	3	3	3	3	3	49
5	4	4	4	5	4	5	4	52
5	3	2	4	4	4	4	4	58
5	5	5	5	5	5	5	5	71
5	4	4	4	4	5	4	5	64
4	3	4	5	5	5	5	1	57
5	4	4	3	4	4	4	4	56
4	4	4	3	4	4	3	4	49
5	3	4	4	4	4	3	3	57
3	3	2	3	3	3	3	3	39
5	3	5	5	4	5	5	5	48
3	3	3	3	3	3	3	3	35
4	4	4	3	3	4	3	4	45
5	3	3	4	4	2	4	4	65
4	5	5	5	5	5	5	5	57
3	4	4	3	4	4	4	3	40
5	1	3	5	5	5	5	1	73
3	4	5	4	4	4	4	4	43
5	3	4	5	5	4	4	5	45
4	3	4	4	4	4	4	4	51
5	3	3	3	3	4	5	3	44
4	5	5	5	5	5	5	5	64
4	3	2	2	3	4	2	3	46
5	5	4	3	5	5	4	5	61
5	5	5	5	5	5	5	5	59
3	3	3	3	3	3	3	3	41
4	4	4	4	4	4	4	4	44
3	4	4	4	4	4	4	4	50
3	4	4	4	3	4	3	4	58
3	3	4	4	3	5	5	3	49
4	4	4	4	4	4	4	4	47
3	4	4	4	4	4	4	4	52
3	3	3	4	4	3	3	4	54
4	3	3	3	4	4	4	3	50
1	1	1	1	5	1	5	1	47
3	3	3	3	3	3	4	3	44
5	5	5	5	5	5	3	5	58
3	4	4	3	3	3	3	3	52
3	3	3	3	3	3	3	3	35
3	3	3	3	3	3	3	3	48
4	4	4	4	4	4	4	4	52
3	4	4	4	2	4	4	4	60
4	4	3	3	3	3	4	3	38
4	4	3	5	5	5	5	5	48
2	4	4	4	5	5	5	5	43
4	4	4	4	4	4	4	4	54
5	3	3	4	5	4	5	5	64
4	4	5	5	4	4	4	4	49
5	4	5	5	5	4	4	5	67

4	4	4	4	4	4	4	3	61
3	3	3	3	3	4	4	4	52
3	4	4	4	3	4	3	4	55
3	4	4	4	5	4	5	4	56
3	4	4	3	4	4	4	3	47
3	3	3	4	3	4	3	3	42
2	4	4	4	3	4	4	4	38
4	3	5	5	4	5	5	5	52
5	4	4	4	4	5	4	4	43
3	4	4	4	4	2	4	4	66
3	3	4	4	3	4	4	4	44
3	3	4	4	4	4	4	4	60
4	4	4	4	4	4	4	4	52
3	5	5	5	5	4	5	5	48
3	5	5	4	4	4	4	4	45
3	5	4	4	4	4	3	5	57
3	4	4	4	5	4	5	4	72
4	4	4	4	4	2	4	4	59
3	3	3	3	3	3	3	3	59
3	4	4	4	2	4	4	4	55
4	5	5	5	4	5	4	4	50
5	5	5	5	5	5	5	5	67
4	3	3	4	4	4	4	4	56
4	5	5	5	5	5	5	5	61
3	4	4	4	2	3	3	3	38
4	4	4	4	4	3	4	4	61
4	4	4	4	4	2	4	4	58
3	3	4	2	2	4	4	3	35
4	4	3	4	4	4	4	4	50
4	3	4	4	4	4	4	4	45
4	3	4	4	4	5	5	5	57
4	4	4	4	4	4	4	4	52
4	4	4	4	5	4	5	4	72
5	5	5	5	5	5	5	5	67
2	4	4	4	4	4	4	4	48
3	4	4	4	4	4	5	4	42
4	4	3	5	5	5	3	4	58
3	4	4	4	3	5	3	3	51
2	4	2	4	3	4	4	4	54
2	5	5	5	5	4	5	5	39
3	2	3	3	3	3	3	3	36
4	4	4	4	4	4	4	4	54
3	3	3	3	3	3	3	4	52
4	3	4	4	3	4	4	4	44
4	4	4	4	4	2	4	2	49
4	4	4	4	5	4	4	4	62
3	4	4	4	3	4	4	4	49
5	4	5	5	5	5	5	5	58
4	4	4	4	5	5	4	4	73
3	3	3	4	4	4	4	3	52
3	4	4	3	3	4	3	3	44
4	3	3	3	5	3	3	3	49
5	4	4	4	5	5	4	5	49
2	4	4	3	3	3	3	3	44
4	3	4	3	3	4	3	3	44

4	4	5	5	5	5	5	5	59
4	4	4	5	5	4	5	4	63
5	2	4	4	3	4	4	4	66
5	5	5	5	5	5	5	5	67
4	3	4	4	3	4	4	4	49
3	3	4	3	5	5	3	3	53
2	3	3	4	4	4	4	3	42
3	4	4	3	3	4	3	3	38
4	3	5	5	5	5	5	5	65
4	3	4	4	4	5	4	3	43
4	4	4	3	3	3	3	3	47
3	4	4	4	4	4	4	4	49
3	3	3	3	3	3	3	3	49
5	4	5	5	5	5	5	5	57
4	1	1	5	5	4	5	4	71
5	5	5	5	5	5	5	5	73
5	4	4	4	4	4	4	4	43
4	4	3	3	4	4	3	4	58
4	3	4	4	4	4	4	4	57
3	5	5	5	4	3	5	3	43
4	4	4	3	3	4	3	4	48
4	3	3	3	4	3	3	4	63
3	4	4	4	5	4	4	4	53
3	3	3	3	3	3	3	3	42
4	5	5	4	5	5	4	5	57
2	3	3	3	3	3	3	3	34
3	4	3	3	3	4	3	3	51
3	4	4	4	3	4	4	3	50
4	5	5	5	5	5	5	5	53
4	4	4	4	5	5	4	5	53
3	4	4	4	4	4	4	4	56
4	3	5	4	5	1	4	4	59
3	2	2	3	4	4	3	4	49
4	4	4	3	3	4	3	4	39
4	3	3	3	3	3	3	3	42
4	4	4	4	2	4	4	4	48
4	4	1	5	5	4	5	4	66
3	4	4	4	4	4	4	4	53
3	3	3	4	5	3	4	3	54
3	4	4	3	3	3	3	3	47
3	3	3	3	3	3	3	3	60
3	3	3	2	3	3	2	3	43
4	2	3	3	3	4	3	4	40
4	5	5	5	5	5	5	4	57
5	4	4	4	3	4	4	4	57
2	4	4	4	3	4	4	4	51
3	3	3	3	3	3	3	3	46
3	4	4	4	4	4	4	4	39
4	3	5	5	5	5	5	5	36
5	4	4	4	4	4	4	4	52
4	3	3	4	4	4	4	4	59
3	4	5	4	3	5	4	4	47
3	3	3	3	3	3	3	3	53
4	3	3	2	3	3	2	3	48
4	4	4	4	4	4	4	4	48

4	3	4	4	4	4	4	4	53
2	3	3	3	3	3	3	3	46
5	4	4	5	5	5	5	5	48
4	4	4	4	5	4	4	4	57
5	5	5	5	5	5	5	5	72
5	4	4	4	4	5	4	5	54
4	3	3	3	4	3	3	3	48
3	3	3	5	1	5	5	5	54
5	4	4	4	4	4	4	3	62
4	3	4	3	3	5	3	3	45
1	5	5	5	5	5	5	5	51
3	5	4	4	4	5	4	5	49
4	4	4	4	4	4	4	5	50
4	4	4	4	5	5	4	5	60
1	3	3	2	4	2	2	2	40
5	5	5	5	4	5	5	5	58
5	5	5	5	5	5	5	5	51
5	5	5	5	5	5	5	5	66
3	3	3	3	3	3	3	3	55
5	5	5	5	5	5	5	5	56
4	4	4	4	4	4	4	4	41
3	3	3	3	3	3	3	3	54
3	3	3	3	3	3	3	3	55
5	3	5	4	3	5	4	4	50
3	3	3	3	3	3	3	3	47
3	3	3	3	3	3	3	4	51
3	5	4	5	5	5	5	4	50
2	3	3	3	4	3	3	5	56
5	3	4	5	3	4	5	1	46
4	4	3	3	3	4	4	4	50
4	4	4	4	5	5	5	5	67
3	4	4	4	4	4	3	3	46
4	4	4	4	4	4	2	4	66
3	5	5	5	5	5	4	5	47
3	4	2	2	3	4	4	4	54
4	4	4	5	5	5	2	5	49
3	4	4	4	4	3	5	4	55
4	4	4	4	5	4	2	4	53
4	4	5	5	5	5	3	5	51
4	4	3	3	3	3	3	3	56
3	5	5	5	5	4	4	4	45
3	4	4	4	4	3	3	3	44
4	4	5	4	4	4	5	4	54
4	4	4	4	4	4	4	4	52
5	5	5	5	5	5	5	5	59
5	5	5	5	5	1	5	5	61
4	3	3	3	4	3	3	4	62
5	1	5	5	5	5	1	5	63
3	5	5	5	5	5	5	5	65

Totle_score _WFC	Totle_score _WFE	Totle_score _JE
36	8	25
24	22	22
32	18	24
27	12	21
22	22	35
25	30	32
24	21	33
21	24	28
24	19	22
14	27	35
26	19	31
21	20	32
20	24	26
22	15	31
25	18	26
30	23	26
25	24	26
16	18	28
16	24	30
20	22	25
20	20	28
40	30	27
18	21	27
15	23	28
23	20	24
20	24	31
21	24	31
12	21	19
24	18	23
19	18	28
30	19	25
24	18	30
25	14	28
32	18	24
20	23	33
15	30	30
25	15	31
16	17	29
25	19	27
24	18	35
17	30	29
27	15	23
24	18	21
19	26	25
21	19	27
24	10	26
24	27	27
24	22	27
19	18	22
19	19	22
20	30	27

33	23	28
30	25	31
23	21	24
29	17	23
21	20	26
25	22	28
24	17	21
16	20	25
18	17	25
34	16	20
30	14	26
24	18	23
25	23	35
24	22	27
24	12	26
18	20	31
24	18	26
25	20	28
20	23	27
24	18	26
25	18	23
28	25	30
24	18	24
28	17	22
24	24	21
36	18	26
14	18	23
21	18	21
40	9	29
22	23	21
24	22	26
16	21	28
28	20	23
19	12	33
23	18	27
8	28	35
24	19	23
23	22	26
15	24	24
34	22	29
24	23	28
40	24	27
16	24	34
20	23	32
26	18	23
33	21	24
23	22	30
14	23	24
23	23	28
29	21	31
26	28	32
33	12	19
23	17	21
17	21	32
29	16	22

27	24	21
29	28	31
20	16	30
32	18	23
31	20	30
10	17	35
25	18	21
31	16	23
28	21	30
24	18	22
27	16	21
24	17	28
22	18	31
26	18	21
29	25	35
28	13	27
24	24	23
29	20	31
32	22	21
25	18	29
27	21	28
23	17	32
16	30	35
21	14	23
33	24	23
19	18	35
17	25	35
28	12	21
19	17	28
15	29	35
24	18	21
30	19	24
30	19	22
34	24	22
19	15	33
40	10	16
34	21	23
25	20	30
15	19	32
30	25	23
28	11	14
22	23	27
28	13	35
18	27	35
22	21	27
37	16	21
15	20	32
25	19	22
25	20	33
8	29	35
20	21	28
8	30	35
29	21	26
27	18	30
24	18	21

34	13	16
24	18	28
27	23	31
21	23	31
19	24	33
24	18	28
30	18	24
22	23	28
38	19	23
18	21	25
16	24	31
20	21	29
19	21	23
36	24	16
14	22	28
23	15	22
26	18	24
36	24	20
24	14	21
16	22	31
24	18	34
17	18	28
25	26	35
24	24	22
21	25	29
20	26	34
26	8	35
20	24	35
14	24	31
21	23	28
21	24	32
20	18	28
30	19	25
25	18	25
16	25	35
16	28	35
28	19	21
17	22	35
15	22	31
24	19	25
12	28	30
23	18	28
21	15	24
25	19	22
20	22	29
29	24	28
22	23	28
19	19	20
22	16	24
18	23	25
22	17	21
28	18	21
26	21	25
27	19	32
24	20	23

27	17	26
20	24	34
37	9	19
24	23	27
32	18	21
9	30	30
20	18	18
22	11	33
16	16	28
16	24	27
24	18	24
24	11	28
19	22	27
18	16	24
23	20	33
32	15	27
22	23	27
38	21	21
25	17	28
32	20	29
22	19	28
18	20	35
34	26	33
24	18	21
28	19	24
40	18	11
21	23	28
27	17	31
25	24	35
24	18	24
8	12	22
24	23	30
18	20	29
20	21	35
34	24	28
16	28	30
25	16	24
24	18	21
16	18	25
30	24	33
29	24	24
27	23	22
19	18	27
22	26	33
12	24	35
14	22	27
28	20	23
35	17	30
23	18	24
22	26	32
16	20	29
17	21	28
19	24	28
26	18	33
21	18	26

24	17	28
34	30	31
20	18	25
22	12	33
35	24	32
29	17	21
13	19	33
21	24	30
15	23	30
24	18	26
28	21	25
16	24	28
21	13	28
27	18	28
28	22	35
24	18	21
23	21	26
22	18	31
24	18	21
21	20	24
20	19	21
12	18	32
11	21	30
8	6	35
21	17	21
19	18	26
29	15	30
22	19	30
25	30	34
20	19	20
11	28	18
22	24	24
15	18	30
24	14	11
19	24	21
21	24	21
23	24	15
15	22	18
18	24	14
18	18	25
20	28	26
20	24	16
28	17	15
17	22	17
25	18	22
35	30	22
37	19	11
10	10	32
21	21	22
20	18	29
20	24	21
21	6	13
25	23	7
11	27	33
24	21	18

25	17	17
22	22	20
14	22	14
23	17	12
17	23	14
21	20	21
19	25	27
33	16	14
27	23	19
22	25	22
23	17	22
19	24	16
20	18	22
17	23	24
21	17	22
35	23	14
8	24	28
35	24	13
21	18	26
19	22	22
19	20	18
16	24	30
8	24	29
20	21	18
21	13	15
21	23	26
8	6	19
23	21	21
24	18	19
17	30	20
8	24	27
8	29	31
20	15	24
20	18	20
19	13	28
22	19	21
8	30	35
21	18	24
22	23	26
28	22	14
17	17	27
23	22	28
16	24	20
19	18	14
20	16	20
23	13	10
26	18	20
19	18	18
28	25	15
23	30	24
27	16	19
20	17	22
24	19	7
24	18	13
17	22	25

25	27	13
18	20	27
28	17	13
19	18	19
25	20	16
34	22	9
22	20	21
28	24	8
20	19	17
30	18	14
28	17	10
40	16	7
29	20	7
14	19	29
12	19	22
26	18	14
16	28	29
24	10	11
24	18	13
24	18	20
16	18	25
21	21	21
19	23	17
24	25	28
16	25	15
21	11	18
22	21	20
31	23	30
23	18	21
16	20	24
28	12	14
28	18	15
23	20	22
25	15	13
24	23	21
21	18	25
23	21	19
18	29	26
16	19	18
19	24	25
28	21	21
30	18	18
27	19	17
11	30	24
28	24	13
24	24	24
34	6	14
17	22	27
24	20	12
30	22	21
24	27	22
22	23	11
16	24	33
27	22	16
26	22	22

22	23	11
20	30	29
23	19	23
25	18	17
22	24	23
25	11	13
20	21	14
12	20	26
26	17	8
32	19	14
16	30	20
26	23	20
22	22	19
15	25	21
17	21	20
10	20	26
21	15	14
24	18	16
16	23	25
28	16	23
27	15	15
21	18	23
21	14	21
25	18	20
26	23	9
24	22	16
27	17	13
40	30	7
30	19	23
17	18	21
27	18	8
32	16	7
16	20	10
27	18	21
22	19	13
24	19	16
28	23	12
24	16	20
24	17	16
25	16	14
24	18	23
23	24	18
16	20	27
25	20	18
28	12	12
19	18	26
19	22	19
23	21	15
25	24	21
17	21	19
24	18	21
40	30	21
19	24	24
25	16	7
20	18	20

24	18	24
25	17	17
27	18	21
8	27	35
26	21	14
25	22	31
23	16	23
27	23	15
16	24	7
13	18	26
8	30	34
26	15	12
25	23	20
30	18	12
17	17	20
26	18	23
17	18	21
20	22	20
22	20	20
16	24	25
20	27	17
25	21	19
34	17	15
17	24	25
23	20	22
21	17	16
16	21	23
20	22	22
24	18	22
27	20	13
12	26	35
17	28	20
15	21	28
26	15	14
21	24	21
8	24	21
25	18	18
28	17	14
16	20	13
17	20	13
32	19	16
23	15	17
24	13	7
32	24	23
28	22	11
8	26	27
21	18	20
15	21	19
30	14	10
20	22	17
22	16	27
12	22	27
21	21	20
34	21	10
23	26	22

24	22	23
23	22	23
21	23	24
22	27	25
23	21	22
10	22	16
22	18	18
27	19	12
24	17	21
11	24	29
24	26	19
18	22	18
15	30	20
24	20	13
26	21	13
24	18	23
15	18	19
20	22	23
27	20	20
16	21	7
24	18	21
24	18	20
24	30	24
18	19	18
23	18	11
32	24	19
17	30	21
25	10	12
38	20	11
10	22	15
23	19	22
25	19	14
34	25	14
16	20	24
17	18	19
28	21	12
19	20	19
40	9	13
24	19	15
25	14	23
27	18	20
25	23	12
23	24	15
8	30	30
19	19	24
29	18	18
35	14	20
24	18	21
16	20	28
24	16	20
31	22	18
14	17	21
24	19	7
27	20	20
20	21	25

20	18	18
13	20	23
27	15	22
18	23	24
26	20	15
29	11	25
24	23	31
24	18	24
8	20	13
8	15	20
20	20	23
27	20	20
18	17	19
14	19	24
21	20	28
11	28	19
25	22	25
24	18	16
40	16	33
24	24	26
23	20	22
19	22	19
17	20	16
8	30	23
16	21	21
19	19	22
24	18	16
24	18	17
31	17	28
24	20	19
16	19	15
32	18	18
24	18	19
22	21	24
23	25	32
29	15	28
19	19	16
17	24	22
22	26	29
15	21	24
24	20	19
13	23	26
22	19	20
24	18	24
13	27	19
17	18	19
21	21	24
20	18	20
39	22	24
24	18	28
8	9	23
18	26	28
8	30	21
25	21	26
22	22	25

24	20	28
8	30	17
28	18	16
23	16	22
29	24	26
12	22	33
23	21	28
26	15	25
12	18	24
24	16	20
24	18	27
32	19	28
29	22	28
24	17	32
20	21	20
20	21	20
22	17	25
8	30	16
24	18	18
25	21	26
21	18	16
22	18	22
21	18	24
14	24	29
20	14	21
20	19	21
8	18	15
24	18	25
20	20	23
28	18	25
20	22	20
29	18	32
24	20	24
22	18	20
40	20	31
29	15	26
32	24	25
20	24	22
22	18	21
14	26	23
15	23	19
9	30	22
20	21	25
32	20	33
33	19	18
24	22	24
17	17	22
30	19	26
19	30	31
24	18	17
20	20	26
24	15	20
36	24	29
27	19	30
16	23	20

18	26	28
22	24	24
26	20	30
32	24	28
21	22	22
11	24	24
28	19	18
11	26	23
21	20	30
16	18	16
32	11	29
24	18	25
21	21	20
33	20	20
16	19	21
24	6	27
23	18	16
16	21	22
23	25	24
24	14	26
14	23	22
22	22	25
20	24	21
20	30	24
35	9	25
19	24	22
17	21	23
28	18	23
23	15	28
20	20	25
39	15	26
8	22	16
33	24	31
32	20	24
27	20	26
20	21	24
28	24	29
19	20	25
29	20	28
30	14	28
14	29	27
20	23	18
22	23	27
20	20	22
20	18	22
28	19	28
15	22	29
23	22	22
30	18	23
31	17	22
30	22	28
20	21	23
20	24	23
24	18	23
20	18	25

20	30	27
27	14	25
19	15	24
34	15	27
25	19	25
26	17	29
37	14	26
23	12	30
24	18	22
35	21	28
22	14	27
19	13	22
24	18	28
26	18	27
17	30	19
16	22	23
16	21	22
24	19	23
17	20	26
24	14	22
20	25	27
22	18	28
26	18	28
24	18	25
16	27	25
24	8	29
18	19	27
24	18	26
16	25	23
24	22	18
8	30	15
25	24	19
15	21	27
21	24	25
27	19	16
23	17	20
22	23	19
20	20	24
24	19	22
24	20	15
16	18	23
17	21	27
28	18	17
18	14	24
17	22	25
19	18	24
24	24	21
21	18	20
20	21	27
15	18	29
40	9	11
16	13	26
8	24	34
24	18	23
9	24	33

23	19	22
23	21	21
23	20	19
24	19	18
19	18	19
40	20	11
23	18	23
25	22	19
35	20	11
20	22	21
25	18	20
27	17	19
16	13	27
23	18	18
27	23	20
28	18	17
28	17	17
8	30	35
15	22	28
27	18	18
31	18	13
19	21	27
31	22	17
28	20	17
35	22	15
14	22	28
19	24	25
14	15	28
16	22	26
16	24	28
8	21	35
24	18	23
31	18	17
26	17	18
24	18	20
32	18	16
21	22	23
24	18	17
29	21	17
24	20	17
23	13	21
18	22	28
26	18	16
21	18	21
24	23	25
26	22	19
17	21	27
17	26	28
17	20	28
39	21	12
29	24	17
26	21	16
17	24	28
25	20	17
17	18	26

8	24	31
24	18	21
26	20	19
16	16	27
25	15	20
26	16	18
16	25	27
22	19	22
19	17	25
24	18	21
12	22	31
20	14	26
40	9	10
32	15	13
16	16	22
14	28	28
32	18	18
27	18	18
8	30	35
20	25	22
20	18	23
25	11	17
35	21	14
23	20	26
21	26	22
19	15	24
18	18	26
24	21	18
26	24	20
23	21	22
28	19	17
28	15	20
30	15	15
34	22	12
32	24	18
18	17	22
8	30	34
29	26	16
16	30	27
20	17	27
26	18	19
20	16	24
24	23	21
24	18	23
18	15	21
24	20	22
32	24	17
31	24	20
24	18	19
25	15	19
25	16	22
21	22	22
26	24	19
15	30	29
16	20	26

22	11	18
20	23	24
25	19	21
31	21	16
19	22	26
12	19	27
26	18	19
30	19	17
32	24	17
8	30	35
24	18	23
16	24	26
24	18	21
19	30	27
32	24	18
33	18	14
13	22	28
23	27	21
16	24	25
21	26	23
21	21	23
21	14	18
23	15	20
18	21	24
26	22	21
21	14	24
23	20	20
20	26	24
11	20	30
16	24	28
26	19	21
25	18	20
24	20	18
25	16	17
19	12	22
18	16	21
31	18	19
31	19	18
27	18	18
25	21	21
19	18	25
17	23	27
26	21	20
8	30	35
23	19	21
13	20	30
25	16	22
28	25	17
29	21	14
17	24	27
24	18	20
29	17	21
23	22	23
21	24	25
36	14	10

27	11	14
22	17	21
21	16	22
25	21	18
18	20	21
25	9	21
40	18	12
18	18	24
22	16	22
33	23	17
26	18	19
20	19	22
17	30	27
27	18	21
11	27	31
23	23	32
32	20	19
22	19	24
23	16	22
20	21	25
15	24	26
22	25	20
16	24	27
32	18	13
14	27	28
15	26	28
17	20	28
24	18	20
11	24	31
16	20	27
30	24	17
26	18	18
23	22	22
20	26	24
26	19	19
29	20	20
31	24	19
24	18	22
26	24	21
8	26	34
23	22	22
18	24	25
21	20	21
20	17	24
15	25	30
21	19	23
18	24	26
27	20	16
17	23	24
30	16	19
22	19	21
29	14	19
32	12	13
19	30	21
24	22	21

34	18	14
8	22	35
24	18	21
20	27	26
21	19	24
33	21	12
24	28	22
18	24	26
20	19	24
21	20	26
12	13	31
8	30	35
17	19	26
23	7	20
18	23	26
26	21	17
31	23	13
16	24	25
38	14	12
16	16	28
18	26	25
34	20	11
29	18	16
20	23	24
35	18	13
20	29	23
21	22	24
22	18	22
19	24	25
23	24	22
35	13	14
16	12	27
35	21	12
16	30	29
11	29	31
13	26	30
20	24	24
13	22	30
34	20	14
16	24	29
27	15	20
21	18	24
28	18	16
16	24	28
25	24	19
31	18	15
22	23	20
37	17	11
23	24	22
31	24	18
30	24	16
29	18	17
40	16	11
20	20	27
8	24	31

26	22	19
17	29	26
32	18	13
29	15	13
17	29	29
39	21	12
40	18	10
19	24	27
16	24	30
20	16	27
23	18	21
20	18	22
30	20	17
20	13	19
23	19	22
40	14	13
24	26	25
8	30	35
23	16	21
27	16	19
19	17	24
22	20	23
31	20	20
22	18	17
16	20	28
28	21	22
23	22	25
24	18	21
27	17	24
18	21	26
17	24	25
22	22	22
24	18	21
24	18	22
8	24	33
24	18	22
12	30	31
13	25	30
17	30	27
22	19	25
22	24	23
26	18	19
24	18	23
22	22	22
32	12	16
16	23	30
28	12	17
34	16	13
28	18	19
24	18	22
24	18	19
40	8	11
29	21	18
25	22	17
28	19	19

11	18	32
35	9	20
8	30	35
21	24	24
25	13	18
24	14	16
18	24	28
14	8	28
31	18	17
8	30	35
12	20	30
9	26	34
18	18	27
16	14	30
17	30	26
29	19	15
8	24	35
21	17	23
8	19	35
16	24	27
8	30	35
23	23	20
24	18	21
30	27	15
21	21	26
24	28	21
29	30	17
26	18	19
13	25	30
30	20	14
20	18	24
15	24	27
31	16	32
16	21	28
19	26	25
20	16	22
34	18	15
23	25	22
31	14	14
21	20	22
18	22	26
25	18	19
22	28	24
25	23	22
30	18	19
25	18	20
23	22	22
35	22	18
17	24	27
25	19	16
18	16	30
21	22	27
40	30	27
8	30	34
17	18	27

16	24	35
30	18	28
28	14	18
8	30	33
31	23	29
21	19	27
31	18	19
20	22	30
16	24	25
12	28	35
20	26	30
18	24	28
27	22	27
24	22	26
17	25	25
25	17	20
27	25	32
24	15	21
35	20	25
19	30	24
33	24	35
28	15	26
20	30	25
29	15	29
19	25	30
22	24	27
40	30	24
17	24	35
22	19	19
20	22	31
8	30	35
24	18	21
23	16	28
25	18	28
23	18	26
21	18	27
22	20	28
23	18	28
26	19	24
21	21	24
8	6	15
22	18	22
32	18	33
20	16	23
25	18	21
29	18	21
21	18	28
20	23	26
30	21	23
23	23	32
25	12	32
16	23	28
24	28	29
22	23	30
8	30	32

10	18	27
25	18	24
25	19	26
8	16	30
22	20	26
30	22	23
26	16	27
23	20	32
28	27	29
27	18	26
20	18	26
20	15	27
23	19	28
32	21	34
28	16	30
23	13	29
16	18	30
19	19	26
14	22	21
29	20	26
31	15	32
22	30	35
28	16	26
10	24	35
33	18	23
16	16	27
20	23	26
29	15	22
24	21	27
21	18	27
23	22	30
24	22	28
8	21	30
13	25	35
25	13	28
27	22	29
13	24	29
28	18	26
24	17	25
25	10	34
40	19	20
20	23	28
25	18	22
24	18	26
22	21	24
21	24	29
22	19	27
13	23	34
16	24	30
19	17	25
24	18	24
20	17	23
16	22	31
26	12	23
22	23	23

21	22	34
20	22	31
20	24	25
18	20	35
30	20	26
21	16	26
25	15	25
33	15	24
16	24	33
18	17	27
24	17	23
17	15	28
20	17	21
24	25	34
8	17	25
14	27	35
30	26	28
18	20	25
20	21	27
28	19	30
32	20	25
17	21	23
20	18	29
25	15	21
26	24	33
32	12	21
21	20	23
28	18	26
26	14	35
22	27	31
23	16	28
14	19	26
21	16	22
27	23	25
28	15	21
30	24	26
19	16	28
20	18	28
21	18	25
20	12	23
20	18	21
25	17	19
20	18	22
14	28	34
24	26	27
28	18	27
26	18	21
29	15	28
30	19	33
20	21	28
21	20	26
25	20	29
24	18	21
29	19	19
23	15	28

24	24	27
22	15	21
23	25	33
18	22	29
8	30	35
27	23	30
22	20	22
24	14	27
17	25	27
19	23	24
17	14	35
29	21	31
19	24	29
21	23	31
30	11	18
20	28	34
14	24	35
13	30	35
32	17	21
17	22	35
30	22	28
24	18	21
20	18	21
26	26	28
16	18	21
26	18	22
19	17	33
13	12	24
27	26	25
23	19	25
12	24	32
24	19	26
15	22	26
26	17	34
23	19	23
24	21	30
25	17	28
23	23	27
25	24	32
28	17	22
24	18	32
28	18	25
18	22	30
20	22	28
21	29	35
15	30	31
16	24	23
8	30	27
11	25	35
