# Supplementary material for: Relationship between job resources and job embeddedness among tertiary-level public hospital nurses: parallel mediating roles of work–family conflict and work–family enrichment
Source: Front Public Health. 2025 Jun 2;13:1527511. doi: 10.3389/fpubh.2025.1527511 (PMC12171369; doi:10.3389/fpubh.2025.1527511)
Supplement: Supplementary file 2 [file Data_Sheet_2.pdf]

伦理审查批件

初始审查意见为同意

**批件号**	KYLL20210827-1		
**项目（论文）名称**	基于 JD-R 模型的三级公立医院护士<br>工作要求-资源对幸福感的影响研究-以潍坊市为例		
**申办单位**<br>**参与单位**	潍坊市人民医院		
**承担科室/主要研究者**	护理部/陈丽英等		
**审查类别**	初始审查	**审查方式**	快速审查
**审查日期**	2021 年 8 月 27 日	**审查地点**	线上
**审查文件**	（见附表）		

审查意见

经医院医学科研伦理委员会对研究者资质、研究方案、临床科研项目知情同意书及相关资料审核，同意按照所批准的临床研究方案等开展本研究。

**年度/定期跟踪审查频率**	请于 2022 年 8 月 27 日前 1 个月提交年度/定期跟踪审查
**有效期**	2021 年 9 月 － 2024 年 8 月
**声明**	本伦理委员会是相对独立的，委员会的职责、人员组成、操作程序及记录均遵循《涉及人的生物医学研究伦理审查办法》《中华人民共和国人类遗传资源管理条例》和中国相关法律法规。所有出席委员会均在委员会有效任职期间。
**主任委员/副主任委员签名（签章）**	王少强
**伦理委员会**	**潍坊市人民医院医学科研伦理委员会（盖章）**
**日期**	**2021 年 8 月 27 日**

注：伦理批件批准 1 年内项目没有启动，则该批件自动失效。

伦理委员会地址：潍坊市奎文区广文街 151 号
联系电话：0536-8192680
E-mail: wfsrmyykyb@163.com

医学伦理委员会审查文件清单

项目名称：基于 JD-R 模型的三级公立医院护士工作要求-资源对幸福感的影响研究-以潍坊市为例

序号	文件名称	版本号	版本日期
1	科研伦理审查申请书	NA	2021 年 8 月 27 日
2	实施方案和相关依据资料	版本号：1.0	2021 年 8 月 27 日
3	临床科研项目知情同意书	版本号：1.0	2021 年 8 月 27 日
4	项目相关人员履历表	NA	2021 年 8 月 27 日
5	申请项目的可行性和安全性报告	版本号：1.0	2021 年 8 月 27 日
6	与本项目相关的管理制度和质量保障措施	版本号：1.0	2021 年 8 月 27 日
7	开展本项目的风险评估与应急预案	版本号：1.0	2021 年 8 月 27 日

伦理委员会地址：潍坊市奎文区广文街 151 号
联系电话：0536-8192680
E-mail：wfsrmyykyb@163.com
