# Supplementary material for: Relationship between job resources and job embeddedness among tertiary-level public hospital nurses: parallel mediating roles of work–family conflict and work–family enrichment
Source: Front Public Health. 2025 Jun 2;13:1527511. doi: 10.3389/fpubh.2025.1527511 (PMC12171369; doi:10.3389/fpubh.2025.1527511)
Supplement: Supplementary file 3 [file Data_Sheet_3.pdf]

三级综合性公立医院护士调查问卷

亲爱的白衣天使：

您好！本调查旨在了解护士的工作情况，所得信息仅供综合性研究之用。

问卷不必署名，答案无所谓对错！恳请您仔细阅读指导语后据实填写，凭您的第一感觉回答，不必过多思虑；不要漏掉任何一题。您的意见非常宝贵，谢谢您的支持！

单位全称：____________________

一、护士基本情况

序号	问题及选项	应答
1	您的性别：①男 ②女	
2	您的年龄：________	
3	最高学历：①高中/中专及以下 ②大专 ③本科 ④硕士及以上	
4	所在科室：<br>①内科 ②外科 ③妇产科 ④儿科 ⑤急诊科 ⑥其他（请注明）________	
5	技术职称：①护士 ②护师 ③主管护师 ④副主任护师 ⑤主任护师	
6	职务：①无 ②有（请注明）________	
7	编制情况：①编制内 ②合同制 ③退休返聘 ④其他（请注明）________	
8	本单位工作年限：<br>①<1 年 ②1-5 年 ③6-10 年 ④11-15 年 ⑤16-20 年 ⑥>20 年	
9	您觉得每天的工作强度：<br>①很轻松 ②不大 ③一般 ④较大 ⑤非常大	
10	您认为您的健康状况：<br>①非常不健康 ②不大健康 ③一般 ④比较健康 ⑤非常健康	

二、护士工作资源评价（请根据下表描述，选择与自身情况相符的选项，将符合程度填写在应答栏）

序号	问题及选项	应答
1	与护理管理者发生冲突<br>①从不 ②很少 ③有时 ④经常 ⑤绝大部分	
2	与科室的某些护士一起工作很难<br>①从不 ②很少 ③有时 ④经常 ⑤绝大部分	
3	与医生发生冲突<br>①从不 ②很少 ③有时 ④经常 ⑤绝大部分	
4	护理管理者的批评过多<br>①从不 ②很少 ③有时 ④经常 ⑤绝大部分	
5	同事之间缺乏理解和支持<br>①从不 ②很少 ③有时 ④经常 ⑤绝大部分	
6	我们能参与制定科室的规章制度<br>①从不 ②很少 ③有时 ④经常 ⑤绝大部分	
7	对科室今后的工作计划，我们能参与讨论并出谋划策<br>①从不 ②很少 ③有时 ④经常 ⑤绝大部分	
8	在我的工作中，做出决定需征求领导或同事的意见	

	①从不 ②很少 ③有时 ④经常 ⑤绝大部分	
9	我如何进行自己的工作，我能自己做出安排<br>①从不 ②很少 ③有时 ④经常 ⑤绝大部分	
10	护理工作的社会地位太低<br>①从不 ②很少 ③有时 ④经常 ⑤绝大部分	
11	晋升的机会少<br>①从不 ②很少 ③有时 ④经常 ⑤绝大部分	
12	好好工作，却不一定能得到相应的回报<br>①从不 ②很少 ③有时 ④经常 ⑤绝大部分	
13	我的工作十分单调、乏味<br>①从不 ②很少 ③有时 ④经常 ⑤绝大部分	
14	我的工作很有趣<br>①从不 ②很少 ③有时 ④经常 ⑤绝大部分	
15	我的工作丰富多彩，不乏味<br>①从不 ②很少 ③有时 ④经常 ⑤绝大部分	

三、护士工作家庭平衡评价（请根据下表行为描述，选择与自身情况相符的选项，将符合程度填写在应答栏）

序号	问题及选项	应答
1	1.回到家里后，工作中的烦恼或难题仍然缠绕着我。<br>①非常不同意 ②不同意 ③一般 ④同意 ⑤非常同意	
2	繁忙的工作使我没有时间参与家庭活动<br>①非常不同意 ②不同意 ③一般 ④同意 ⑤非常同意	
3	工作如此劳累，以至我没有精力处理家庭事务<br>①非常不同意 ②不同意 ③一般 ④同意 ⑤非常同意	
4	工作压力使我在家里变得急躁易怒<br>①非常不同意 ②不同意 ③一般 ④同意 ⑤非常同意	
5	个人烦恼或家庭问题常使我工作时心不在焉<br>①非常不同意 ②不同意 ③一般 ④同意 ⑤非常同意	
6	家庭压力使我在工作中变得急躁易怒<br>①非常不同意 ②不同意 ③一般 ④同意 ⑤非常同意	
7	家务杂事影响睡眠，进而影响工作<br>①非常不同意 ②不同意 ③一般 ④同意 ⑤非常同意	
8	对家庭尽责使我不能专心致志地工作<br>①非常不同意 ②不同意 ③一般 ④同意 ⑤非常同意	
9	我在工作中所做的事情有助于解决家里的实际问题<br>①非常不同意 ②不同意 ③一般 ④同意 ⑤非常同意	
10	我工作中掌握的技能在做家务活时派得上用场<br>①非常不同意 ②不同意 ③一般 ④同意 ⑤非常同意	
11	我在工作中所做的事情让我在家里变得饶有风趣<br>①非常不同意 ②不同意 ③一般 ④同意 ⑤非常同意	
12	同家人的沟通与交流有助于我解决工作中的问题<br>①非常不同意 ②不同意 ③一般 ④同意 ⑤非常同意	
13	从家人那里得到的爱和尊重使我在工作中充满自信<br>①非常不同意 ②不同意 ③一般 ④同意 ⑤非常同意	
14	家庭生活可以缓解紧张的工作压力<br>①非常不同意 ②不同意 ③一般 ④同意 ⑤非常同意	

四、护士工作嵌入评价（请根据下表描述，选择与自身情况相符的选项，将符合程度填写在应答栏）

序号	问题及选项	应答
1	我觉得我很依附于组织<br>①非常不同意　②不同意　③一般　④同意　⑤非常同意	
2	我很难做出离开组织的决定<br>①非常不同意　②不同意　③一般　④同意　⑤非常同意	
3	我十分喜欢目前的组织，不会离开<br>①非常不同意　②不同意　③一般　④同意　⑤非常同意	
4	我对组织感到厌倦<br>①非常不同意　②不同意　③一般　④同意　⑤非常同意	
5	我不能轻率地离开组织<br>①非常不同意　②不同意　③一般　④同意　⑤非常同意	
6	离开组织对我来说很容易<br>①非常不同意　②不同意　③一般　④同意　⑤非常同意	
7	我觉得我和组织紧密相连<br>①非常不同意　②不同意　③一般　④同意　⑤非常同意	
